# A prior-sampling conditional variational autoencoder for neuroimaging normative modelling: Benchmarking deep learning against statistical approaches

**DOI:** 10.1162/IMAG.a.1098

**Published:** 2026-01-12

**Authors:** Mai P. Ho, Yang Song, Perminder S. Sachdev, Lei Fan, Jiyang Jiang, Wei Wen

**Affiliations:** Centre for Healthy Brain Ageing (CHeBA), Discipline of Psychiatry and Mental Health, School of Clinical Medicine, Faculty of Medicine and Health, University of New South Wales (UNSW), Sydney, NSW, Australia; School of Computer Science and Engineering, Faculty of Engineering, University of New South Wales (UNSW), Sydney, NSW, Australia; Neuropsychiatric Institute (NPI), Euroa Centre, Prince of Wales Hospital, Sydney, NSW, Australia

**Keywords:** normative modelling, deep learning, UK Biobank, conditional variational autoencoders, precision psychiatry

## Abstract

Normative modelling in neuroimaging provides a powerful framework for quantifying individual deviations from expected brain measures as a function of relevant covariates. While earlier methods focused on analysing distinct variables in isolation, an increasing number of deep learning-based approaches are emerging to handle multiple response variables simultaneously. Conditional variational autoencoders (cVAEs) have previously been applied in this context and show promise for multivariate modelling. However, existing inference methods still face challenges in providing reliable probabilistic predictions, limiting their effectiveness as true normative models. In this study, we propose an enhanced cVAE-based framework that generates predictions directly from covariates through prior-sampling inference. This approach aligns with normative modelling principles while leveraging deep learning to handle high-dimensional data. We demonstrate the effectiveness of our approach using 195 imaging-derived phenotypes (IDPs), including morphometric features (cortical thickness, cortical volume, subcortical volume) and white matter hyperintensity (WMH) volumes, as a test case. Our dataset includes 8,551 normotensive and 18,180 hypertensive participants from the UK Biobank. We benchmarked our model against three well-established normative modelling techniques, including Generalised Additive Models for Location, Scale, and Shape (GAMLSS), Multivariate Fractional Polynomial Regression (MFPR), and Hierarchical Bayesian Regression (HBR), as well as the conventional posterior-sampling cVAE approach employed in existing autoencoder-based normative models. Through comparative analyses, our results show that the proposed cVAE-based framework achieves performance comparable with well-established normative models across various metrics, while appropriately capturing individual deviations associated with hypertension severity. Furthermore, our inference strategy demonstrates superior covariate sensitivity compared with those used in existing cVAE-based normative models, with deviations derived from our method showing better sensitivity to hypertension severity. Beyond predictive performance, our study also offers a comprehensive correlational mapping of the relationships between hypertension and brain structural damage. Taken together, this work highlights the promise of deep learning-based normative modelling for complex datasets such as neuroimaging, paving the way for personalised brain health assessment and early detection of neurological disorders.

## 1 Introduction

Normative modelling has recently emerged as a powerful approach in neuroimaging analysis for understanding individual variations in brain structure and function. Unlike traditional case–control studies that focus on group-level differences, normative modelling aims to establish a statistical norm of brain measures across populations, against which individual subjects can be compared (Marquand, Rezek, et al., 2016). This approach allows for the quantification of individual deviations from expected brain measures, potentially revealing subtle morphological differences that might go undetected using conventional techniques (Fraza et al., 2021). As a result, normative modelling holds great potential for early detection of neurological disorders and assessment of disease progression.

Several statistical approaches have been employed in normative modelling, each with distinct strengths and limitations in the context of neuroimaging analysis. Ge et al. (2024) provided a comprehensive comparison of these methods, highlighting widely used algorithms such as Generalised Additive Models for Location, Scale, and Shape (GAMLSS) (Rigby & Stasinopoulos, 2005), Hierarchical Bayesian Regression (HBR) (Lindley & Smith, 1972), Gaussian Process Regression (GPR) (Williams & Rasmussen, 1995), and Multivariate Fractional Polynomial Regression (MFPR) (Royston & Altman, 1994). For instance, GAMLSS has proven effective for modelling non-linearity through smoothing functions, as demonstrated in a lifespan brain development study (Bethlehem et al., 2022). By extending traditional generalised linear models, GAMLSS models all distribution parameters (location, scale, shape) as functions of explanatory variables, offering substantial flexibility in capturing complex relationships (Rigby & Stasinopoulos, 2005). However, it is not inherently designed to analyse multiple response variables simultaneously, limiting its capacity to capture the complex spatial correlations common in neuroimaging data (Habeck, 2010). Similarly, while HBR can model non-linear relationships through basis functions within a Bayesian hierarchical framework (de Boer et al., 2024), it analyses each feature separately, which limits its ability to capture shared patterns across brain regions. In modern implementations such as the PCNtoolkit (Marquand, Wolfers, et al., 2016), HBR adaptively controls model complexity using hyperpriors on smoothness parameters, helping to prevent overfitting and enabling the modelling of heteroscedastic noise. Despite these strengths, the computational cost of fitting and updating hierarchical posterior distributions increases rapidly with data size and dimensionality, making HBR less practical for large-scale neuroimaging applications. GPR, one of the earliest normative modelling approaches (Marquand, Rezek, et al., 2016), offers a principled Bayesian framework capable of modelling non-linear functions with quantified uncertainty. However, its computational complexity scales cubically with the number of data points ($O(n^{3}$)), rendering it even more computationally intensive than HBR. MFPR, known for its computational efficiency and generalisability in the CentileBrain study (Ge et al., 2024), offers flexibility in modelling non-linear relationships using fractional polynomial terms for multiple predictor variables. Through an automated stepwise procedure, it can systematically explore power transformations for multiple continuous covariates. However, like GAMLSS and HBR, MFPR models each brain measure separately, limiting its ability to capture interdependencies between different brain features. Furthermore, unlike GAMLSS, MFPR estimates only the conditional mean, restricting its ability to model higher-order distributional moments such as variance.

The limitations of traditional statistical approaches have motivated the exploration of deep learning methods for normative modelling. Deep learning has shown great promise in handling complex, non-linear relationships and simultaneous interactions among variables (LeCun et al., 2015), making it well suited for analysing the rich, high-dimensional information contained in brain scans. Although deep learning models can face scalability challenges, their flexible architectures and capacity for parallel computation often make them better equipped to manage such complexity compared with traditional methods. A notable example is the conditional autoencoder (cAE)-based normative model introduced by Pinaya et al. (2019), which jointly learns patterns across 104 neuroanatomical structures while incorporating age and sex as covariates. The cAE architecture consists of an encoder that compresses the input data into a latent representation, and a decoder that reconstructs the original input from this latent representation, conditioned on the relevant covariates. Building upon this work, Pinaya et al. (2021) introduced conditional adversarial autoencoders (cAAEs) for normative modelling, employing adversarial training with a discriminator to regularise the latent space distribution. Both models quantify deviations as reconstruction errors—the difference between original and reconstructed brain data—under the assumption that models trained on healthy data will fail to reconstruct pathological inputs accurately. However, well-trained autoencoders can generalise sufficiently to reconstruct anomalous data accurately, making reconstruction error an unreliable marker of abnormality (Bouman & Heskes, 2025).

Recognising these limitations, Lawry Aguila et al. (2022) introduced a latent-based metric that measures deviations directly in the latent space using a conditional variational autoencoder (cVAE), which captures uncertainty through probabilistic latent representations. By conditioning both encoder and decoder on covariates such as age and sex, this framework encourages the latent space to capture residual variation independent of known demographic factors—a strategy that aligns with broader principles of disentanglement learning in generative models (Pati & Lerch, 2021). This approach was later extended to handle multiple neuroimaging data by using generalised Product-of-Experts and Mixture-of-Experts VAE models (Lawry Aguila et al., 2023). More recently, Ijishakin et al. (2024) further advanced this field by applying diffusion autoencoder-based normative models and using latent space deviations as the primary method for deviation quantification. Briefly, these latent-based approaches quantify deviations by encoding the observed brain data into a joint latent space and then comparing each subject’s latent representation to the healthy controls using various distance metrics such as z-score (Lawry Aguila et al., 2022), Mahalanobis distance (Lawry Aguila et al., 2023), or cosine similarity (Ijishakin et al., 2024). These latent-based metrics capture overall deviation by accounting for correlations between latent dimensions and leveraging the shared information across modalities. However, because these metrics operate in the latent space, they do not directly indicate which features or brain regions are contributing to the observed deviation. To localise abnormalities, latent dimensions showing statistically significant group differences must be identified and then reconstructed into the original feature space, where the resulting outputs are interpreted as feature-wise deviations (Lawry Aguila et al., 2022). This process introduces additional challenges: selective reconstruction requires arbitrary thresholding and is sensitive to factors such as latent space dimensionality and cohort characteristics. Similarly to reconstruction-error-based deviations, well-trained VAEs may still accurately reconstruct anomalous data, which challenges the assumption that good reconstruction implies normal brain structure. Moreover, reconstructing only a subset of latent components while setting the remainder to zero risks biased or incomplete representations.

Autoencoder (AE)-based normative models represent a major shift towards more complex, non-linear modelling of brain data, leveraging deep learning’s capacity to handle multiple variables simultaneously. Nevertheless, these models present several conceptual and practical limitations in how they quantify and interpret deviations from normative patterns. First, existing AE-based approaches diverge fundamentally from the traditional normative modelling paradigm. Conventional normative models follow a clear two-stage process: they first establish a statistical norm or reference distribution based solely on covariates (e.g., age, sex) and then identify deviations by comparing an individual’s observed data against this covariate-specific norm (Marquand, Rezek, et al., 2016; Rutherford et al., 2022). In contrast, existing AE-based models generate brain measures through posterior sampling, in which the latent variables are inferred using both covariates and actual brain data before reconstruction. This dual-input approach prevents the construction of a pure covariate-based norm, making it difficult to determine whether identified deviations reflect true abnormalities or artefacts of the inference process. Second, reconstruction-based deviation metrics themselves are inherently limited. The reconstruction error, typically computed as an unsigned difference between the reconstructed and observed values, fails to capture the direction of deviation. In neuroimaging studies, this directionality is essential for biological interpretation, as it distinguishes between atrophy and hypertrophy or between hypo- and hyperintensities. Moreover, well-trained autoencoders reconstruct even abnormal data accurately (Bouman & Heskes, 2025), further weakening reconstruction error as a reliable indicator of abnormality. While latent-based extensions attempt to address this by assessing deviations in the latent space, they still require selective reconstruction to obtain feature-specific deviations. This process involves arbitrary thresholding decisions that may introduce bias, undermining the reliability of deviation measures. Finally, these models do not provide probabilistic predictions, which are crucial in normative modelling for understanding individual variations in brain structure and function across a population. Collectively, these conceptual and technical limitations constrain both the interpretability and reliability of conclusions drawn from such AE-based normative models.

In this study, we make two key contributions to the field of normative modelling. First, we propose an alternative inference approach for cVAE-based normative modelling that better aligns with traditional normative modelling principles and leverages the model’s generative capabilities. Instead of relying on a dual-input approach, our model generates brain measures directly from relevant covariates by sampling from the prior distribution in the latent space. Second, despite growing interest in deep learning for normative modelling, no studies have systematically compared these approaches against established statistical methods. This gap makes it difficult to assess whether the increased computational complexity of deep learning is justified by improved performance, and under what circumstances each approach might be preferred. We address this gap by providing the first comprehensive benchmarking of deep learning against traditional statistical normative models, systematically evaluating our approach against three well-established methods (GAMLSS, MFPR, and HBR) as well as the conventional posterior-sampling cVAE. To evaluate our model’s performance on neuroimaging data, we used 195 imaging-derived phenotypes, including morphometric features (cortical thickness, cortical volume, subcortical volume from FreeSurfer) and white matter hyperintensity (WMH) volumes caused by cerebral small vessel disease across different brain regions (see Supplementary Table S1 and Supplementary Fig. S1) as a test case. We modelled these features by incorporating relevant covariates, including age, sex, total intracranial volume, and several vascular risk factors. Age and sex are fundamental factors in brain morphology’s variations (Coffey et al., 1998; Zhao et al., 2019), while total intracranial volume accounts for head size. The modifiable vascular risk factors we included (diabetes, hypercholesterolemia, obesity, and smoking) are key contributors to cerebrovascular health and are associated with the manifestation of WMH on brain scans (Williamson et al., 2018).

We assessed our approach through four analyses. First, we performed a covariate sensitivity analysis comparing our prior inference method with the conventional posterior-sampling approach used in existing AE-based models (Pinaya et al., 2019, 2021). This analysis reveals how predicted brain measures from these two inference strategies respond to covariate perturbations, thereby exposing potential biases. Second, we compared the predictive performance of our cVAE-based normative model against established normative modelling algorithms (GAMLSS, MFPR, and HBR) as well as the conventional posterior-sampling cVAE using a hold-out dataset. Third, we conducted an Expected Calibration Error (ECE) analysis to validate the quality of our model’s uncertainty estimates. Finally, we performed a comprehensive deviation analysis comprising two components: (1) validation of our model’s ability to generate covariate-independent deviation scores on the hold-out dataset and (2) clinical validation examining the relationship between individual deviations and hypertension levels, which have been shown to correlate with increased white matter burden (de Leeuw et al., 2002; Du et al., 2024) and cortical atrophy (Beauchet et al., 2013). Together, these analyses demonstrate the feasibility and validity of the proposed prior-sampling cVAE framework, which overcomes the limitations of posterior-sampling cVAEs and provides a systematic comparison with established statistical normative models.

## 2 Methods and Materials

### 2.1 Participants

**Fig. 1.**
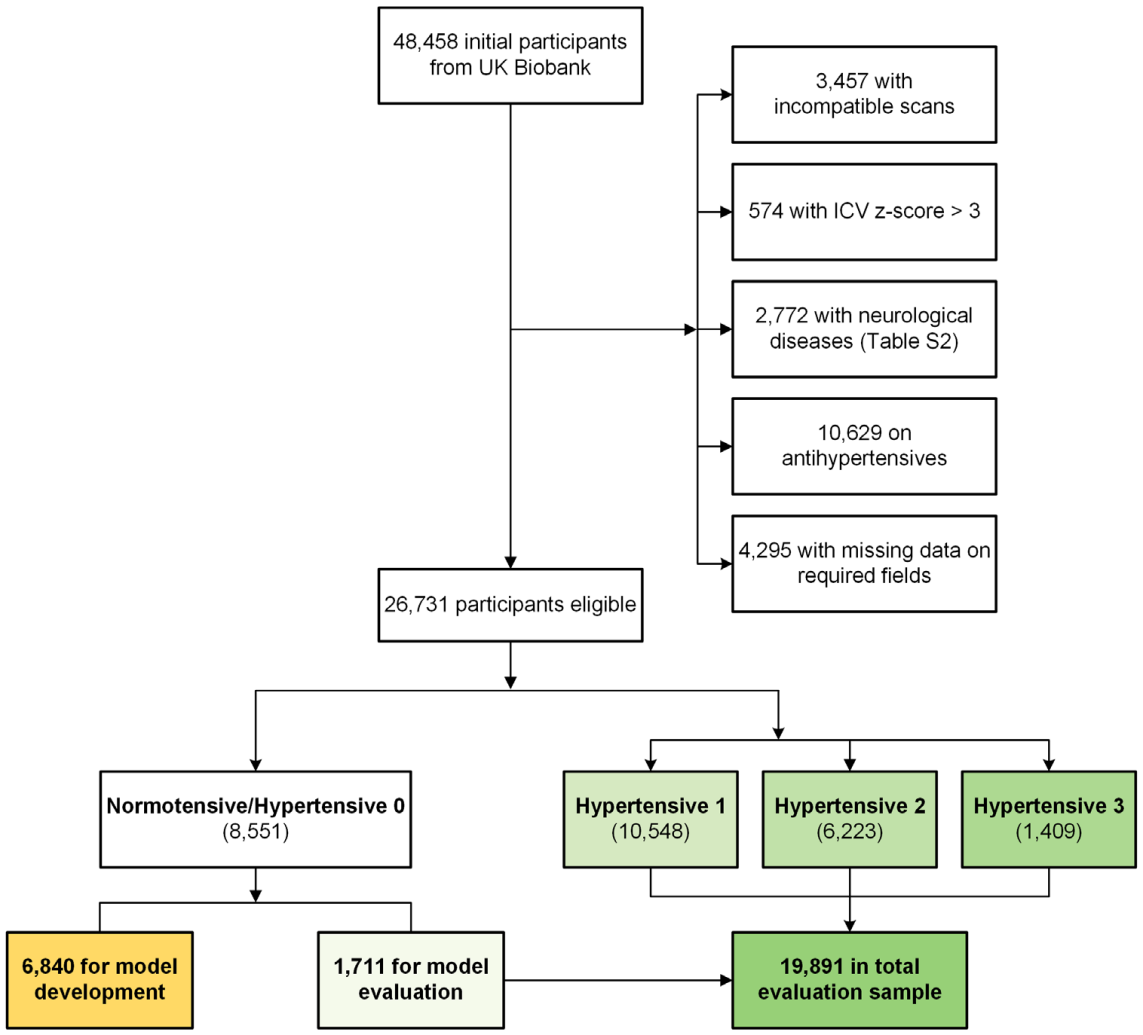
Flowchart of participant selection. T1w = T1-weighted; T2w-FLAIR = T2-weighted-Fluid-Attenuated Inversion Recovery; ICV = intracranial volume.

The data for this study were obtained from UK Biobank (project #98013). UK Biobank is a large-scale cohort study that includes comprehensive health information from over half a million UK participants (Sudlow et al., 2015). A flowchart detailing the selection of participants for the current study is presented in Figure 1. Initially, 48,458 participants with both T1-weighted (T1w) and T2-weighted-Fluid-Attenuated Inversion Recovery (T2w-FLAIR) scans were considered. Several exclusion criteria were applied as follows: participants with incompatible or unusable T1w/FLAIR scans (*n* = 3,457), those with intracranial volume z-scores greater than 3 (*n* = 574), individuals diagnosed with specific neurological disorders outlined in Supplementary Table S2 (*n* = 2,772), and those with incomplete data on required variables to determine vascular risk factors, for example, diabetes diagnosis by doctor (*n* = 4,295). Additionally, to eliminate potential confounding effects from antihypertensive use on the study’s outcomes, 10,629 participants who were on antihypertensive medication were excluded. After these exclusions, a total of 26,731 participants remained for analysis.

For clinical relevance assessment, eligible participants were classified into four groups based on blood pressure measurements, following the Global Hypertension Practice guidelines by the International Society of Hypertension (Unger et al., 2020). The normotensive group (Hypertension 0) comprised 8,551 participants with systolic blood pressure (SBP) <130 mmHg and diastolic blood pressure (DBP) <85 mmHg. The hypertensive groups were defined as follows: Hypertensive 1 (*n* = 10,548) with SBP between 130 and 139 mmHg, or DBP between 85 and 89 mmHg; Hypertensive 2 (*n* = 6,223) with SBP between 140 and 159 mmHg, or DBP between 90 and 99 mmHg; and Hypertensive 3 (*n* = 1,409) with SBP ≥160 mmHg or DBP ≥100 mmHg.

For model development, 80% (*n* = 6,840) of the normotensive group was randomly selected to form the training dataset. The remaining 20% (*n* = 1,711) of the normotensive group served as the hold-out dataset, which was used to assess the model’s performance. This hold-out dataset was then combined with all hypertensive participants to create an evaluation sample of 19,891 participants, used to test the model’s clinical relevance across different levels of hypertension severity.

### 2.2 Vascular risk factors

Several vascular risk factors other than hypertension were considered as part of the covariates. These factors, treated as binary covariates, were diabetes, hypercholesterolemia, obesity, and smoking. Participants with diabetes were identified based on a doctor’s diagnosis and the use of antidiabetic medication. Hypercholesterolemia was determined through participants’ medication records, indicating treatment for elevated cholesterol levels. Obesity was defined as having a body mass index (BMI) of 30 or higher. Smoking status was identified as current or former smoker based on participants’ self-reported smoking history. Controlling for these factors allowed us to better assess the relationship between WMH volumes and hypertension, minimising the influence of other vascular conditions on the results.

### 2.3 MRI acquisition and data preprocessing

T1w and FLAIR scans were acquired from three UK imaging centres (Cheadle Greater Manchester, Newcastle and Reading), all using a 3T Siemens Skyra scanner with a standard Siemens 32-channel head coil and uniform imaging parameters (Miller et al., 2016). Full details of the imaging protocols are available in the online UK Biobank brain imaging documentation (Smith et al., 2024).

Structural brain measures, including whole-brain intracranial volumes and regional morphometric measures, were derived from T1w images using FreeSurfer (Fischl, 2012), as part of the UK Biobank’s standard image processing pipeline (Alfaro-Almagro et al., 2018). Cortical measures were parcellated using the Desikan–Killiany–Tourville (DKT) atlas (see Supplementary Fig. S1). These included regional cortical thickness for 31 regions per hemisphere (62 features total), and cortical volumes comprising 31 regions per hemisphere plus 3 global measures (total grey matter volume, left cortical volume, and right cortical volume; 65 features total). Subcortical measures were obtained through automated subcortical segmentation, yielding volumes for 38 subcortical structures. Whole-brain intracranial volume, essential for adjusting for head size, was also included as a covariate in the development of normative models.

White matter hyperintensity (WMH) volumes were computed in native space using the UBO (Unidentified Bright Object) Detector, a cluster-based, fully automated toolbox that segments and calculates WMH volumes from T1w and FLAIR images (Jiang et al., 2018). Three parcellation schemes were applied to extract the volumes for different regions of interest: (1) whole-brain, periventricular, and deep WMH; (2) lobar WMH volumes, with the brain divided into 11 lobar regions; and (3) WMH volumes based on arterial territories, with parcellation according to 16 blood supply areas (Wen & Sachdev, 2004). The full list of brain regions and their corresponding abbreviations is presented in Supplementary Table S1. To ensure data integrity, all segmented WMH clusters underwent visual quality-control procedures (see Supplementary Methods S1 for further details).

All brain measures were z-score standardised to normalise the data for subsequent analyses. For WMH volumes, which tend to be highly skewed, log transformation was applied prior to z-score standardisation. The normalised brain measures served as inputs for the development and evaluation of all models, resulting in a total of 195 features comprising 62 cortical thickness measures, 65 cortical volume measures, 38 subcortical volume measures, and 30 WMH measures across various brain regions.

### 2.4 cVAE-based model development

#### 2.4.1 Model architecture

The model employed in this study is a conditional Variational Autoencoder (cVAE), building on the auto-encoding variational Bayes algorithm introduced by Kingma and Welling (2013). Unlike a standard Autoencoder (AE), which deterministically maps data to a latent space and reconstructs it, a VAE introduces probabilistic modelling by learning a distribution over the latent space, enabling generative capabilities. The cVAE architecture consists of two main components: an encoder and a decoder. The encoder maps input data to a lower-dimensional latent space, while the decoder reconstructs the original data from this latent representation conditioned on covariates. To determine the optimal model architecture, specifically the dimensionality of the latent and hidden layers, we utilised the Optuna framework (Akiba et al., 2019) with fivefold cross-validation. Optuna is a Bayesian optimisation framework that efficiently searches the hyperparameter space by learning from previous trials, employing a tree-structured Parzen estimator (Watanabe, 2023). Through a total of 100 Optuna trials, the tuning process identified an optimal architecture with a 64-dimensional latent space and a single hidden layer containing 512 units. Further details regarding this hyperparameter optimisation process are provided in Supplementary Methods S2.

**Fig. 2.**
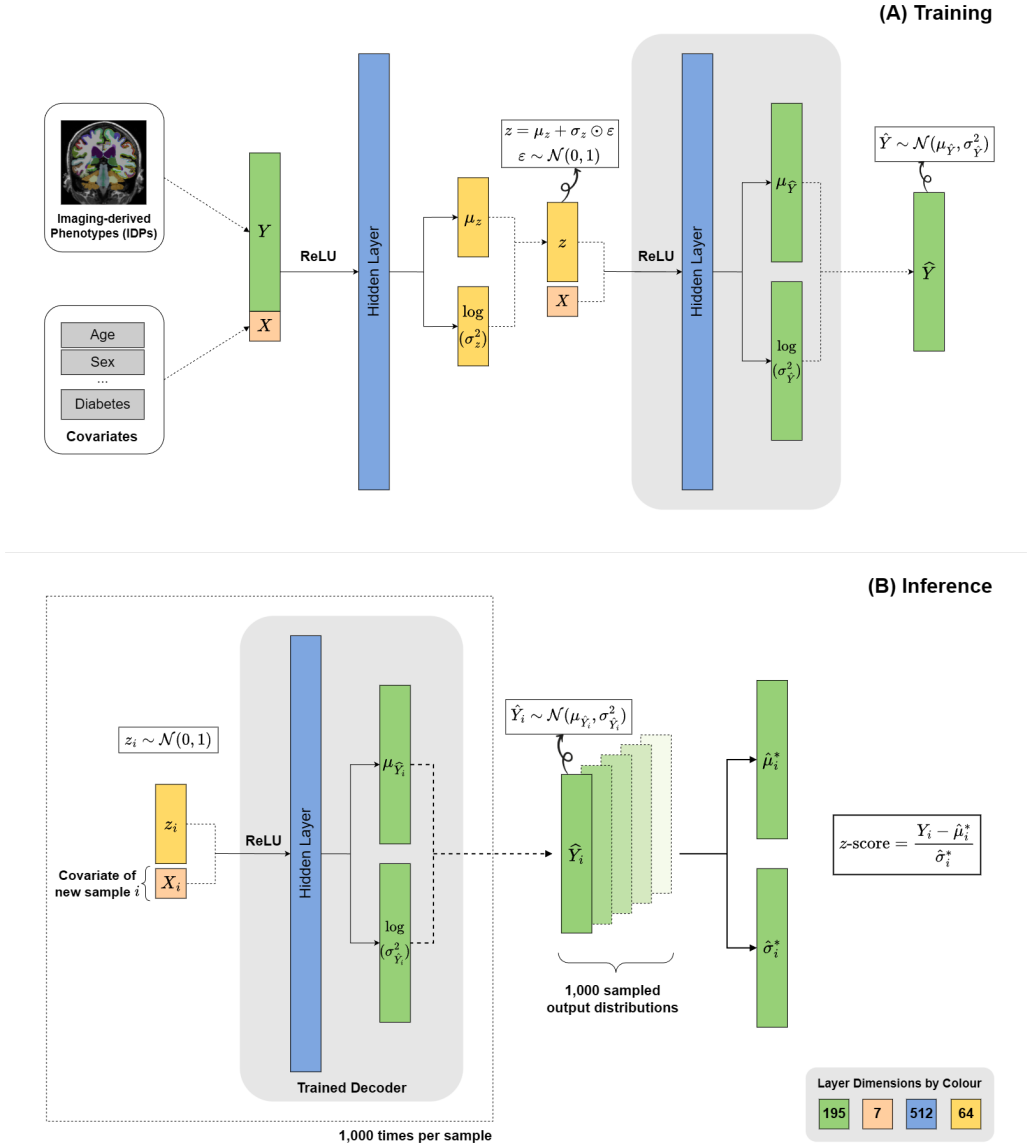
Model architecture and inference framework. This diagram illustrates a conditional Variational Autoencoder (cVAE) architecture for modelling imaging-derived features across brain regions. (A) The top panel depicts the model architecture and training phase, showing how 195 brain measures and 7 covariates of the training dataset are processed through the encoder, latent space, and decoder. (B) The bottom panel demonstrates the inference process, where the trained decoder is used to generate new predictions. This process is repeated 1,000 times to synthesise 1,000 output distributions, from which summary statistics (${\hat{\mu }}^{*}$ and ${\hat{\sigma }}^{*}$) are computed per sample. Layers represented in the same colour indicate identical dimensions. ReLU = Rectified Linear Unit. ⊙ denotes element-wise multiplication.

In this study, the cVAE framework is designed to predict brain morphometric measures and WMH volumes across different regions while accounting for covariates, including age, sex, total intracranial volume, and vascular risk factors (diabetes, hypercholesterolemia, obesity, and smoking). To ensure numerical stability, age and total intracranial volume were normalised to standard normal distribution before being fed into the model. The comprehensive cVAE framework is illustrated in Figure 2, highlighting both the training and inference phases. During training, the encoder receives two inputs: (1) the brain measures (denoted as Y) comprising 195 features (62 cortical thickness measures, 65 cortical volume measures, 38 subcortical volume measures, and 30 WMH measures) and (2) a set of 7 covariates (denoted as X). These inputs pass through a 512-unit hidden layer with rectified linear unit (ReLU) activation, allowing the model to capture non-linear relationships between the brain features and covariates.

The encoder outputs two key latent variables: the mean ($\mu_{z}$) and the log-variance ($\log(\sigma_{z}^{2})$ ), both represented as 64-dimensional vectors. The latent variables define a multivariate Gaussian distribution from which a latent variable z is sampled. As proposed by Kingma and Welling (2013), the reparameterisation trick is applied to enable backpropagation through stochastic layers. Specifically, z is calculated as

$$z\;=\;\mu_{z}+\sigma_{z}⊙\varepsilon .$$

Here, ε~N(0,1) is a random noise variable sampled from a standard normal distribution and ⊙ denotes element-wise multiplication. This technique allows the sampling process to be expressed as a deterministic operation, making it possible to compute gradients and update model parameters during training.

Subsequently, the decoder reconstructs brain measures by processing a 71-length input (latent vector z concatenated with covariates) through a 512-unit hidden layer with ReLU activation. The decoder produces two outputs that define a probabilistic distribution over the reconstructed brain measures. The first output is the mean vector ($\mu_{\hat{Y}}$ ), obtained by transforming the 512-dimensional hidden representation through a linear layer to produce a 195-dimensional vector. The second output is the log-variance ($\log(\sigma_{\hat{Y}}^{2})$ ), obtained through a separate linear layer that also transforms the 512-dimensional hidden representation to produce a 195-dimensional vector. Together, these components define the final output, which is a multivariate normal distribution $\hat{Y}\sim \;N(\mu_{\hat{Y}},\;\sigma_{\hat{Y}}^{2})$ . From this distribution, multiple predictions of WMH volumes can be sampled. The rationale behind outputting a distribution rather than a deterministic value is twofold. First, brain structure measurements vary significantly between individuals (Habes et al., 2016). Modelling the output as a distribution thus better captures this inherent biological variability, which a single point estimate might overlook. Second, outputting a distribution is essential for training under the variational autoencoder framework, which relies on optimising a differentiable lower bound on the marginal data likelihood (Kingma & Welling, 2013). Thus, the probabilistic decoder is fundamental to the variational objective, which is defined in terms of a likelihood over reconstructed data.

#### 2.4.2 Training phase

The cVAE is trained by minimising a loss function that combines two terms: the Kullback–Leibler (KL) divergence (Kullback & Leibler, 1951) and the reconstruction loss. The reconstruction loss measures how well the model can reconstruct the original brain measures from the latent space, while the KL divergence serves as a regularisation term, encouraging the latent space to remain close to a predefined prior distribution (N(0,1)). The overall loss function (ℒ) of the cVAE is defined as

$$ℒ=\underset{\text{Regularisation (KL divergence)}}{\underset{︸}{-\text{KL}(q_{\phi }(z\;\;\text{|}\;Y,X)∥p(z))}}\;\;+\;\;\underset{\text{Reconstruction Loss}}{\underset{︸}{{\text{E}}_{q_{\phi }\left(z\;\text{|}\;Y,X\right)}[\log p_{\theta }(Y\;\text{|}\;\;z,X)]}}$$.

In this equation, the first term is the KL divergence, which measures the difference between the learned posterior distribution $q_{\phi }\text{(}\;z\;\text{|}\;Y,X)$ and the Gaussian prior distribution p(z) . The second term represents the expected log-likelihood of generating the input Y given the latent representation z and covariates X. The encoder and decoder parameters are denoted as ϕ and θ, respectively.

For our implementation, we trained the model using the Adam optimiser, with hyperparameter tuning conducted via the Optuna framework (Akiba et al., 2019) using fivefold cross-validation on the training dataset. For each fold, 20 Optuna trials with adaptive pruning (median pruner with 5 start-up trials and 20 warm-up steps) were executed to systematically explore the hyperparameter space, including latent space dimensions, hidden layer configurations, learning rates, and batch sizes. This resulted in a total of 100 trials (20 trials × 5 folds) to identify the optimal hyperparameter combination that minimised the mean cross-validation loss.

After identifying the best hyperparameter configuration from cross-validation, we retrained the final model on the entire training dataset (combining all training and validation folds). For this final training, we reserved 10% of the data as a validation set to monitor training progress. To prevent overfitting, we applied early stopping with a patience of 20 epochs, monitoring the validation loss. The model with the lowest validation loss was selected as the final trained model for subsequent inference. Further details on the analysis of training and validation loss are provided in Supplementary Methods S3.

#### 2.4.3 Inference phase

The inference phase of our framework introduces a novel approach for generating brain features using a cVAE in normative modelling studies. Unlike conventional AE-based models that use both covariates and actual brain measures for prediction (Lawry Aguila et al., 2022, 2023; Pinaya et al., 2019, 2021), our inference strategy relies directly on relevant covariates. This design better aligns with traditional statistical normative models, which generate reference distributions based purely on the covariates rather than being influenced by the observed values.

Our inference process consists of two key steps. First, for each participant i with covariates $X_{i}$ in the evaluation dataset, we sampled a 64-dimensional latent vector *$z_{i}$* from the standard normal distribution N(0,1), which serves as the prior for the latent space. This vector was concatenated with $X_{i}$ and passed through the trained cVAE decoder to output a predicted distribution ${\hat{Y}}_{i}\sim N\left(\mu_{{\hat{Y}}_{i}},\;\sigma_{{\hat{Y}}_{i}}^{2}\right)$, where $\mu_{{\hat{Y}}_{i}}$ is the mean and $\sigma_{{\hat{Y}}_{i}}^{2}$ is the variance derived from the decoder’s output layers.

Second, to capture the inherent variability in the data, this entire process—from sampling a new latent vector $z_{i}$ to obtaining the distribution parameters—was repeated 1,000 times per participant. This number was chosen based on preliminary experiments and also exceeds the minimum of 399 recommended by Davidson and MacKinnon (2000) for estimating a 95% confidence interval. This iterative sampling yields 1,000 sets of distribution parameters $\left\{\mu_{{\hat{Y}}_{i}}^{\left(k\right)},\sigma_{{\hat{Y}}_{i}}^{2\left(k\right)}\right\}$, for k=1,…,1000 , each representing the decoder’s predicted distribution given different latent samples. From these 1,000 parameter sets, the predicted mean for participant i was calculated as

$${\hat{\mu }}_{i}^{*}=\frac{1}{1000}\sum_{k=1}^{1000}\mu_{{\hat{Y}}_{i}}^{\left(k\right)}.$$

Following the law of total variance, the predicted variance was decomposed into within-iteration variance and between-iteration variance:

$${\hat{\sigma }}_{i}^{2*}=\underset{\text{Within-iteration variance}}{\underset{︸}{\frac{1}{1000}\sum_{k=1}^{1000}\sigma_{{\hat{Y}}_{i}}^{2(k)}}}+\underset{\text{Between-iteration variance}}{\underset{︸}{\frac{1}{1000}\sum_{k=1}^{1000}{\left(\mu_{{\hat{Y}}_{i}}^{\left(k\right)}-{\hat{\mu }}_{i}^{*}\right)}^{2}}}.$$

This decomposition aligns with the total predictive variance formulation adopted in the PCNtoolkit for HBR (de Boer et al., 2024), which jointly accounts for aleatoric and epistemic sources of uncertainty. Together, the predicted mean (${\hat{\mu }}_{i}^{*}$) and standard deviation (${\hat{\sigma }}_{i}^{*}$) constitute the final predictions for each participant i, representing the expected healthy brain profiles given their covariate characteristics.

### 2.5 Comparable normative models

We compared our proposed cVAE framework with four alternative approaches: three well-established statistical models and the conventional posterior-sampling cVAE method. The statistical models included Generalised Additive Models for Location, Scale, and Shape (GAMLSS), Multivariate Fractional Polynomial Regression (MFPR), and Hierarchical Bayesian Regression (HBR). The posterior-sampling cVAE represents the conventional approach employed in existing autoencoder-based normative models (Pinaya et al., 2019, 2021), using both covariates and observed brain measures for prediction. To clearly distinguish between these two inference strategies, we hereafter refer to our proposed approach as prior-cVAE and the conventional approach as posterior-cVAE. While Gaussian Process Regression (GPR) was one of the earliest normative modelling approaches, we did not include it as part of the comparable models due to its significant limitations in computational efficiency and scalability. Instead, HBR serves as a more computationally tractable alternative that was specifically developed to address GPR’s limitations while maintaining similar modelling capabilities (de Boer et al., 2024).

For GAMLSS, we implemented a Gaussian family distribution model with P-splines for age, enabling automatic selection of smoothing parameters. Additionally, an automated model selection process using the Bayesian Information Criterion (BIC) was used to determine the optimal distribution family and smoothing parameters for each feature. Both location and scale parameters were modelled as functions of covariates. For MFPR, we employed a two-stage modelling approach to obtain both conditional mean and variance estimates. First, we used the mfp package in R with its closed test procedure to systematically select the most appropriate fractional polynomial functions from the standard set of powers {-2, -1, -0.5, 0, 0.5, 1, 2, 3} for conditional mean modelling. Second, since MFPR does not directly estimate variance, we computed the root mean squared error (RMSE) of the residuals from the first-stage model as an estimate of the residual standard deviation, aligning with the approach described in the CentileBrain study (Ge et al., 2024). The HBR model, implemented using the PCNtoolkit package (Kia et al., 2020), handles non-linearity through B-spline basis functions with adaptive regularisation via hyperpriors on smoothness parameters. It estimates the total predictive variance by integrating both aleatoric and epistemic sources of uncertainty through Markov chain Monte Carlo sampling (de Boer et al., 2024). All statistical models were trained using fivefold cross-validation to ensure robust performance estimation and reduce the risk of overfitting, with each brain region modelled independently.

In the posterior-cVAE approach, we used the same trained cVAE model as our proposed prior-cVAE method, differing only in inference strategy. As described in Section 2.4.3, our prior-sampling approach samples the latent vector $z_{i}$ from the prior distribution N(0,1) and uses it together with the covariates $X_{i}$ to estimate brain features. In contrast, the posterior-sampling approach maps both the covariates $X_{i}$ and observed measures $Y_{i}$ to an approximate posterior distribution $q_{\phi }(z_{i}Y_{i},X_{i})$ in the latent space, from which $z_{i}$ is sampled. These latent vectors are then passed through the decoder along with the covariates to produce parameters for a multivariate normal distribution. For consistency, probabilistic predictions are produced using the same sampling protocol. This process is illustrated in detail in Supplementary Figure S2.

### 2.6 Covariate sensitivity analysis

To evaluate the reliability of our proposed inference approach, we conducted a covariate sensitivity analysis focusing on age, a variable with well-established correlations with cortical atrophy (Fujita et al., 2023) and WMH volumes (Garnier-Crussard et al., 2020). This analysis was designed to assess how model predictions respond to controlled perturbations in age, thereby determining the robustness and sensitivity of each inference strategy when subjected to systematic changes.

The covariate sensitivity analysis was conducted in several steps. First, we computed the standard deviation for age from the training dataset. For each participant in the hold-out dataset, we then created a perturbed version by adding this training-derived standard deviation to their age while keeping all other covariates constant. Subsequently, we generated predicted brain measures for both the original and perturbed datasets using the prior and posterior sampling approaches described in Sections 2.4.3 and 2.5. The sensitivity analysis was then performed on both regional and global levels.

At the regional level, we compared the median predictions between original and perturbed datasets for each brain region and sampling method separately. The shift in predictions was quantified as the difference between perturbed and original medians. For the global analysis, we computed the perturbation effects as the absolute differences between the original and perturbed predictions for each inference method across all brain regions. The Wilcoxon signed-rank test was then conducted on these perturbation effects to assess whether one sampling method exhibited statistically greater sensitivity to age perturbation than the other. A significant result in this global analysis would indicate fundamental differences in how the two sampling approaches respond to covariate perturbations, potentially revealing inherent biases in either approach.

### 2.7 Deviation score computation

To quantify the deviation of observed values from model predictions, a standardised deviation score was computed. Specifically, the deviation score $z_{ij}$ for participant i and feature j is defined as

$$z_{ij}=\;\frac{Y_{ij}-{\hat{\mu }}_{ij}^{*}}{{\hat{\sigma }}_{ij}^{*}},$$

where $Y_{ij}$ is the observed value, ${\hat{\mu }}_{ij}^{*}$ denotes the predicted mean, and ${\hat{\sigma }}_{ij}^{*}$ represents the predicted standard deviation for participant i and feature j.

### 2.8 Model performance metrics

To comprehensively evaluate the performance of our prior-cVAE approach against other normative models (posterior-cVAE, GAMLSS, MFPR and HBR), we selected a set of metrics that capture different aspects of model performance. These metrics include (1) *Median Absolute Error*, (2) *Root Mean Squared Error*, (3) *Spearman’s Correlation Coefficient*, and (4) *Explained Variance*.

Median Absolute Error was used to measure the typical prediction error across all participants by calculating the median of the absolute differences between observed and predicted mean values. Unlike Mean Absolute Error, it is less influenced by outliers, making it particularly informative for data with non-Gaussian characteristics such as WMH volumes. Root Mean Squared Error (RMSE) quantifies the overall prediction accuracy by measuring the square root of the average squared differences between observed and predicted values. RMSE is sensitive to larger errors and provides a comprehensive assessment of model performance across the entire distribution. Spearman’s Correlation Coefficient was employed to assess the monotonic relationship between observed and predicted values without assuming linearity. Meanwhile, Explained Variance, a common metric in normative modelling studies (Ge et al., 2024; Rutherford et al., 2022), indicates the proportion of variability in observed values that each model can capture. The mathematical formulation and interpretation of each metric are provided in Table 1.

**Table 1.** Performance metrics.

Name	Formula	Interpretation
Median Absolute Error (MedianAE)	$MedianAE_{j}=median\left(\sum_{i=1}^{N}\left|Y_{ij}-{\hat{\mu }}_{ij}^{*}\right|\right),$ where N is the number of participants, $Y_{ij}$ is the observed value, and ${\hat{\mu }}_{ij}^{*}$ is the predicted mean for participant i and feature j.	Lower values indicate better model performance, with zero representing a perfect prediction.
Root Mean Squared Error (RMSE)	$RMSE_{j}=\sqrt{\frac{1}{N}\sum_{i=1}^{N}{\left(Y_{ij}-{\hat{\mu }}_{ij}^{*}\right)}^{2}}$ where N is the number of participants, $Y_{ij}$ is the observed value, and ${\hat{\mu }}_{ij}^{*}$ is the predicted mean for participant i and feature j.	Lower values indicate better model performance, with zero representing a perfect prediction.
Spearman’s Correlation Coefficient (ρ)	$\rho_{j}=1-\frac{6\sum_{i=1}^{N}d_{ij}^{2}}{N(N^{2}-1)},$ where N is the number of participants and $d_{ij}$ is the difference between the ranks of $Y_{ij}$ , the observed value and ${\hat{\mu }}_{ij}^{*}$, the predicted mean for participant i and feature j.	Spearman’s ρ ranges between -1 and 1. A value closer to 1 indicates better model performance, suggesting strong positive monotonic relationship where predicted values increase with observed values. A value of -1 indicates a perfect negative relationship, while 0 indicates no monotonic relationship.
Explained Variance (EV)	$EV_{j}=1-\frac{\sum_{i=1}^{N}{\left(Y_{ij}-{\hat{\mu }}_{ij}^{*}\right)}^{2}}{\sum_{i=1}^{N}{\left(Y_{ij}-{\bar{Y}}_{j}\right)}^{2}},$ where N is the number of participants, $Y_{ij}$ is the observed value, ${\hat{\mu }}_{ij}^{*}$ is the predicted mean for participant i and feature j, and ${\bar{Y}}_{j}$ is the mean of the observed values for feature j across all participants.	Explained variance ranges from (-∞, 1], with 1 indicating that the model accounts for 100% of the variance in the observed data. A value of 0 means the model’s predictions are no better than simply predicting the mean value, while negative values indicate worse performance than predicting the mean.

To assess whether the performance differences between our proposed approach (prior-cVAE) and each comparison model (posterior-cVAE, GAMLSS, MFPR, and HBR) were statistically significant, median-based permutation tests were conducted. This non-parametric approach evaluates whether observed differences in performance metrics could have occurred by chance. Briefly, for each feature, we first calculated the observed median difference, defined as the difference between the median performance score (e.g., RMSE) of the prior-cVAE model and that of the comparison model. Next, a null distribution was constructed by pooling all feature-wise scores from both models and randomly partitioning them into two groups of their original sizes. The median difference between the two reshuffled groups was then computed, and this procedure was repeated 10,000 times to form a distribution of median differences expected under the null hypothesis of no true difference between models. The empirical *p*-value was calculated as the proportion of shuffled differences whose absolute values were greater than or equal to that of the observed difference. This process was repeated independently for each model comparison, ensuring a robust and distribution-free assessment of statistical significance in non-Gaussian data. To account for multiple comparisons across the numerous brain regions assessed, we applied False Discovery Rate (FDR) correction to the resulting *p*-values.

### 2.9 Expected calibration error analysis

To assess the quality of uncertainty estimates produced by each model, we computed the Expected Calibration Error (ECE) for each brain measure using the hold-out dataset. While the performance metrics primarily assess the accuracy of point predictions, ECE specifically determines how well the full probability distributions predicted by a model correspond to the observed outcomes. In the context of normative modelling, well-calibrated uncertainty estimates are crucial for computing reliable deviation scores and accurately identifying abnormalities.

The ECE quantifies the discrepancy between a model’s predicted probability distribution and the empirical distribution of observed values. For a normative modelling framework that outputs a predicted mean $\mu_{i}$and standard deviation $\sigma_{i}$ for each sample i and feature j, we can construct two-sided confidence intervals at various confidence intervals α. For a given confidence level α, we define the confidence interval as

$$CI_{\alpha }\left(i,j\right)=\left[\mu_{ij}-z_{\frac{\left(1+\alpha \right)}{2}}\sigma_{ij},\;\mu_{ij}+z_{\frac{\left(1+\alpha \right)}{2}}\sigma_{ij}\right].$$

Here, $z_{\frac{\left(1+\alpha \right)}{2}}$ is the $\left(\frac{1+\alpha }{2}\right)$-quantile of the standard normal distribution. For a perfectly calibrated model, the proportion of true values falling within these intervals should equal to the confidence level α.

To compute the ECE, we evaluate this property across a range of confidence levels $\left\{\alpha_{1},\alpha_{2},...,\alpha_{m}\right\}$ . For each confidence level and feature, we calculate the observed coverage (the fraction of true values that fall within the predicted confidence intervals) and compare it with the expected coverage. The ECE for feature j is then defined as the average absolute deviation between observed and expected coverage across all confidence levels. In our implementation, we used confidence levels ranging from 0.05 to 0.95 in increments of 0.1, resulting in 10 quantile bins for computing the ECE.

### 2.10 Deviation analysis

#### 2.10.1 Covariate independence analysis

On the hold-out dataset, z-scores were computed from all five modelling approaches (prior-cVAE, posterior-cVAE, GAMLSS, MFPR, and HBR) to assess how individual data points deviate from the normative predictions. We examined the relationships between all covariates (age, sex, total intracranial volume, diabetes, hypercholesterolaemia, obesity, and smoking) and the obtained z-scores across all 195 imaging-derived phenotypes to evaluate each model’s ability to generate predictions that were independent of covariates. This analysis was essential in ensuring that the deviation scores reflected clinically relevant abnormalities, rather than being confounded by the covariates used in model training. Spearman’s correlation coefficients were calculated between each covariate and z-scores for each brain measure, with statistical significance assessed after False Discovery Rate (FDR) correction for multiple comparisons.

#### 2.10.2 Clinical application

In the entire evaluation dataset, we explored the relationship between z-scores derived from all five models (prior-cVAE, posterior-cVAE, GAMLSS, MFPR, and HBR) and different hypertension levels (graded from 0 to 3) using Spearman’s correlation test. This analysis was conducted across all 195 imaging-derived phenotypes to assess each model’s sensitivity to clinically relevant abnormalities associated with hypertension severity. Reported *p*-values were corrected for multiple comparisons using the False Discovery Rate (FDR) method. Additionally, the percentage of extreme deviations (z-score > 2.58 and z-score < -2.58) was calculated and compared across the different hypertension levels to understand how hypertension influences brain structure abnormalities. The threshold of ±2.58 corresponds to the 99th percentile of a standard normal distribution, representing statistically significant deviations that occur in approximately 1% of a healthy population.

### 2.11 Ethics statement

This study used data from UK Biobank under its Research Tissue Bank (RTB) approval, granted by the Northwest Multi-centre Research Ethics Committee (REC reference: 16/NW/0274). All UK Biobank participants provided written informed consent for their data to be used in health-related research. Data access and analysis procedures complied with UK Biobank’s data protection policies and governance framework.

## 3 Results

### 3.1 Sample characteristics and distribution of brain measures

Sample characteristics, including demographics and vascular risk factors of training and evaluation data, are summarised in Table 2. The training sample consisted of 6,840 normotensive individuals (referred to as hypertension level 0 in Table 2), with a mean age of 60.76 ± 7.20 years, of whom 30.8% were male. The evaluation sample included 19,891 participants, further subdivided into 1,711 normotensive (mean age 61.24 ± 7.33 years, 31.0% male) and 18,180 hypertensive individuals (mean age 64.69 ± 7.49 years, 48.6% male). Hypertension levels were classified into three groups (1–3), with 58.0% of hypertensive individuals falling into level 1, 34.2% into level 2, and 7.8% into level 3. Hypertensive participants exhibited higher rates of vascular risk factors, including diabetes, hypercholesterolaemia, obesity, and smoking, compared with normotensive participants.

**Table 2.** Characteristics of samples (*n* = 26,731).

	Training sample		Evaluation sample		
	Normotensive (*n* = 6,840)	Hypertensive (*n* = 0)	Total (*n* = 19,891)	Normotensive (*n* = 1,711)	Hypertensive (*n* = 18,180)
Demographics					
Male, number (%)	2,104 (30.8%)	-	9,368 (47.1%)	530 (31.0%)	8,838 (48.6%)
Chronological age, years, mean ± SD	60.76 ± 7.20	-	64.39 ±7.54	61.24 ± 7.33	64.69 ± 7.49
Chronological age, range [min, max]	[45.13, 81.68]		[46.03, 83.39]	[46.03, 81.36]	[46.07, 83.39]
Risk factors					
Diabetes, number (%)	120 (1.8%)	-	552 (2.8%)	30 (1.8%)	522 (2.9%)
Hypercholesterolaemia, number (%)	508 (7.4%)	-	2,793 (14.0%)	148 (8.6%)	2,645 (14.5%)
Obesity, number (%)	578 (8.5%)	-	3,198 (16.1%)	139 (8.1%)	3,059 (16.8%)
Smoking, number (%)	2,309 (33.8%)	-	7,100 (35.7%)	587 (34.3%)	6,513 (35.8%)
Hypertension level, number (%)					
- Level = 0	6,840 (100.0%)	-	1,711 (8.6%)	1,711 (100.0%)	-
- Level = 1	-	-	10,548 (53.0%)	-	10,548 (58.0%)
- Level = 2	-	-	6,223 (31.3%)	-	6,223 (34.2%)
- Level = 3	-	-	1,409 (7.1%)	-	1,409 (7.8%)

SD = standard deviation.

Supplementary Table S3 provides descriptive statistics of the 195 imaging-derived phenotypes across the training and evaluation samples, comprising morphometric measures (cortical thickness, cortical volume, and subcortical volume) and WMH volumes. In the evaluation dataset, cortical measures showed hypertension-related reductions, with cortical thickness displaying subtle but consistent decreases across multiple regions (e.g., bilateral insula reductions of approximately 0.04–0.05 mm from normotensive to Hypertensive Level 3). More pronounced hypertension-related atrophy was observed in cortical volume measures, particularly in frontal and temporal regions. For instance, left superior frontal volume decreased by approximately 2% (from 25,467 ± 2,849 mm³ to 24,948 ± 2,924 mm³), while left middle temporal volume showed a reduction of approximately 3% (from 14,101 ± 1,938 mm³ to 13,669 ± 1,893 mm³). Subcortical structures demonstrated mixed patterns, with notable ventricular expansion across hypertension levels (e.g., left lateral ventricle increased by approximately 28%, from 11,690 ± 6,606 mm³ to 14,917 ± 8,055 mm³), while other structures such as the hippocampus showed modest volume reductions of approximately 2.5% (from 3,955 ± 399 mm³ to 3,858 ± 405 mm³).

WMH volumes showed clear hypertension-related increases across all measured regions. In the evaluation dataset, whole-brain WMH volumes increased from 1,736.62 ± 2,500.84 mm³ in normotensive participants to 2,433.40 ± 3,315.97 mm³ in Hypertensive Level 1, eventually reaching 3,933.86 ± 5,122.58 mm³ in Hypertensive Level 3. This pattern varied dramatically across anatomical regions. Among lobar areas, frontal regions exhibited the highest WMH burden in normotensive participants (left: 86.12 ± 306.76 mm³, right: 91.36 ± 284.00 mm³), followed by parietal lobes (left: 82.78 ± 275.56 mm³, right: 81.78 ± 252.76 mm³). At the arterial territory level, middle artery regions demonstrated the greatest burden (middle artery hemisphere—left: 242.81 ± 667.33 mm³, right: 267.17 ± 623.75 mm³).

Certain regions remained largely unaffected regardless of hypertension status. The cerebellum showed minimal WMH volumes bilaterally (<0.3 mm³ in normotensive participants), with only slight increases even in severe hypertension. Similarly, the posterior artery callosal (PAC) regions displayed consistently low WMH presence (left: 3.30 ± 19.56 mm³, right: 1.40 ± 10.09 mm³ in normotensive participants), with modest increases in severe hypertension (left: 15.56 ± 52.39 mm³, right: 6.41 ± 27.78 mm³ in Hypertensive Level 3). Notably, the fifth ventricle (also known as the cavum septi pellucidi, CSP) demonstrated negligible volumes across all groups (<0.1 mm³ in normotensive participants), reflecting the fact that this space is typically fused postnatally and persists in adults as a normal anatomical variant (Farruggia & Babcock, 1981).

### 3.2 Correlations between covariates and brain measures

The Spearman and point-biserial correlations between covariates and the 195 brain features in the training dataset are detailed in Supplementary Table S4 and illustrated in Supplementary Figure S3. All *p*-values reported are False Discovery Rate (FDR) corrected.

Age demonstrated distinct correlation patterns across different types of brain measures. For WMH volumes, age showed significant positive correlations across multiple features, with the strongest correlations observed in global measures including whole-brain WMH (*r* = 0.394, *p* < 0.001). In contrast, certain features such as the cerebellar WMH volumes exhibited weak or non-significant correlations with age (left: *r* = -0.052, *p* = 0.037; right: *r* = -0.038, *p* = 0.128). For morphometric measures, age showed significant negative correlations with whole-brain grey matter volume (*r* = -0.218, *p* < 0.001) and total subcortical volume (*r* = -0.285, *p* < 0.001), as well as cortical thickness in regions such as the left insula (*r* = -0.087, *p* < 0.001) and right insula (*r* = -0.134, *p* < 0.001).

Intracranial volume (ICV) showed strong positive correlations with brain structural volumes, including whole-brain subcortical grey matter (*r* = 0.771, *p* < 0.001) and whole-brain cortical grey matter (*r* = 0.853, *p* < 0.001), while showing weaker correlations with WMH volumes (whole-brain WMH: *r* = 0.086, *p* < 0.001) and cortical thickness measures (left insula: *r* = 0.090, *p* < 0.001; right insula: *r* = 0.109, *p* < 0.001). Similarly, sex demonstrated moderate to strong correlations with structural volumes, particularly whole-brain cortical grey matter (*r* = 0.545, *p* < 0.001) and subcortical grey matter (*r* = 0.471, *p* < 0.001), with generally weaker associations with WMH volumes and cortical thickness. Among vascular risk factors, diabetes showed the most consistent associations with WMH volumes (whole-brain: *r* = 0.081, *p* = 0.017), while hypercholesterolaemia demonstrated significant correlations with whole-brain WMH (*r* = 0.118, *p* < 0.001) and right insula thickness (*r* = -0.092, *p* = 0.001). Obesity and smoking status generally showed weak or non-significant correlations with most brain measures.

### 3.3 Covariate sensitivity analysis

**Fig. 3.**
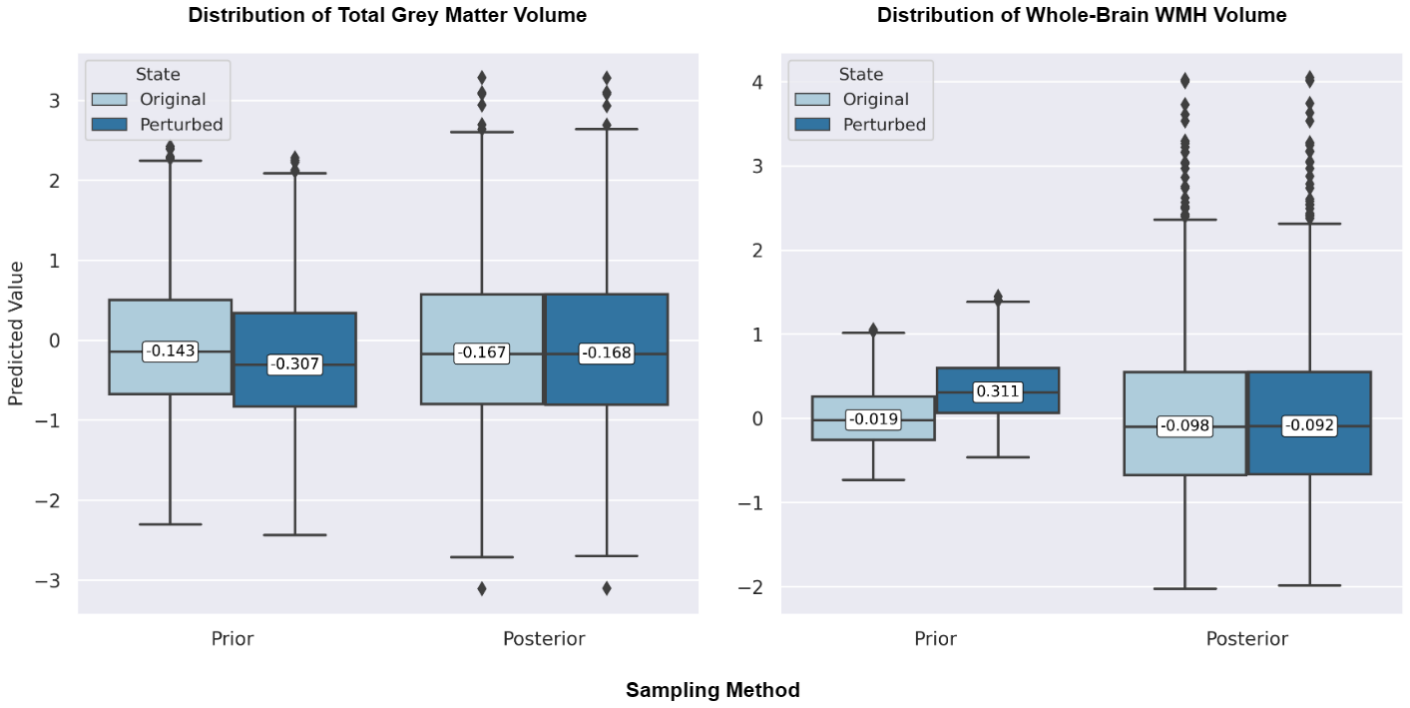
Box plots of brain volume predictions under age perturbation: Prior versus posterior sampling. These box plots illustrate the distribution of predicted total grey matter volume (standardised, left) and whole-brain WMH volume (log-transformed and standardised, right). The plots compare predictions from prior-sampling cVAE and posterior-sampling cVAE under Original and Age-Perturbed covariate conditions. Predictions were generated using the hold-out dataset (*n* = 1,711). WMH = White Matter Hyperintensity.

Our covariate sensitivity analysis revealed substantial differences in how the prior and posterior sampling approaches respond to age perturbations, reflecting distinct inference mechanisms within the cVAE framework. At the feature-specific level, we directly compared the medians of perturbed versus original predictions for each sampling method. As shown in Supplementary Table S5, prior-cVAE exhibited markedly stronger responses to age perturbation in almost all types of brain measures. For instance, median shifts (in standardised units) for prior sampling included -0.220 for volume of whole-brain subcortical grey matter, -0.164 for volume of whole-brain cortical grey matter, and 0.330 for volume of whole-brain WMH. Conversely, posterior sampling demonstrated minimal changes across all measure types, with median shifts of 0.007, -0.001, and 0.006 for the same measures, respectively. This contrast in sensitivity is illustrated in Figure 3 for both total grey matter volume (left panel) and whole-brain WMH volume (right panel), where prior sampling shows noticeable shifts in predicted values under age perturbation (from -0.143 to -0.307 for total grey matter; from -0.019 to 0.311 for WMH), while posterior sampling remains relatively stable (from -0.167 to -0.168 for total grey matter; from -0.098 to -0.092 for WMH).

In the global analysis comparing the magnitude of perturbation effects between sampling methods, we found that prior sampling exhibited significantly larger absolute differences between original and perturbed predictions (median: 0.126, IQR: 0.125) than posterior sampling (median: 0.023, IQR: 0.031). The Wilcoxon signed-rank test confirmed that posterior sampling was significantly less sensitive to age perturbations than prior sampling, with an effect size of -0.573 and a *p*-value < 0.001 (FDR-corrected). Posterior sampling showed 82.2% smaller perturbation effects than prior sampling, suggesting a substantial bias towards the observed data that limits its ability to capture covariate-driven variations.

### 3.4 Model performance metrics comparison

Table 3 presents the results of the median-based permutation tests, comparing the performance of our proposed prior-cVAE approach against posterior-cVAE, GAMLSS, MFPR, and HBR models using the following metrics: Median Absolute Error, Root Mean Squared Error, Spearman’s Correlation Coefficient between observed and predicted values, and Explained Variance. Detailed performance metric values are provided in Supplementary Table S6.

**Table 3.** Median-based permutation test results for comparing model performance across metrics on the hold-out dataset**.**

	Effect size	Permutation p-value (FDR-corrected)
Median Absolute Error:		
- Prior-cVAE vs. Posterior-cVAE	0.873	**<0.001**
- Prior-cVAE vs. GAMLSS	0.062	0.524
- Prior-cVAE vs. MFPR	0.006	1.0
- Prior-cVAE vs. HBR	-0.003	1.0
Root Mean Squared Error:		
- Prior-cVAE vs. Posterior-cVAE	0.907	**<0.001**
- Prior-cVAE vs. GAMLSS	-0.092	0.524
- Prior-cVAE vs. MFPR	0.018	1.0
- Prior-cVAE vs. HBR	0.004	1.0
Spearman’s Correlation Coefficient:		
- Prior-cVAE vs. Posterior-cVAE	-0.930	**<0.001**
- Prior- cVAE vs. GAMLSS	-0.007	0.524
- Prior- cVAE vs. MFPR	-0.012	1.0
- Prior- cVAE vs. HBR	0.006	1.0
Explained Variance:		
- Prior-cVAE vs. Posterior-cVAE	-0.875	**<0.001**
- Prior- cVAE vs. GAMLSS	-0.063	0.524
- Prior- cVAE vs. MFPR	-0.131	0.784
- Prior-cVAE vs. HBR	-0.070	1.0

This table presents results from median-based permutation tests, comparing the prior-sampling cVAE with other models (posterior-cVAE, GAMLSS, MFPR, and HBR) across various performance metrics. Cliff’s delta was used as the effect size measure. Permutation *p*-values indicate statistical significance by measuring how frequently random data shuffling produces differences larger than those observed, with lower values suggesting real differences rather than chance. Reported *p*-values were corrected for multiple comparisons using the False Discovery Rate (FDR) method. Bold p-values indicate statistical significance at the 0.05 level after FDR correction. cVAE = conditional Variational Autoencoder; GAMLSS = Generalised Additive Models for Location, Scale, and Shape; MFPR = Multivariate Fractional Polynomial Regression; HBR = Hierarchical Bayesian Regression.

The posterior-cVAE demonstrated substantially superior performance compared with all other models across all metrics, with statistically significant differences (*p* < 0.001). For Median Absolute Error, the posterior-cVAE achieved a large effect size of 0.873 than our prior-sampling approach, indicating markedly lower prediction errors. Similar patterns were observed for Root Mean Squared Error (effect size: 0.907), Spearman’s Correlation Coefficient (effect size: -0.930), and Explained Variance (effect size: -0.875). However, as discussed in Section 2.5, the posterior-cVAE uses both covariates and observed brain measures for prediction, giving access to additional information not available to other models.

Among the models using only covariates for prediction (prior-cVAE, GAMLSS, MFPR, and HBR), the differences were minor, with small effect sizes and high *p*-values. Comparisons between our proposed prior-cVAE and GAMLSS (effect size: 0.062, *p* = 0.524), MFPR (effect size: 0.006, *p* = 1.0), and HBR (effect size: -0.003, *p* = 1.0) showed no statistically significant differences in Median Absolute Error. Likewise, Root Mean Squared Error, Spearman’s Correlation Coefficient, and Explained Variance metrics indicated minimal and non-significant differences between our prior-cVAE and other covariate-only models, reflecting comparable performance in capturing overall trends and variance.

**Fig. 4.**
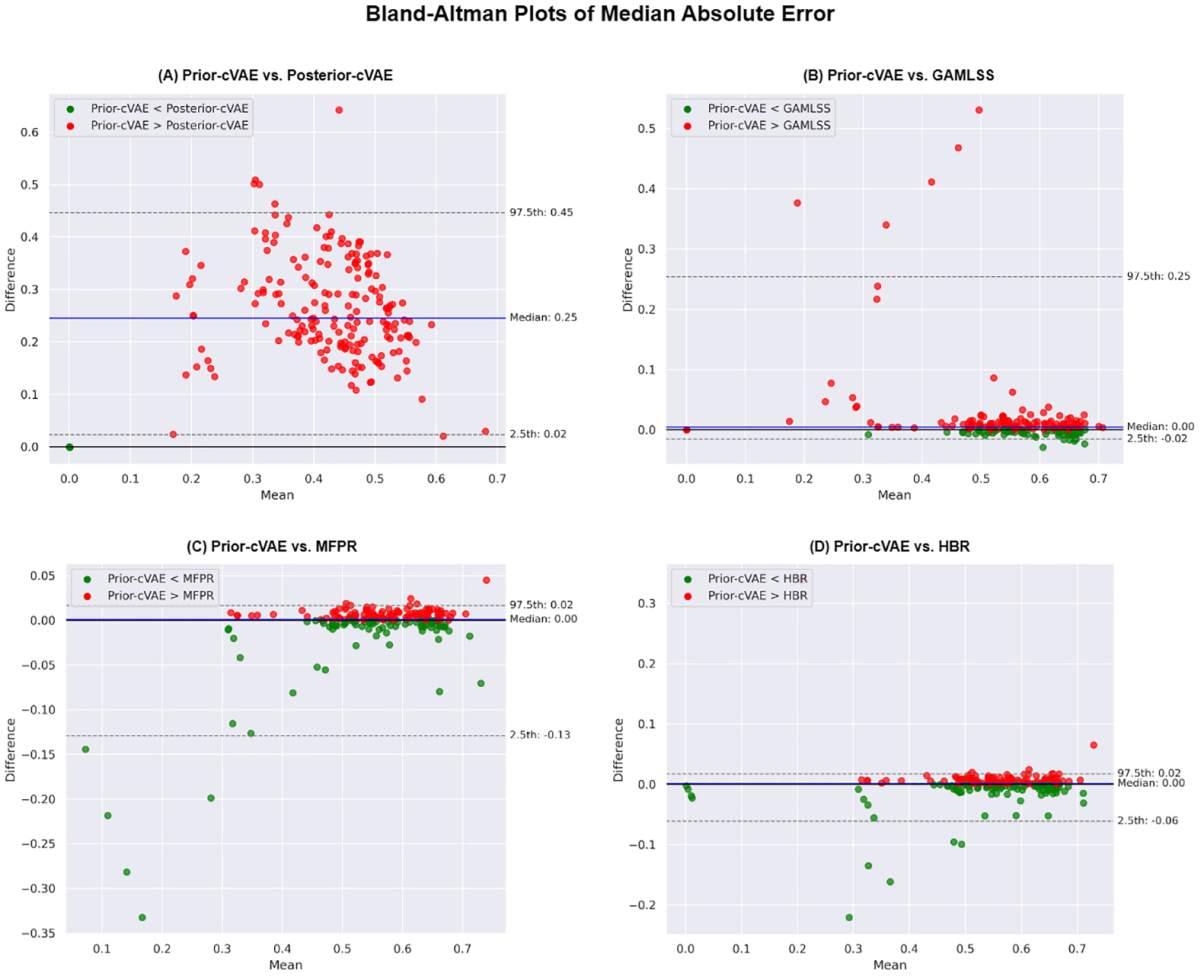
Bland–Altman plots and distribution of median absolute error for the prior-cVAE versus other models on the hold-out dataset. These plots compare the Median Absolute Error of the prior-sampling conditional Variational Autoencoder (cVAE) model against the posterior-sampling cVAE (Posterior-cVAE) (A), Generalised Additive Models for Location, Scale, and Shape (GAMLSS) (B), Multivariate Fractional Polynomial Regression (MFPR) (C), and Hierarchical Bayesian Regression (HBR) (D) models. Each point represents a different brain region. Green points indicate regions where cVAE exhibits lower Median Absolute Error than the corresponding model, while red points show regions where the other model performs better.

Figure 4A to D presents Bland–Altman plots that depict the distribution of differences in Median Absolute Error between our prior-cVAE and other models across different brain regions. Figure 4A illustrates the substantial performance gap between prior- and posterior-cVAE, with the posterior-sampling approach showing consistently lower errors across all brain regions. In contrast, Figure 4B to D shows that region-specific variations among covariate-only models are relatively minor, with the majority of brain features clustering around the median difference lines, suggesting generally comparable performance. Additional Bland–Altman plots for other performance metrics are shown in Supplementary Figures S4 and S5. Further details regarding the Explained Variance metric across brain features are provided in Supplementary Methods S4.

### 3.5 Expected calibration error analysis

**Fig. 5.**
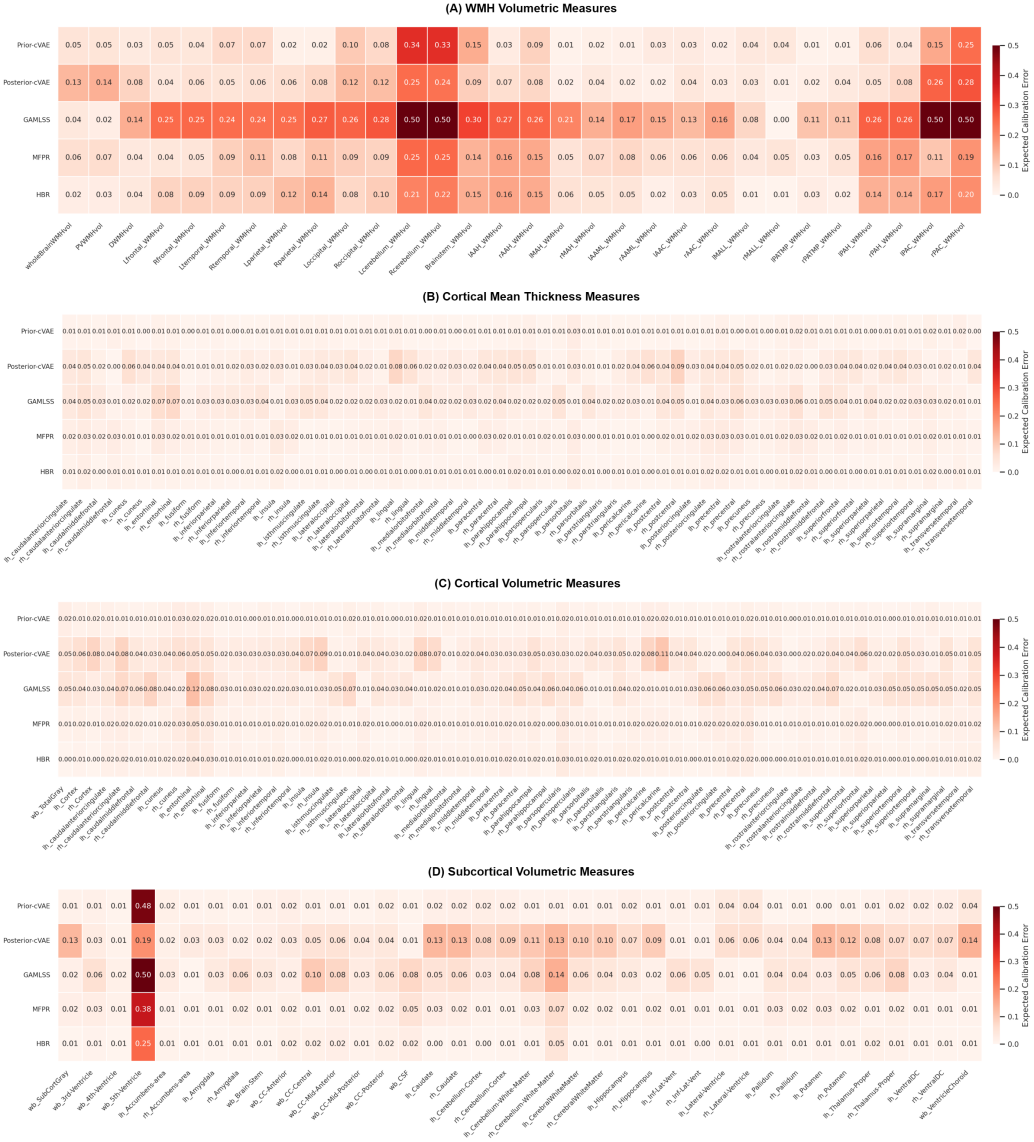
Expected calibration error (ECE) of brain measures and model (prior-cVAE, posterior-cVAE, GAMLSS, MFPR, HBR) on the hold-out dataset, shown separately for each measure category: (A) WMH Volumetric Measures, (B) Cortical Mean Thickness Measures, (C) Cortical Volumetric Measures, and (D) Subcortical Volumetric Measures. Lower ECE values (lighter colours) indicate better calibration, meaning the model’s uncertainty estimates align well with the actual error distribution. Higher values (darker colours) indicate poorer calibration, where models are either overconfident (producing confidence intervals that are too narrow) or underconfident (producing intervals that are too wide). cVAE = conditional Variational Autoencoder; GAMLSS = Generalised Additive Models for Location, Scale, and Shape; MFPR = Multivariate Fractional Polynomial Regression; HBR = Hierarchical Bayesian Regression; WMH = White Matter Hyperintensity. See Supplementary Table S1 and Supplementary Figure S1 for region-specific measure abbreviations.

Figure 5 presents the Expected Calibration Error (ECE) values across all brain measures for the five models (prior-cVAE, posterior-cVAE, GAMLSS, MFPR, and HBR).

For WMH measures (Fig. 5A), calibration performance varied substantially between global and regional metrics. Overall, most models achieved good to moderate calibration, with ECE values predominantly below 0.15. Notably, for key general WMH measures such as whole-brain and periventricular WMH volumes, the covariate-only models (prior-cVAE, GAMLSS, and HBR) exhibited better calibration (i.e., lower ECE values) than the dual-input posterior-cVAE approach. However, while GAMLSS demonstrated excellent calibration for global measures (ECE = 0.04 for whole-brain; 0.02 for periventricular), it performed less consistently across lobar and arterial territory regions, where ECE values frequently reached 0.15–0.39. Certain regions presented challenges for all models (ECE > 0.15), such as the cerebellum and posterior artery callosal WMH volumes. This high ECE indicates a mismatch between the models’ predicted probabilities and the observed outcome frequencies, likely due to sparse pathology in these areas.

For cortical mean thickness measures (Fig. 5B) and cortical volumetric measures (Fig. 5C), all models demonstrated substantially better calibration than WMH measures, with ECE values consistently at or near 0.01 across nearly all brain features. This improvement likely reflects the more normally distributed nature of cortical morphometric measures compared with the skewed WMH volumes. Similarly, subcortical volumetric measures (Fig. 5D) showed good to excellent calibration across most structures (ECE < 0.05). A notable exception was the 5th ventricle, which showed markedly higher ECE values across all models—0.48 (prior-cVAE), 0.19 (posterior-cVAE), 0.50 (GAMLSS), 0.38 (MFPR), and 0.25 (HBR)—indicating unreliable uncertainty estimates in this region.

### 3.6 Deviation analysis

#### 3.6.1 Covariate independence analysis

**Fig. 6.**
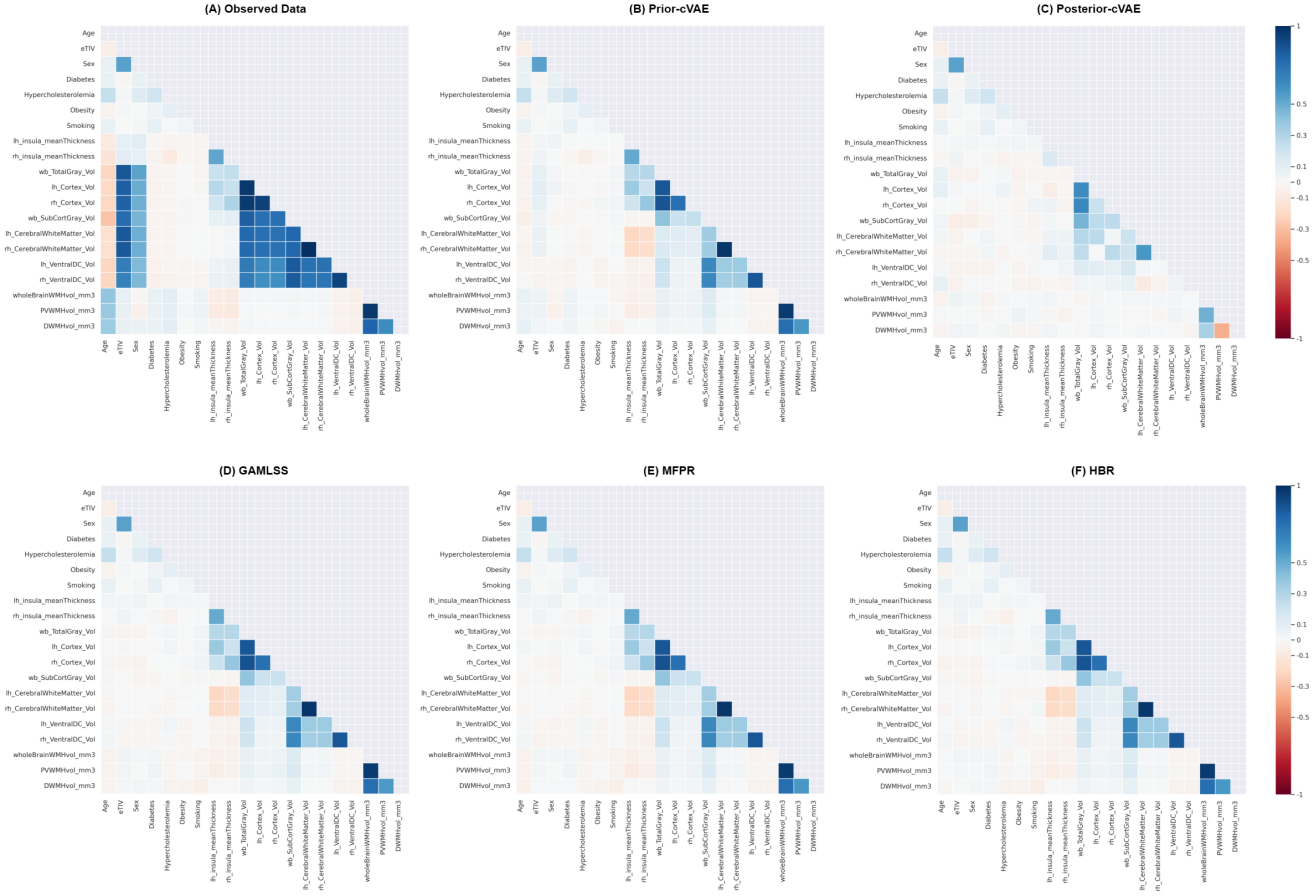
Heatmaps of Spearman correlation among covariates across observed and modelled data. This figure presents six heatmaps (A–F) showing the pairwise Spearman correlation coefficients between covariates and selected imaging-derived phenotypes for the hold-out dataset. Panel (A) displays the correlations observed in the empirical data, while panels (B–F) show correlations between deviation scores estimated by different approaches: (B) Prior-sampling conditional Variational Autoencoder (Prior-cVAE), (C) Posterior-sampling conditional Variational Autoencoder (Posterior-cVAE), (D) Generalised Additive Models for Location, Scale, and Shape (GAMLSS), (E) Multivariate Fractional Polynomial Regression (MFPR), and (F) Hierarchical Bayesian Regression (HBR). Blue indicates positive correlations and red indicates negative correlations, with colour intensity representing the strength of association. lh = left hemisphere; rh = right hemisphere; wb = whole-brain. See Supplementary Table S1 and Supplementary Figure S1 for brain region-specific measure abbreviations.

Figure 6 shows the pairwise correlations between covariates and deviation scores for representative brain measures from the hold-out dataset, with detailed coefficients provided in Supplementary Table S7. Overall, all models achieved substantially lower correlations between z-scores and covariates than true brain measures and covariates, indicating effective control of confounding effects and preservation of covariate independence in normative predictions.

Specifically, age correlations were minimal for all the representative features across all models (|* r *| < 0.05, *p* > 0.05; FDR-corrected). For example, in the prior-cVAE, correlations with age were −0.026 for left insula thickness, −0.025 for whole-brain cortical volume, −0.033 for total subcortical volume, and 0.041 for whole-brain WMH volume. Similarly, intracranial volume (ICV) showed weak correlations with thickness and volumetric measures, such as total cortical grey matter (|* r *| = 0.021–0.044, *p* > 0.05), total subcortical volume (|* r *| = 0.001–0.075, *p* > 0.05), and WMH volumes (|* r *| = 0.030–0.052, *p* > 0.05). Furthermore, sex and the vascular risk factors (diabetes, hypercholesterolemia, obesity, and smoking) exhibited negligible associations with all morphometric and WMH measures.

However, moderate age- and ICV-related correlations persisted in certain regions, indicating the incomplete removal of covariate effects in those areas. For example, the 5th ventricle’s volume showed a clear age association (prior-cVAE: *r* = 0.217, *p* < 0.001; GAMLSS: *r* = 0.140, *p* < 0.001). WMH measures in the posterior artery callosal (PAC) regions also retained non-trivial age correlations across models (left: |* r *| = 0.146–0.445; right: |* r *| = 0.046–0.548; all *p* < 0.001). Similarly, cerebellar WMH volumes showed persistent ICV dependencies, with MFPR-derived deviations correlating negatively with ICV (left: *r* = −0.557; right: *r* = −0.585; *p* < 0.001).

#### 3.6.2 Clinical application

**Fig. 7.**
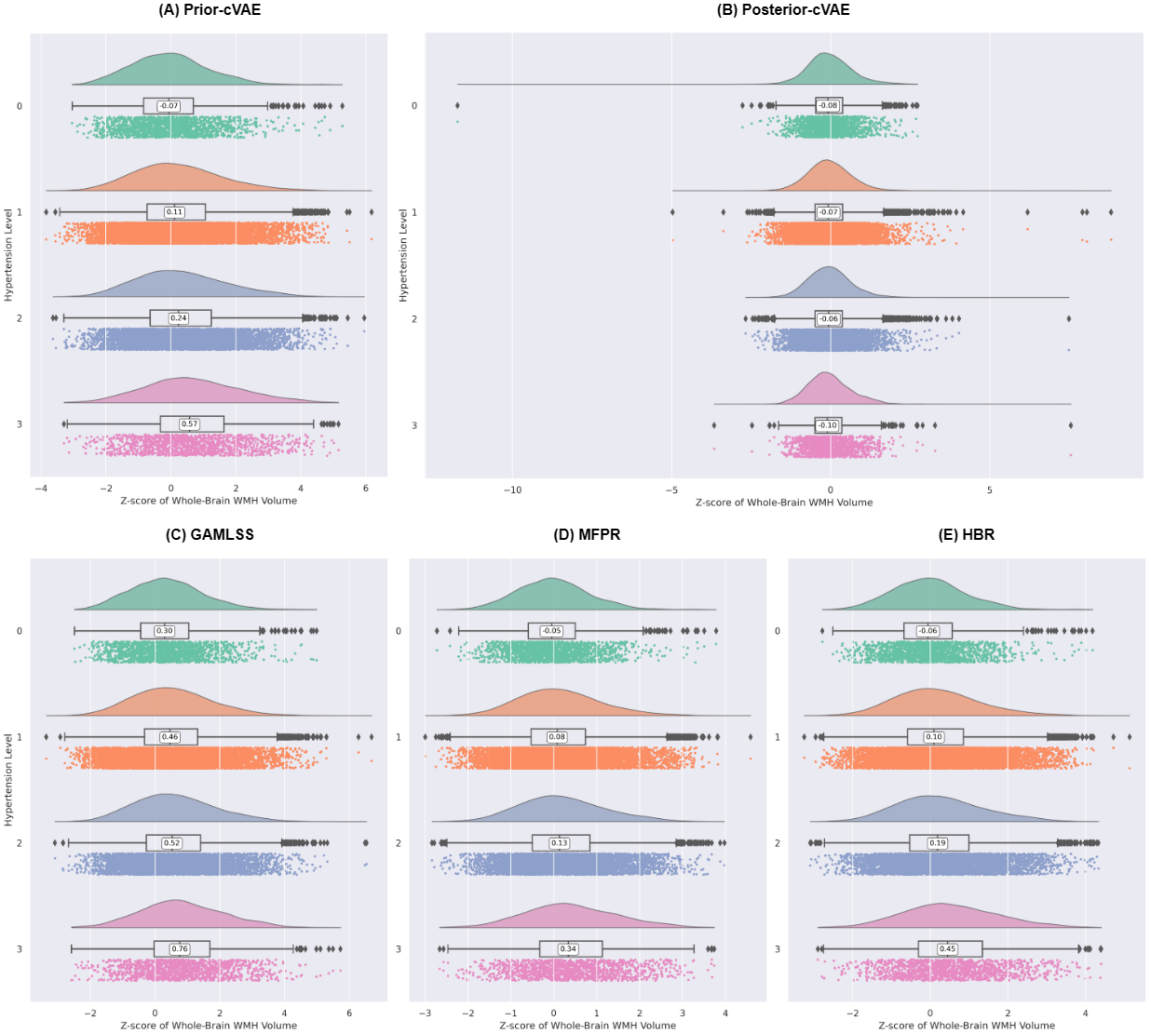
Comparison of Z-scores for whole-brain WMH volume across hypertensive levels and models. This figure displays the distribution of z-scores for whole-brain WMH volume stratified by hypertensive levels (0–3) across five different modelling approaches. Each panel corresponds to a different model: (A) Prior-sampling conditional Variational Autoencoder (prior-cVAE), our proposed inference approach that generates brain measures directly from covariates; (B) Posterior-sampling cVAE, the conventional inference approach that uses both observed data and covariates (posterior-cVAE); (C) Generalised Additive Models for Location, Scale, and Shape (GAMLSS); (D) Multivariate Fractional Polynomial Regression (MFPR); and (E) Hierarchical Bayesian Regression (HBR).

The clinical relevance of the deviation scores derived from each normative model was examined by evaluating their associations with hypertension severity. Figure 7 shows the distribution of whole-brain WMH volume z-scores across hypertension levels (0–3) for the five modelling approaches. Notably, four covariate-only models (prior-cVAE, GAMLSS, MFPR, and HBR) displayed consistent positive trends, with higher hypertension levels associated with increasingly elevated z-scores. This indicates that all four models captured the expected hypertensive burden on white matter. In contrast, the posterior-cVAE exhibited minimal variation, with z-scores remaining centred around zero regardless of hypertension severity. Quantitatively, Supplementary Table S8 confirmed weak but significant positive Spearman’s correlations between whole-brain WMH z-scores and hypertension levels for the four covariate-only models (*r* = 0.072–0.094, *p* < 0.001 after FDR correction), whereas the dual-input posterior-cVAE showed no significant association (*r* = −0.001, *p* = 0.983).

**Fig. 8.**
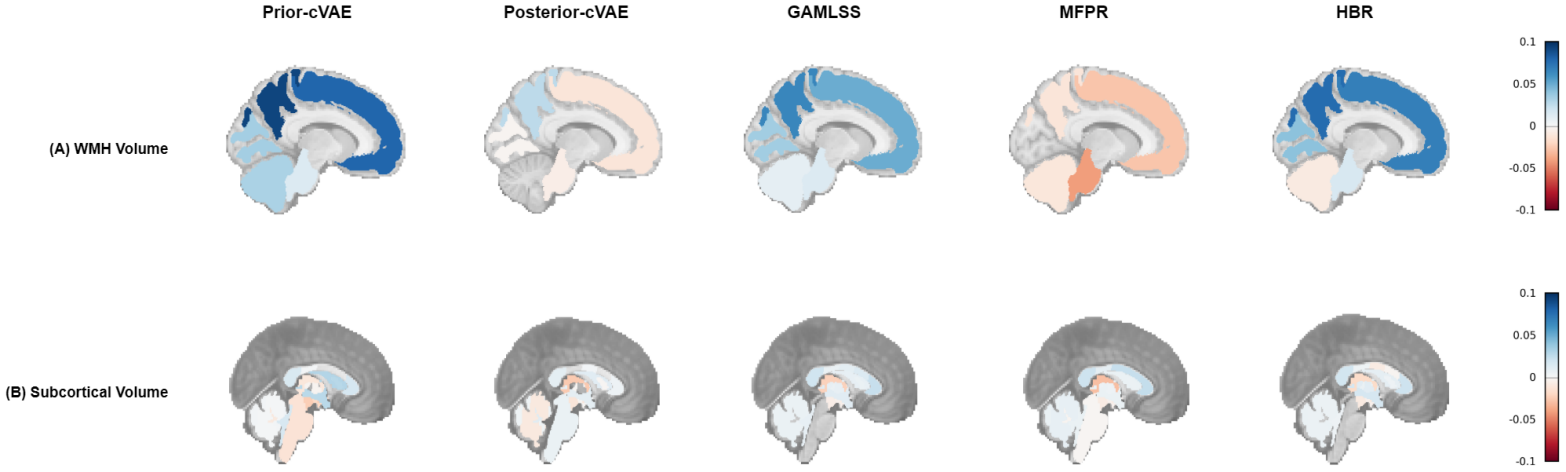
Correlation between Z-scores and hypertension severity across models: Lobar WMH and subcortical volumes. Spearman correlation coefficients between model-derived z-scores and hypertension levels for five models (Prior-cVAE, Posterior-cVAE, GAMLSS, MFPR, HBR) on the evaluation dataset. (A) Lobar WMH volume correlations (sagittal slice 10). (B) Subcortical volume correlations (sagittal slice 0). Red indicates negative correlations, and blue indicates positive correlations (range: -0.1 to +0.1). cVAE = conditional Variational Autoencoder; GAMLSS = Generalised Additive Models for Location, Scale, and Shape; MFPR = Multivariate Fractional Polynomial Regression; HBR = Hierarchical Bayesian Regression; WMH = White Matter Hyperintensity.

Beyond global WMH measures, regional analyses provided more detailed insights into the spatial patterns of hypertension-related white matter abnormalities. Specifically, both lobar and arterial territory WMH volumes showed statistically significant positive correlations with hypertension severity across covariate-only models (*p* < 0.001), with the notable exception that MFPR demonstrated negative correlations in lobar regions while maintaining positive associations in arterial territories (Fig. 8A; Supplementary Fig. S7A; Supplementary Table S8). Among lobar regions, frontal and parietal lobes exhibited the strongest positive correlations in prior-cVAE (frontal: *r* = 0.075–0.079; parietal: *r* = 0.088–0.092, all *p* < 0.001), GAMLSS (frontal: *r* = 0.045–0.050; parietal: *r* = 0.054–0.065, all *p* < 0.001), and HBR (frontal: *r* = 0.066–0.068; parietal: *r* = 0.070–0.077, all *p* < 0.001). MFPR showed negative associations across all lobes, with the strongest negative correlations observed in frontal regions (left: *r* = −0.035, right: *r* = −0.028; both *p* < 0.001). For arterial territories, all covariate-only models demonstrated consistent positive correlations, with the middle artery lateral lenticulostriate showing the most robust associations (*r* = 0.090–0.092 for prior-cVAE, *r* = 0.068–0.073 for GAMLSS, *r* = 0.072–0.076 for MFPR, and *r* = 0.072–0.080 for HBR; all *p* < 0.001), followed by the middle artery hemisphere, anterior artery hemisphere, and their corresponding territories. In contrast, posterior-cVAE displayed near-zero or slightly negative correlations across all WMH measures, whether lobar or arterial territories.

Compared with WMH volumes, subcortical volume measures (Fig. 8B) showed lower correlation magnitudes and exhibited more heterogeneous spatial patterns across models. For instance, ventricular structures, including the inferior lateral ventricles (left: *r* = 0.020–0.040; right: *r* = 0.018–0.032, *p* < 0.001), showed positive correlations, reflecting ventricular expansion associated with increasing hypertension severity. Other subcortical regions, such as the hippocampus (|* r *| ≤ 0.017), displayed weaker or inconsistent associations. The posterior-cVAE again demonstrated minimal sensitivity, with near-zero correlations across all subcortical structures.

**Fig. 9.**
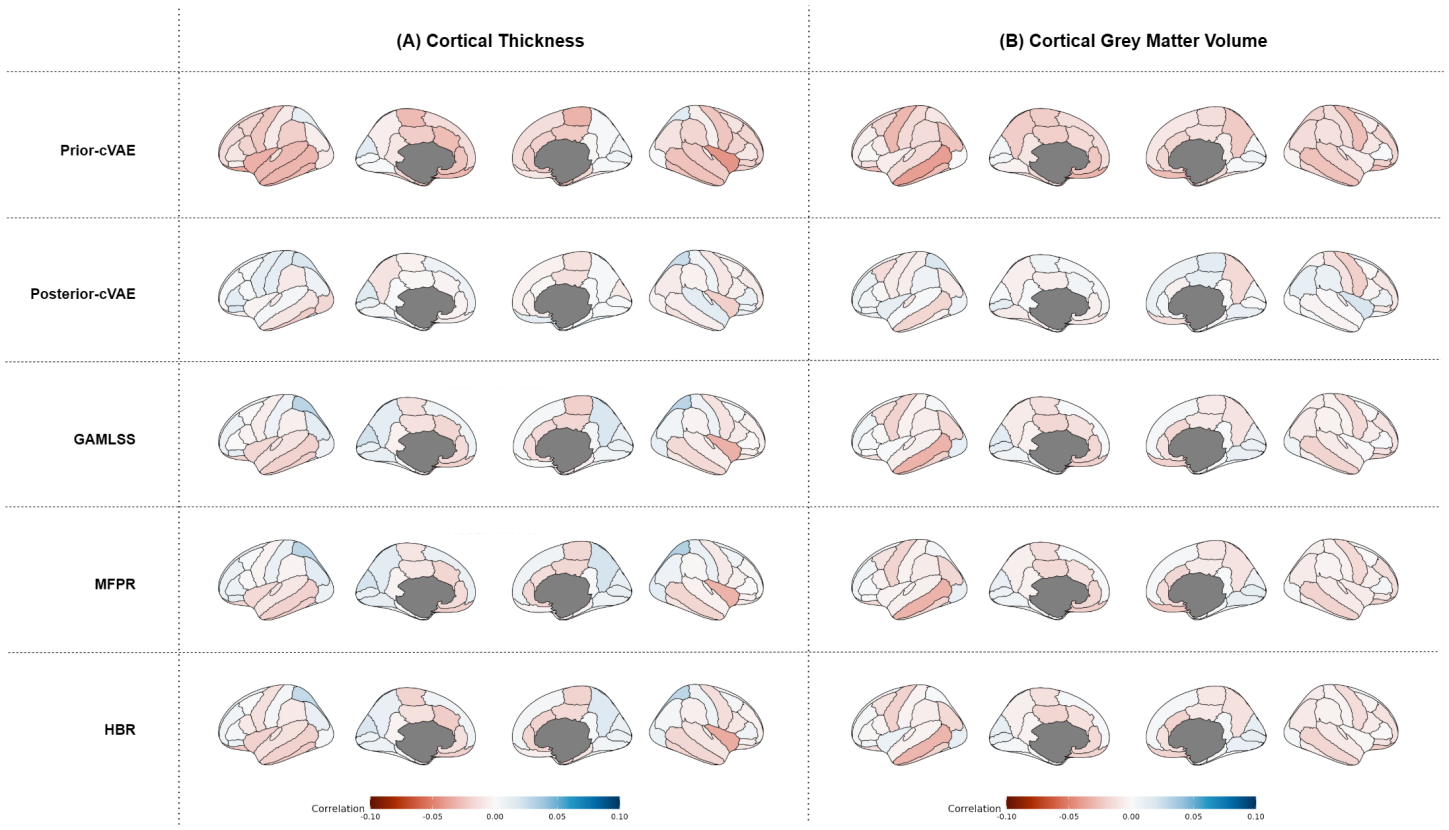
Regional cortical correlations between Z-scores and hypertension severity across models. Spearman correlation coefficients between model-derived z-scores and hypertension levels for five models (Prior-cVAE, Posterior-cVAE, GAMLSS, MFPR, HBR) on the evaluation dataset, displayed across four brain views (lateral left, medial left, medial right, lateral right). (A) Cortical thickness correlations using DKT parcellation. (B) Cortical grey matter volume correlations using DKT parcellation. Red indicates negative correlations, and blue indicates positive correlations (range: -0.1 to +0.1). Grey regions represent subcortical structures not included in cortical analyses. cVAE = conditional Variational Autoencoder; GAMLSS = Generalised Additive Models for Location, Scale, and Shape; MFPR = Multivariate Fractional Polynomial Regression; HBR = Hierarchical Bayesian Regression; DKT = Desikan–Killiany–Tourville.

For cortical thickness (Fig. 9A), z-scores displayed predominantly negative correlations with hypertension levels across all models, with posterior-cVAE showing lower magnitude of correlation than covariate-only models. Additionally, the prior-cVAE revealed more spatially distributed and statistically significant associations between deviation scores and hypertension level. Supplementary Table S8 indicates that prior-cVAE identified significant correlations in approximately 22 out of 62 cortical thickness regions, while GAMLSS, MFPR, and HBR showed significance in only 2–6 features. These associations were generally weaker than those observed for WMH volumes, reflecting the more subtle nature of hypertension-related cortical changes. Among the regions showing consistent negative associations, the right insula’s cortical thickness showed slightly stronger negative associations, with statistically significant correlations in all four covariate-only models (prior-cVAE: *r* = −0.043, *p* < 0.001; GAMLSS: *r* = −0.033, *p* < 0.001; MFPR: *r* = −0.032, *p* = 0.001; HBR: *r* = −0.035, *p* < 0.001).

Cortical grey matter volume correlations (Fig. 9B; Supplementary Fig. S6; Supplementary Table S8) demonstrated similar trends: all covariate-only models demonstrated negative correlations with increasing hypertension level, though with lower correlation magnitudes than WMH measures. Notably, the left middle temporal gyrus’s volumes showed consistent negative associations across all four covariate-only models (prior-cVAE: *r* = −0.040, *p* < 0.001; GAMLSS: *r* = −0.023, *p* = 0.041; MFPR: *r* = −0.024, *p* = 0.032; HBR: *r* = −0.023, *p* = 0.042). The prior-cVAE also identified additional weak yet significant associations across temporal, frontal, and cingulate regions (|* r *| < 0.03). In contrast, the posterior-cVAE consistently showed near-zero correlations across all cortical measures, with no statistically significant associations in any region.

The extreme deviation analysis (Supplementary Figs. S8 and S9; Supplementary Tables S9 and S10) complements the Spearman correlation findings by examining extreme deviations at the individual level. For WMH measures, whole-brain volume extreme deviations (z-score > 2.58) progressively increased with hypertension severity: from 2.28% (level 0) to 10.93% (level 3) for prior-cVAE, from 3.16% to 10.01% for GAMLSS, from 0.70% to 3.26% for MFPR, and from 1.17% to 5.75% for HBR. For morphometric measures, negative extreme deviations (z-score < −2.58) in cortical thickness and volumes remained relatively stable across hypertension levels for all models, typically below 2%. Consistent with its flat correlation patterns, the posterior-cVAE showed no extreme deviations across any hypertension level, consistent with its lack of significant Spearman correlations. Notably, certain regions exhibited higher percentages of extreme deviations across all models regardless of hypertension level, reflecting inherent modelling challenges rather than true pathology. This was particularly evident for positive extreme deviations (z-score > 2.58) in WMH volumes of the posterior artery callosal (PAC) regions (7–77%), where GAMLSS showed exceptionally high rates (53–78%). A similar pattern was observed in the 5th ventricle volume, where GAMLSS again displayed higher rates (>50% extreme deviations in both directions), whereas the other models maintained low rates (<3%).

### 3.7 Computational efficiency

Table 4 compares the computational requirements across all models in terms of training and inference times. All models were trained using 24 CPU cores on a standardised computational setup. The non-cVAE models (GAMLSS, MFPR, and HBR) employed a feature-wise modelling approach, fitting independent models for two features simultaneously via multithreading. The cVAE models (both prior-sampling and posterior-sampling) utilised two additional GPUs for parallelisation during hyperparameter tuning via the Optuna framework.

**Table 4.** Elapsed time comparison across models during training and inference.

	Prior-cVAE	Posterior-cVAE	GAMLSS	MFPR	HBR
Training (seconds)	5,642	5,642	4,297	1,887	85,070
Inference (seconds)	3,763	3,784	59	46	2,383
Total (seconds)	9,405	9,426	4,356	1,933	87,453

This table compares the computational efficiency of five modelling approaches: prior-cVAE, posterior-cVAE, GAMLSS, MFPR, and HBR. cVAE = conditional Variational Autoencoder; GAMLSS = Generalised Additive Models for Location, Scale, and Shape; MFPR = Multivariate Fractional Polynomial Regression; HBR = Hierarchical Bayesian Regression.

The results revealed substantial variations in computational requirements among the models. MFPR was the most computationally efficient, with a total elapsed time of 1,933 seconds (approximately 32 minutes), followed by GAMLSS at 4,356 seconds (approximately 73 minutes). The prior-cVAE required 9,405 seconds (approximately 2.6 hours), while the posterior-cVAE was slightly slower at 9,426 seconds (approximately 2.6 hours), primarily due to the added complexity of encoding observed brain measures during inference. In contrast, HBR demanded substantially greater computational resources, requiring 87,453 seconds (approximately 24.3 hours) for the complete training and inference pipeline, making it approximately 9 times slower than the cVAE approaches and 45 times slower than MFPR.

## 4 Discussion

A key contribution of this study is the development and refinement of the cVAE-based normative model, with a particular focus on integrating probabilistic inference through our prior-sampling strategy. Unlike existing autoencoder-based normative models, our approach provides a more direct way to model normative variations, rather than using reconstruction error as a proxy for detecting abnormalities. Our prior-sampling inference strategy generates predictions directly from covariates without incorporating observed brain measures, thereby preserving the core principle that normative predictions should reflect only what is expected based on demographic and clinical characteristics. The advantage of this inference strategy was demonstrated in the covariate sensitivity analysis, which revealed that prior sampling shows substantially higher sensitivity to age-related variability than conventional posterior sampling approaches. Without being constrained by observed brain measures, the prior-sampling inference strategy allows the covariate effects learned during training to be more fully expressed. In contrast, posterior sampling, which incorporates observed brain measures, showed minimal response to covariate changes. This indicates a potential bias in posterior sampling towards the observed data, which can lead to an underestimation of covariate effects such as age. Such insensitivity could limit the model’s ability to detect clinically meaningful deviations linked to individual differences in covariates, undermining its effectiveness in normative modelling applications.

In addition to establishing the methodological advantages of prior sampling, this study provides the first systematic comparison of deep learning against established statistical approaches in normative modelling. As detailed in Section 3.5, which compares model performance across quantitative metrics, our results demonstrate that the prior-cVAE achieved performance comparable with well-established methods including GAMLSS, MFPR, and HBR across all metrics. Notably, the posterior-cVAE yielded numerically lower prediction errors across all metrics than covariate-only models. However, this comparison is fundamentally unfair: by incorporating observed brain measures during inference, posterior sampling gains access to information unavailable to true normative models. This dual-input approach explains its excellent reconstruction accuracy but simultaneously undermines its validity as a normative model, as the predicted values are not independent of the observations they are intended to benchmark.

Beyond predictive accuracy, the quality of uncertainty estimates proved crucial for reliable deviation quantification. The calibration analysis showed that all models, including prior-cVAE, achieved excellent uncertainty estimates for morphometric measures. This indicates that the predicted confidence intervals closely matched the observed error distributions. For WMH measures, however, calibration performance varied more widely across both regions and models. While covariate-only models demonstrated superior calibration for global WMH measures than the dual-input posterior-cVAE approach, notable differences emerged among the covariate-only methods themselves. GAMLSS, despite achieving excellent calibration for whole-brain and periventricular WMH volumes, exhibited considerably poorer performance across lobar and arterial territory regions. In contrast, prior-cVAE, MFPR, and HBR maintained more consistent calibration across diverse WMH regions. All models, however, faced challenges in regions with sparse pathology, such as the cerebellar and posterior artery callosal WMH volumes, and the fifth ventricle’s volume. These calibration patterns suggest that while our approach produces reliable uncertainty estimates for most brain regions, special consideration is needed when interpreting deviations in areas with limited pathological representation in the training data.

The clinical validation through hypertension analysis provided compelling evidence that our prior-sampling approach generates deviation scores capable of detecting clinically meaningful abnormalities. Across all covariate-only models, including our proposed prior-cVAE, deviation scores demonstrated progressive associations with hypertension severity, with white matter hyperintensity measures showing the most robust relationships and spatially extensive relationships. This pattern aligns with established literature indicating that hypertension’s impact on brain structure is more pronounced in white matter pathology than in cortical atrophy, reflecting the particular vulnerability of small vessels to chronic blood pressure elevation (Du et al., 2024; Hainsworth et al., 2024). The prior-cVAE detected significant correlations between WMH z-scores and hypertension levels across whole-brain, periventricular, deep, and regional measures, with extreme deviations increasing systematically from normotensive to severe hypertensive groups. Interestingly, MFPR unexpectedly exhibited negative correlations in several lobar WMH measures, likely attributable to its reliance on RMSE-based uncertainty estimation.

For morphometric measures, associations with hypertension were more modest but anatomically meaningful. Cortical thickness demonstrated predominantly negative correlations with hypertension levels, with the prior-cVAE revealing more spatially distributed and statistically significant associations than other covariate-only models. Cortical grey matter volumes exhibited similar patterns, with the prior-cVAE again identifying substantially more significant regions than GAMLSS, MFPR, or HBR. Among these regions, right insular cortical thickness and middle temporal gyrus volumes showed consistent negative associations across all covariate-only models, patterns that align with previous findings demonstrating the vulnerability of these regions to hypertensive damage (Sanchez-Larsen et al., 2024; Won et al., 2024). Subcortical structures displayed selective vulnerability, with ventricular expansion showing modest hypertension-related increases, whereas other structures showed only weak or non-significant associations. Overall, the prior-cVAE demonstrated the broadest spatial extent of significant associations among covariate-only models, identifying clinically relevant deviations across more cortical and subcortical regions than GAMLSS, MFPR, or HBR. This enhanced sensitivity in detecting hypertension-related morphometric alterations suggests that joint feature modelling may capture subtle structural changes that escape detection by feature-wise statistical approaches.

In contrast, the posterior-cVAE failed to detect any meaningful hypertension-related abnormalities, showing near-zero correlations and minimal extreme deviations across all hypertension levels and brain regions. This lack of association occurs because, during inference, the posterior-cVAE conditions on both the covariates and the observed brain measures themselves, thereby incorporating direct information about the subject’s actual anatomy into the prediction. Consequently, the model reconstructs each individual’s data with such fidelity that residuals, and, therefore, deviation scores, become negligible even for pathological features. Additionally, since well-trained autoencoder and variational autoencoder models can accurately reconstruct even anomalous data (Bouman & Heskes, 2025), this makes it difficult to balance reconstruction accuracy with deviation sensitivity. Consequently, even though the posterior-cVAE achieves superior reconstruction metrics on the hold-out dataset, it produces very small residuals between observed and predicted values even in pathological features, thereby yielding deviation scores that are uninformative with respect to clinical variation. These findings underscore that high reconstruction accuracy does not equate to better normative performance. They also validate that our prior-sampling strategy better preserves the essential property of clinical sensitivity, which is the central objective of normative modelling.

Despite these promising results, several limitations require consideration. First, our model demonstrated challenges in regions with sparse pathology, particularly the fifth ventricle volumes, WMH in the cerebellum and the posterior artery callosal regions. These areas exhibited high rates of extreme deviations and persistent covariate dependencies in z-scores regardless of clinical status. This limitation affects all modelling approaches examined in this study, highlighting a fundamental challenge when modelling rare or focal abnormalities. Second, the calibration analysis revealed difficulties with white matter hyperintensity measures in certain regions, despite our application of log transformation prior to standardisation. The inherently non-Gaussian distribution of WMH volumes, characterised by excessive zeros and heavy right tails, proved challenging to fully normalise. This resulted in Expected Calibration Errors exceeding 0.15 in several WMH measures. Third, our current implementation does not explicitly disentangle aleatoric and epistemic uncertainty. The total predictive variance in our framework, consistent with HBR, jointly accounts for both sources of uncertainty. However, because latent variables are sampled from the prior, both the within- and between-iteration variances partially reflect model uncertainty. Consequently, the derived standard deviations represent the combined effect of aleatoric and epistemic components rather than their explicit decomposition. Finally, the computational demands of our approach exceed those of efficient statistical methods such as GAMLSS and MFPR. Although the cVAE’s training and inference time remained substantially lower than HBR, the requirement for iterative sampling and the complexity of deep learning architectures present practical barriers for applications requiring rapid deployment or limited computational resources. This trade-off between multivariate modelling capability and computational efficiency should inform method selection based on specific analytical needs and available infrastructure.

Several avenues for future research could address the limitations identified in this study and extend the applicability of our framework. First, to improve performance in regions with sparse pathology, future work could explore region-specific modelling strategies, such as incorporating additional anatomical or physiological predictors, or implementing adaptive training procedures that account for class imbalance in pathological representation. Second, addressing both the non-Gaussian distribution of white matter hyperintensity measures and computational efficiency represents a key priority for future work. Deviation scores could be derived directly from cumulative distribution functions (CDFs) of the generated samples, providing a percentile-based approach that naturally handles non-Gaussian distributions without requiring parametric assumptions or transformations. Third, as our current inference approach captures total predictive uncertainty without explicitly disentangling it into aleatoric and epistemic sources, future work could extend this decomposition to provide more transparent and clinically interpretable uncertainty estimates. Implementing techniques such as ensemble methods, Bayesian neural networks, or Monte Carlo Dropout could provide clearer separation and quantification of epistemic and aleatoric uncertainty, enabling clinicians to distinguish between regions where the model lacks confidence versus regions with genuine biological variability. Fourth, extending the framework to accommodate higher-dimensional data represents a natural next step. Incorporating convolutional neural network architectures would enable direct analysis of 2D slices or 3D brain volumes at the voxel level, fully leveraging the multivariate modelling advantages of deep learning. Finally, our prior-sampling inference strategy could be adapted to other generative architectures, such as diffusion models and normalising flows, potentially offering improved modelling flexibility while maintaining alignment with traditional normative modelling principles. These extensions would broaden the framework’s applicability across diverse neuroimaging modalities and clinical contexts.

## 5 Conclusion

In conclusion, this study makes two fundamental contributions to normative modelling in neuroimaging. First, we introduce an enhanced cVAE-based framework that employs prior-sampling inference to generate predictions directly from covariates, without incorporating observed brain measures. This approach addresses critical limitations in existing autoencoder-based normative models, which rely on posterior sampling that compromises their ability to serve as independent reference standards for deviation quantification. Second, this study provides the first comprehensive benchmarking of deep learning against established statistical methods in normative modelling. Through systematic comparison with GAMLSS, MFPR, and HBR, we demonstrate that our prior-cVAE achieves comparable predictive performance while maintaining more consistent behaviour across different brain regions and clinical associations. Unlike GAMLSS, which showed poor calibration in certain lobar and arterial territory WMH regions, and MFPR, which exhibited unexpected negative correlations in several lobar WMH measures with hypertension, the prior-cVAE demonstrated stable performance across diverse brain structures. Furthermore, while HBR achieved robust performance, its substantial computational demands limit practical applicability, whereas the prior-cVAE offers a more computationally efficient alternative without sacrificing accuracy. These findings provide evidence-based guidance for method selection: traditional statistical approaches remain well suited for individual feature modelling where computational efficiency is paramount, while deep learning offers a viable alternative when analysing high-dimensional data with potential multivariate dependencies, such as voxel-level 2D or 3D brain imaging. Together, these contributions advance the application of generative deep learning models in normative modelling, offering potential for personalised brain health assessment and early detection of neurological disorders in clinical practice.

## Supplementary Material

Supplementary Material

## Data Availability

The UK Biobank dataset is available to researchers through a formal application process (https://www.ukbiobank.ac.uk/). The code of our enhanced cVAE framework is available at https://github.com/maiho24/BrainNormativeCVAE. Our implementation extends the normative modelling framework by Lawry Aguila et al. (2022) (https://github.com/alawryaguila/normativecVAE), with substantial refinements in both the model architecture and inference approach.
