# Supplementary material for: A prior-sampling conditional variational autoencoder for neuroimaging normative modelling: Benchmarking deep learning against statistical approaches
Source: Imaging Neurosci (Camb). 2026 Jan 12;4:IMAG.a.1098. doi: 10.1162/IMAG.a.1098 (PMC12797146; doi:10.1162/IMAG.a.1098)
Supplement: Supplementary Material [file IMAG.a.1098_supp.pdf]

Supplementary Tables 3

Table S1 List of WMH features and corresponding abbreviations. 3

Table S2 List of exclusion codes and corresponding diseases from UK Biobank dataset. 5

Table S3 Descriptive statistics of brain features. 7

Table S4 Correlations between covariates and selected features using Spearman and point-biserial tests in the training dataset. 16

Table S5 Median predictions of selected brain measures before and after age perturbation on the hold-out dataset. 17

Table S6 Summary of model performance metrics across brain regions and performance metrics. 18

Table S7 Correlations between covariates and z-score of selected measures by model on the hold-out dataset. 26

Table S8 Spearman's correlations between Z-scores of brain measures and hypertension levels across models. 29

Table S9 Percentage of positive extreme deviations (%) of selected brain measures across models. 40

Table S10 Percentage of negative extreme deviations (%) of selected brain measures across models. 42

Supplementary Figures 44

Figure S1 Desikan-Killiany-Tourville (DKT) and Automated Subcortical Segmentation (ASEG) Atlases 44

Figure S2 Posterior Sampling Inference Approach in Conventional cVAE-based Normative Modelling 46

Figure S3 Heatmaps of Spearman Correlations Between Covariates and Brain Region-Specific Measures in the Training Dataset. 48

Figure S4 Bland-Altman Plots of Root Mean Squared Error for prior-cVAE vs Other Models. 52

Figure S5 Bland-Altman Plots of Spearman Correlation for prior-cVAE vs Other Models. 54

Figure S6 Comparison of Z-Scores for Whole-Brain Grey Matter Volume across Hypertensive Levels and Models. 56

Figure S7 Spearman Correlation between Z-score and Hypertension levels. 58

Figure S8 Comparison of Positive Extreme Deviations across Models and Hypertension Levels. 61

Figure S9 Comparison of Negative Extreme Deviations across Models and Hypertension Levels. 63

Supplementary Methods 65

S1 UBO Detector Pipeline 65

S1.1 Pipeline Overview 65

S1.2 Quality Control Procedures 66

S1.3 Application to UK Biobank Data 67

S2 Hyperparameter Optimisation with Optuna 68

S2.1 Optuna Framework Overview 68

S2.2 Optimisation Process 68

S2.3 Hyperparameter Search Space 69

S2.4 Optimisation Results 69

S3 Analysis of Training and Validation Loss Plot 70

S4 Explained Variance as a Performance Metric 72

Methodology 72

Results and Discussion 72

Reference 80

Supplementary Tables

Table S1 List of WMH features and corresponding abbreviations.

Feature	Definition
**General WMH volumetric measures:**	
- wholeBrainWMHvol	Total volume of WMH in the entire brain (mm^3)
- PVWMHvol	WMH volume of the periventricular areas (mm^3)
- DWMHvol	Total volume of deep WMH (mm^3)
**Lobe-specific WMH volumetric measures:**	
- lFrontal_WMHvol	WMH volume of the left frontal lobe (mm^3)
- rFrontal_WMHvol	WMH volume of the right frontal lobe (mm^3)
- lTemporal_WMHvol	WMH volume of the left temporal lobe (mm^3)
- rTemporal_WMHvol	WMH volume of the right temporal lobe (mm^3)
- lParietal_WMHvol	WMH volume of the left parietal lobe (mm^3)
- rParietal_WMHvol	WMH volume of the right parietal lobe (mm^3)
- lOccipital_WMHvol	WMH volume of the left occipital lobe (mm^3)
- rOccipital_WMHvol	WMH volume of the right occipital lobe (mm^3)
- lCerebellum_WMHvol	WMH volume of the left cerebellum (mm^3)
- rCerebellum_WMHvol	WMH volume of the right cerebellum (mm^3)
- Brainstem_WMHvol	WMH volume of the brainstem (mm^3)

**Arterial territory-specific WMH volumetric measures:**	
- lAAH_WMHvol	WMH volume of the left anterior artery hemisphere (mm^3)
- rAAH_WMHvol	WMH volume of the right anterior artery hemisphere (mm^3)
- lMAH_WMHvol	WMH volume of the left middle artery hemisphere (mm^3)
- rMAH_WMHvol	WMH volume of the right middle artery hemisphere (mm^3)
- lAAML_WMHvol	WMH volume of the left anterior medial lenticulostriate (mm^3)
- rAAML_WMHvol	WMH volume of the right anterior medial lenticulostriate (mm^3)
- lAAC_WMHvol	WMH volume of the left anterior callosal (mm^3)
- rAAC_WMHvol	WMH volume of the right anterior callosal (mm^3)
- lMALL_WMHvol	WMH volume of the left middle artery lateral lenticulostriate (mm^3)
- rMALL_WMHvol	WMH volume of the right middle artery lateral lenticulostriate (mm^3)
- lPATMP_WMHvol	WMH volume of the left posterior artery thalamic and midbrain perforators (mm^3)
- rPATMP_WMHvol	WMH volume of the right posterior artery thalamic and midbrain perforators (mm^3)
- lPAH_WMHvol	WMH in the left posterior artery hemisphere (mm^3)
- rPAH_WMHvol	WMH in the right posterior artery hemisphere (mm^3)
- lPAC_WMHvol	WMH in the left posterior artery callosal (mm^3)
- rPAC_WMHvol	WMH in the left posterior artery callosal (mm^3)

Abbreviations: WMH = white matter hyperintensity.

Table S2 List of exclusion codes and corresponding diseases from UK Biobank dataset.

Code	Disease
1081	stroke
1082	transient ischaemic attack (tia)
1083	subdural haemorrhage/haematoma
1086	subarachnoid haemorrhage
1240	neurological injury/trauma
1243	psychological/psychiatric problem
1244	infection of nervous system
1245	brain abscess/intracranial abscess
1246	encephalitis
1247	meningitis
1256	acute infective polyneuritis/guillain-barre syndrome
1258	chronic/degenerative neurological problem
1259	motor neurone disease
1261	multiple sclerosis
1262	Parkinsons disease
1263	dementia/Alzheimer's/cognitive impairment
1264	epilepsy
1266	head injury
1289	schizophrenia
1291	mania/bipolar disorder/manic depression

1297	muscle/soft tissue problem
1425	cerebral aneurysm
1433	cerebral palsy
1491	brain haemorrhage
1524	spina bifida
1583	ischaemic stroke
1659	meningioma / benign meningeal tumour

Table S3 Descriptive statistics of brain features.

Feature	All training mean ± SD	Evaluation Sample			
		Normotensive mean ± SD	Hypertensive Level 1 mean ± SD	Hypertensive Level 2 mean ± SD	Hypertensive Level 3 mean ± SD
wb_SubCortGray_Vol	55135.82 ±4638.45	54839.00 ±4685.15	55242.64 ±4796.53	55000.26 ±4839.12	54327.01 ±4759.25
wb_3rd-Ventricle_Vol	1260.13 ±464.97	1255.19 ±452.77	1410.20 ±525.53	1456.03 ±548.16	1524.14 ±569.19
wb_4th-Ventricle_Vol	1829.09 ±513.83	1809.74 ±507.68	1910.69 ±545.16	1922.72 ±549.31	1939.24 ±547.99
wb_5th-Ventricle_Vol	0.06 ±0.53	0.04 ±0.40	0.07 ±0.63	0.05 ±0.41	0.07 ±0.56
lh_Accumbens-area_Vol	421.95 ±88.71	420.72 ±89.84	410.75 ±89.74	402.38 ±88.82	387.63 ±85.64
rh_Accumbens-area_Vol	493.29 ±82.39	489.09 ±83.66	486.25 ±83.27	479.42 ±82.62	466.14 ±82.20
lh_Amygdala_Vol	1567.12 ±214.43	1561.43 ±223.42	1558.35 ±225.00	1546.45 ±225.61	1502.39 ±223.12
rh_Amygdala_Vol	1738.03 ±215.64	1730.34 ±218.25	1744.53 ±226.34	1736.69 ±229.14	1703.66 ±220.69
wb_Brain-Stem_Vol	21840.31 ±2410.48	21725.34 ±2438.12	22044.18 ±2473.96	21977.40 ±2507.31	21761.09 ±2473.32
wb_CC-Anterior_Vol	944.57 ±160.71	940.68 ±163.58	943.64 ±164.18	941.77 ±160.12	933.28 ±161.86
wb_CC-Central_Vol	541.12 ±123.47	534.69 ±127.08	525.28 ±116.47	518.74 ±115.35	503.71 ±106.03
wb_CC-Mid-Anterior_Vol	544.12 ±127.73	538.80 ±130.46	529.42 ±124.17	521.81 ±122.50	508.01 ±113.85
wb_CC-Mid-Posterior_Vol	553.49 ±105.52	545.37 ±107.04	543.70 ±105.96	537.54 ±108.28	528.01 ±109.86
wb_CC-Posterior_Vol	1045.27 ±153.16	1036.91 ±154.86	1045.95 ±155.34	1046.51 ±156.92	1045.66 ±159.90
wb_CSF_Vol	1078.88 ±243.88	1079.13 ±253.62	1130.35 ±258.85	1138.56 ±265.60	1145.07 ±285.54
lh_Caudate_Vol	3239.87 ±392.56	3214.22 ±389.31	3261.52 ±411.32	3271.66 ±433.86	3275.52 ±450.44
rh_Caudate_Vol	3356.28 ±410.29	3333.84 ±417.15	3396.86 ±432.96	3405.45 ±448.52	3416.27 ±466.61
lh_Cerebellum-Cortex_Vol	54889.54 ±5590.29	54493.94 ±5565.52	55227.01 ±5765.83	55050.78 ±5818.77	54565.72 ±5699.24
rh_Cerebellum-Cortex_Vol	56698.30 ±5976.93	56319.22 ±5934.17	57161.15 ±6179.37	56933.13 ±6246.49	56339.18 ±6056.08

lh_Cerebellum-White-Matter_Vol	16173.87 ±2047.24	16026.64 ±2078.45	16015.00 ±2125.92	15834.55 ±2137.27	15570.33 ±2105.57
rh_Cerebellum-White-Matter_Vol	15438.07 ±2133.37	15269.80 ±2103.39	15327.02 ±2240.56	15161.24 ±2189.66	14918.65 ±2312.11
lh_CerebralWhiteMatter_Vol	238150.02 ±27304.65	236148.92 ±27619.60	238863.16 ±28487.31	237606.02 ±28379.55	233977.26 ±28076.45
rh_CerebralWhiteMatter_Vol	239117.62 ±27500.37	237148.07 ±27928.71	240031.62 ±28735.33	238809.74 ±28669.08	235218.47 ±28283.37
lh_Hippocampus_Vol	3980.06 ±385.51	3954.95 ±398.77	3959.70 ±406.69	3937.34 ±409.65	3857.84 ±405.42
rh_Hippocampus_Vol	4159.30 ±408.68	4134.90 ±407.35	4133.13 ±425.35	4110.06 ±427.81	4032.22 ±414.42
lh_Inf-Lat-Vent_Vol	360.54 ±229.98	366.08 ±241.97	426.03 ±282.14	456.19 ±301.43	496.39 ±330.98
rh_Inf-Lat-Vent_Vol	363.70 ±221.89	367.88 ±223.51	415.00 ±250.34	442.57 ±278.51	479.74 ±320.62
lh_Lateral-Ventricle_Vol	11675.50 ±6548.44	11690.17 ±6605.82	13308.99 ±7274.98	13938.99 ±7610.63	14916.92 ±8054.89
rh_Lateral-Ventricle_Vol	10639.65 ±5713.65	10652.19 ±5704.89	12077.43 ±6363.45	12670.63 ±6688.88	13564.38 ±7232.57
lh_Pallidum_Vol	2014.91 ±218.47	2007.81 ±219.76	2029.22 ±229.47	2023.96 ±230.15	2004.57 ±235.44
rh_Pallidum_Vol	1974.30 ±217.89	1966.49 ±220.86	1987.47 ±228.78	1981.95 ±230.74	1968.59 ±231.14
lh_Putamen_Vol	4517.42 ±513.17	4496.29 ±510.65	4546.70 ±540.72	4526.03 ±548.86	4478.14 ±555.79
rh_Putamen_Vol	4580.66 ±514.73	4561.85 ±513.08	4611.73 ±539.66	4593.51 ±546.12	4544.49 ±543.36
lh_Thalamus-Proper_Vol	6928.82 ±722.26	6886.27 ±759.45	6888.49 ±715.92	6832.05 ±705.25	6726.25 ±685.38
rh_Thalamus-Proper_Vol	6781.15 ±675.75	6743.06 ±706.90	6774.61 ±681.19	6727.57 ±667.30	6639.39 ±670.19
lh_VentralDC_Vol	3976.10 ±400.74	3958.15 ±406.88	3979.25 ±416.70	3953.23 ±417.72	3886.43 ±404.10
rh_VentralDC_Vol	3901.07 ±389.18	3878.08 ±388.94	3901.98 ±402.81	3876.26 ±402.13	3811.35 ±393.61
wb_VentricleChoroid_Vol	23707.84 ±12648.68	23743.92 ±12695.93	27062.08 ±14107.89	28403.65 ±14813.99	30434.71 ±15856.11
lh_caudalanteriorcingulate_Thick	2.76 ±0.28	2.75 ±0.27	2.73 ±0.29	2.72 ±0.29	2.69 ±0.29
rh_caudalanteriorcingulate_Thick	2.54 ±0.33	2.54 ±0.34	2.51 ±0.34	2.50 ±0.35	2.49 ±0.35
lh_caudalmiddlefrontal_Thick	2.87 ±0.14	2.87 ±0.14	2.85 ±0.15	2.84 ±0.15	2.82 ±0.16
rh_caudalmiddlefrontal_Thick	2.83 ±0.14	2.83 ±0.14	2.81 ±0.15	2.80 ±0.15	2.79 ±0.15
lh_cuneus_Thick	2.04 ±0.14	2.04 ±0.14	2.04 ±0.14	2.04 ±0.14	2.03 ±0.15

rh_cuneus_Thick	1.95 ±0.14	1.95 ±0.14	1.95 ±0.14	1.95 ±0.14	1.95 ±0.14
lh_entorhinal_Thick	3.26 ±0.30	3.25 ±0.30	3.23 ±0.31	3.22 ±0.32	3.18 ±0.34
rh_entorhinal_Thick	3.34 ±0.33	3.34 ±0.33	3.31 ±0.34	3.30 ±0.34	3.27 ±0.35
lh_fusiform_Thick	2.89 ±0.13	2.89 ±0.13	2.87 ±0.13	2.87 ±0.13	2.85 ±0.14
rh_fusiform_Thick	2.89 ±0.14	2.88 ±0.14	2.87 ±0.15	2.86 ±0.15	2.85 ±0.15
lh_inferiorparietal_Thick	2.70 ±0.12	2.70 ±0.12	2.69 ±0.12	2.68 ±0.12	2.66 ±0.13
rh_inferiorparietal_Thick	2.73 ±0.13	2.73 ±0.13	2.71 ±0.13	2.70 ±0.14	2.68 ±0.14
lh_inferiortemporal_Thick	3.06 ±0.14	3.06 ±0.14	3.05 ±0.14	3.04 ±0.14	3.02 ±0.15
rh_inferiortemporal_Thick	3.03 ±0.14	3.02 ±0.14	3.01 ±0.14	3.01 ±0.14	2.99 ±0.14
lh_insula_Thick	3.22 ±0.16	3.21 ±0.15	3.20 ±0.17	3.19 ±0.17	3.17 ±0.18
rh_insula_Thick	3.20 ±0.16	3.20 ±0.16	3.18 ±0.17	3.17 ±0.17	3.15 ±0.17
lh_isthmuscingulate_Thick	2.48 ±0.18	2.48 ±0.18	2.46 ±0.18	2.45 ±0.18	2.45 ±0.18
rh_isthmuscingulate_Thick	2.54 ±0.19	2.54 ±0.19	2.53 ±0.19	2.52 ±0.19	2.52 ±0.20
lh_lateraloccipital_Thick	2.31 ±0.13	2.31 ±0.13	2.31 ±0.13	2.31 ±0.13	2.30 ±0.13
rh_lateraloccipital_Thick	2.34 ±0.14	2.34 ±0.14	2.34 ±0.14	2.34 ±0.14	2.33 ±0.14
lh_lateralorbitofrontal_Thick	2.84 ±0.14	2.84 ±0.14	2.83 ±0.14	2.83 ±0.14	2.81 ±0.15
rh_lateralorbitofrontal_Thick	2.83 ±0.14	2.82 ±0.14	2.82 ±0.14	2.82 ±0.15	2.81 ±0.14
lh_lingual_Thick	2.03 ±0.15	2.02 ±0.14	2.03 ±0.15	2.03 ±0.15	2.01 ±0.15
rh_lingual_Thick	2.00 ±0.15	2.00 ±0.15	2.00 ±0.15	2.00 ±0.15	2.00 ±0.15
lh_medialorbitofrontal_Thick	2.66 ±0.17	2.66 ±0.17	2.65 ±0.17	2.64 ±0.17	2.62 ±0.17
rh_medialorbitofrontal_Thick	2.69 ±0.18	2.68 ±0.18	2.67 ±0.18	2.67 ±0.18	2.65 ±0.18
lh_middletemporal_Thick	2.91 ±0.15	2.91 ±0.15	2.90 ±0.15	2.89 ±0.15	2.86 ±0.15
rh_middletemporal_Thick	2.99 ±0.14	2.99 ±0.15	2.98 ±0.15	2.97 ±0.15	2.95 ±0.14
lh_paracentral_Thick	2.72 ±0.18	2.72 ±0.18	2.69 ±0.20	2.68 ±0.20	2.64 ±0.21

rh_paracentral_Thick	2.71 ±0.19	2.70 ±0.19	2.68 ±0.19	2.66 ±0.20	2.63 ±0.21
lh_parahippocampal_Thick	2.78 ±0.29	2.79 ±0.29	2.76 ±0.30	2.76 ±0.31	2.72 ±0.31
rh_parahippocampal_Thick	2.72 ±0.25	2.72 ±0.25	2.69 ±0.26	2.69 ±0.26	2.67 ±0.26
lh_parsopercularis_Thick	2.88 ±0.14	2.87 ±0.14	2.86 ±0.15	2.85 ±0.15	2.84 ±0.15
rh_parsopercularis_Thick	2.83 ±0.14	2.83 ±0.14	2.82 ±0.14	2.81 ±0.14	2.79 ±0.14
lh_parsorbitalis_Thick	2.89 ±0.17	2.88 ±0.16	2.87 ±0.17	2.86 ±0.17	2.86 ±0.18
rh_parsorbitalis_Thick	2.90 ±0.18	2.89 ±0.18	2.87 ±0.18	2.86 ±0.18	2.84 ±0.19
lh_parstriangularis_Thick	2.71 ±0.14	2.71 ±0.14	2.69 ±0.14	2.68 ±0.14	2.67 ±0.14
rh_parstriangularis_Thick	2.69 ±0.13	2.68 ±0.13	2.67 ±0.14	2.66 ±0.14	2.65 ±0.14
lh_pericalcarine_Thick	1.73 ±0.14	1.74 ±0.14	1.74 ±0.14	1.74 ±0.14	1.73 ±0.14
rh_pericalcarine_Thick	1.70 ±0.14	1.71 ±0.15	1.71 ±0.14	1.71 ±0.14	1.71 ±0.14
lh_postcentral_Thick	2.35 ±0.14	2.35 ±0.14	2.34 ±0.14	2.33 ±0.14	2.32 ±0.15
rh_postcentral_Thick	2.31 ±0.15	2.30 ±0.15	2.29 ±0.15	2.28 ±0.15	2.27 ±0.15
lh_posteriorcingulate_Thick	2.70 ±0.17	2.70 ±0.17	2.68 ±0.17	2.67 ±0.18	2.66 ±0.18
rh_posteriorcingulate_Thick	2.70 ±0.17	2.71 ±0.17	2.69 ±0.18	2.67 ±0.19	2.66 ±0.18
lh_precentral_Thick	2.85 ±0.17	2.84 ±0.17	2.82 ±0.18	2.81 ±0.18	2.78 ±0.19
rh_precentral_Thick	2.80 ±0.18	2.79 ±0.18	2.77 ±0.19	2.75 ±0.19	2.73 ±0.19
lh_precuneus_Thick	2.63 ±0.14	2.63 ±0.14	2.62 ±0.14	2.61 ±0.14	2.59 ±0.15
rh_precuneus_Thick	2.63 ±0.13	2.62 ±0.13	2.61 ±0.14	2.60 ±0.14	2.59 ±0.14
lh_rostralanteriorcingulate_Thick	2.91 ±0.19	2.91 ±0.18	2.89 ±0.19	2.89 ±0.19	2.87 ±0.19
rh_rostralanteriorcingulate_Thick	2.95 ±0.20	2.95 ±0.19	2.94 ±0.20	2.94 ±0.21	2.92 ±0.20
lh_rostralmiddlefrontal_Thick	2.69 ±0.13	2.68 ±0.13	2.67 ±0.13	2.66 ±0.13	2.64 ±0.14
rh_rostralmiddlefrontal_Thick	2.63 ±0.12	2.63 ±0.12	2.62 ±0.12	2.61 ±0.12	2.60 ±0.12
lh_superiorfrontal_Thick	2.96 ±0.14	2.96 ±0.14	2.94 ±0.15	2.93 ±0.15	2.90 ±0.15

rh_superiorfrontal_Thick	2.92 ±0.13	2.91 ±0.13	2.90 ±0.14	2.89 ±0.14	2.86 ±0.14
lh_superiorparietal_Thick	2.48 ±0.13	2.48 ±0.13	2.47 ±0.14	2.46 ±0.14	2.45 ±0.15
rh_superiorparietal_Thick	2.45 ±0.14	2.45 ±0.14	2.44 ±0.14	2.43 ±0.14	2.42 ±0.14
lh_superiortemporal_Thick	3.00 ±0.16	2.99 ±0.16	2.97 ±0.17	2.96 ±0.17	2.93 ±0.17
rh_superiortemporal_Thick	3.07 ±0.16	3.06 ±0.16	3.04 ±0.16	3.03 ±0.17	3.00 ±0.16
lh_supramarginal_Thick	2.83 ±0.14	2.83 ±0.13	2.81 ±0.14	2.80 ±0.14	2.78 ±0.15
rh_supramarginal_Thick	2.83 ±0.14	2.83 ±0.14	2.81 ±0.14	2.80 ±0.15	2.78 ±0.15
lh_transversetemporal_Thick	2.57 ±0.25	2.56 ±0.25	2.55 ±0.25	2.55 ±0.25	2.55 ±0.24
rh_transversetemporal_Thick	2.61 ±0.26	2.60 ±0.26	2.59 ±0.26	2.60 ±0.26	2.59 ±0.27
wb_TotalGray_Vol	667395.55 ±57463.30	662913.38 ±56681.81	667659.36 ±59019.74	664256.84 ±59134.05	653821.87 ±57330.17
lh_Cortex_Vol	248706.68 ±23543.49	247083.66 ±23260.72	248369.75 ±24143.64	247020.09 ±24094.64	242456.78 ±23605.19
rh_Cortex_Vol	249263.54 ±23555.54	247508.60 ±23312.33	248976.86 ±24171.64	247567.46 ±24250.98	243460.57 ±23720.65
lh_caudalanteriorcingulate_Vol	3130.95 ±568.30	3108.44 ±572.52	3115.35 ±577.88	3096.20 ±574.67	3024.54 ±564.81
rh_caudalanteriorcingulate_Vol	2262.61 ±563.69	2256.00 ±566.97	2243.23 ±575.00	2214.15 ±568.68	2175.34 ±578.87
lh_caudalmiddlefrontal_Vol	7166.93 ±1119.94	7114.61 ±1073.72	7139.24 ±1150.99	7086.78 ±1128.47	6945.31 ±1086.27
rh_caudalmiddlefrontal_Vol	6648.15 ±1107.74	6566.10 ±1075.43	6600.57 ±1100.97	6566.55 ±1095.44	6452.75 ±1075.88
lh_cuneus_Vol	4363.36 ±713.35	4355.20 ±704.40	4372.80 ±726.58	4383.68 ±733.98	4336.58 ±758.43
rh_cuneus_Vol	3946.93 ±633.23	3925.28 ±608.67	3977.18 ±648.35	3988.00 ±665.26	3936.03 ±672.58
lh_entorhinal_Vol	1773.65 ±354.73	1768.80 ±342.26	1792.22 ±363.36	1786.49 ±363.42	1762.94 ±360.79
rh_entorhinal_Vol	1701.65 ±332.64	1698.16 ±323.29	1711.13 ±338.85	1712.17 ±346.73	1690.81 ±341.04
lh_fusiform_Vol	9174.42 ±1191.18	9105.86 ±1173.22	9161.23 ±1230.19	9097.37 ±1225.05	8951.59 ±1225.11
rh_fusiform_Vol	8934.51 ±1248.66	8861.84 ±1217.06	8968.31 ±1272.43	8896.57 ±1270.85	8755.71 ±1250.41
lh_inferiorparietal_Vol	12740.32 ±1803.80	12677.49 ±1808.78	12663.65 ±1833.23	12571.89 ±1807.62	12302.22 ±1732.78
rh_inferiorparietal_Vol	15146.79 ±2116.58	15030.10 ±2138.37	15119.76 ±2151.61	15009.03 ±2163.56	14719.75 ±2088.85

lh_inferiortemporal_Vol	12600.16 ±1659.49	12514.13 ±1652.90	12605.43 ±1661.56	12512.15 ±1677.33	12297.33 ±1640.30
rh_inferiortemporal_Vol	12746.61 ±1681.69	12640.77 ±1683.97	12781.11 ±1708.68	12707.08 ±1707.20	12420.58 ±1654.00
lh_insula_Vol	6360.43 ±667.33	6321.79 ±660.06	6418.84 ±687.04	6404.54 ±676.58	6354.21 ±661.19
rh_insula_Vol	6574.55 ±690.65	6532.45 ±679.60	6632.51 ±703.45	6616.66 ±701.91	6573.45 ±681.71
lh_isthmuscingulate_Vol	2708.25 ±434.69	2694.35 ±436.18	2738.55 ±445.00	2730.72 ±441.47	2696.06 ±436.86
rh_isthmuscingulate_Vol	2568.45 ±406.07	2560.81 ±421.50	2590.98 ±414.42	2588.51 ±414.62	2554.45 ±413.75
lh_lateraloccipital_Vol	12507.20 ±1735.68	12479.05 ±1686.42	12563.42 ±1762.92	12554.24 ±1800.11	12334.05 ±1739.29
rh_lateraloccipital_Vol	12797.03 ±1828.21	12752.02 ±1824.70	12898.30 ±1862.45	12853.43 ±1909.38	12687.62 ±1878.58
lh_lateralorbitofrontal_Vol	9258.51 ±1000.05	9220.02 ±994.72	9250.06 ±1018.60	9206.93 ±1002.79	9067.88 ±984.68
rh_lateralorbitofrontal_Vol	9252.73 ±979.75	9191.84 ±963.96	9234.14 ±995.72	9178.24 ±983.55	9047.28 ±947.01
lh_lingual_Vol	6597.68 ±1152.06	6560.39 ±1087.87	6620.11 ±1160.73	6605.54 ±1128.39	6463.89 ±1146.30
rh_lingual_Vol	6617.04 ±1137.62	6605.01 ±1107.88	6641.40 ±1154.28	6645.27 ±1139.08	6567.89 ±1168.45
lh_medialorbitofrontal_Vol	5059.30 ±596.27	5054.86 ±604.43	5057.20 ±601.98	5023.88 ±594.88	4950.90 ±595.77
rh_medialorbitofrontal_Vol	4939.17 ±562.69	4918.79 ±564.61	4917.48 ±567.63	4888.10 ±561.63	4818.64 ±543.32
lh_middletemporal_Vol	14194.22 ±1899.32	14101.10 ±1937.79	14172.20 ±1948.68	14047.24 ±1926.82	13669.18 ±1893.07
rh_middletemporal_Vol	14395.21 ±1762.27	14281.25 ±1814.18	14364.02 ±1826.31	14250.33 ±1793.10	13957.04 ±1785.98
lh_paracentral_Vol	4693.07 ±605.70	4660.87 ±590.66	4640.41 ±608.25	4619.57 ±607.00	4539.40 ±607.74
rh_paracentral_Vol	4529.09 ±615.70	4500.58 ±613.65	4492.71 ±623.56	4476.95 ±620.71	4389.51 ±610.45
lh_parahippocampal_Vol	2172.67 ±318.20	2170.55 ±313.38	2151.26 ±324.03	2141.47 ±324.80	2103.65 ±321.02
rh_parahippocampal_Vol	2067.29 ±277.39	2063.58 ±281.04	2043.54 ±276.72	2036.49 ±280.19	2010.71 ±277.58
lh_parsopercularis_Vol	4605.68 ±720.59	4559.24 ±703.33	4573.63 ±727.86	4535.18 ±715.67	4444.99 ±693.57
rh_parsopercularis_Vol	4710.19 ±732.53	4667.69 ±719.91	4693.69 ±735.18	4647.89 ±722.34	4564.67 ±710.18
lh_parsorbitalis_Vol	2288.59 ±312.36	2264.17 ±311.66	2282.02 ±316.19	2260.41 ±314.33	2236.87 ±321.45
rh_parsorbitalis_Vol	2355.17 ±355.90	2338.04 ±358.39	2344.10 ±356.01	2321.11 ±353.59	2265.84 ±336.75

lh_parstriangularis_Vol	4564.67 ±756.40	4518.37 ±736.42	4546.78 ±771.39	4517.50 ±751.14	4390.88 ±719.71
rh_parstriangularis_Vol	4203.05 ±706.82	4182.55 ±694.15	4197.54 ±728.57	4162.36 ±705.16	4093.14 ±682.40
lh_pericalcarine_Vol	2110.46 ±436.43	2107.76 ±419.82	2129.90 ±441.77	2143.43 ±444.38	2114.85 ±440.42
rh_pericalcarine_Vol	2301.13 ±462.23	2297.99 ±451.41	2330.46 ±469.94	2341.22 ±471.40	2324.51 ±480.08
lh_postcentral_Vol	11755.45 ±1474.70	11677.28 ±1451.46	11732.16 ±1511.99	11671.63 ±1497.59	11483.08 ±1461.86
rh_postcentral_Vol	10954.24 ±1459.62	10916.87 ±1473.99	10909.42 ±1451.17	10866.89 ±1460.57	10707.44 ±1432.75
lh_posteriorcingulate_Vol	3615.56 ±511.85	3594.94 ±524.31	3618.94 ±537.64	3605.43 ±532.83	3543.56 ±500.37
rh_posteriorcingulate_Vol	3650.89 ±525.90	3621.46 ±540.95	3636.84 ±557.52	3614.68 ±552.33	3554.50 ±539.79
lh_precentral_Vol	14168.44 ±1633.66	14085.29 ±1595.21	14111.92 ±1679.34	14020.22 ±1650.08	13759.86 ±1637.76
rh_precentral_Vol	13639.25 ±1613.54	13533.01 ±1567.60	13562.94 ±1655.24	13477.91 ±1644.33	13238.50 ±1602.54
lh_precuneus_Vol	10439.52 ±1317.72	10348.54 ±1302.89	10399.88 ±1336.40	10328.39 ±1345.51	10136.50 ±1319.12
rh_precuneus_Vol	11167.02 ±1371.39	11088.23 ±1408.68	11145.50 ±1393.20	11075.79 ±1414.90	10893.85 ±1389.09
lh_rostralanteriorcingulate_Vol	3759.16 ±635.37	3741.35 ±640.16	3762.90 ±638.18	3729.15 ±622.14	3680.39 ±609.27
rh_rostralanteriorcingulate_Vol	2678.28 ±528.09	2669.36 ±531.56	2680.43 ±532.52	2661.83 ±530.66	2631.49 ±530.68
lh_rostralmiddlefrontal_Vol	11706.22 ±1702.80	11589.88 ±1685.94	11698.51 ±1736.04	11626.44 ±1732.06	11388.70 ±1710.57
rh_rostralmiddlefrontal_Vol	11820.03 ±1802.57	11720.75 ±1768.88	11789.30 ±1810.25	11696.07 ±1806.23	11461.38 ±1721.58
lh_superiorfrontal_Vol	25641.11 ±2920.07	25467.05 ±2848.80	25576.70 ±3007.57	25437.31 ±2965.24	24947.84 ±2924.04
rh_superiorfrontal_Vol	28491.57 ±3327.54	28219.51 ±3227.77	28457.02 ±3438.14	28312.78 ±3432.93	27759.10 ±3341.10
lh_superiorparietal_Vol	11560.10 ±1589.61	11425.05 ±1556.54	11495.48 ±1587.00	11431.95 ±1585.02	11242.35 ±1558.26
rh_superiorparietal_Vol	11694.81 ±1594.61	11593.19 ±1591.61	11634.82 ±1588.06	11524.25 ±1585.73	11427.86 ±1536.89
lh_superiortemporal_Vol	17378.23 ±1968.01	17299.47 ±1985.35	17378.56 ±2014.83	17313.05 ±2020.94	16991.76 ±1963.79
rh_superiortemporal_Vol	16502.49 ±1722.87	16419.23 ±1725.94	16459.65 ±1774.84	16375.25 ±1767.72	16093.63 ±1720.16
lh_supramarginal_Vol	10986.84 ±1695.10	10869.79 ±1745.84	11005.83 ±1718.25	10936.13 ±1693.00	10714.11 ±1663.42
rh_supramarginal_Vol	10372.28 ±1453.02	10269.69 ±1417.88	10346.83 ±1477.07	10295.05 ±1453.23	10091.46 ±1400.61

lh_transversetemporal_Vol	1251.31 ±224.93	1233.90 ±220.25	1246.23 ±229.22	1241.48 ±227.75	1225.08 ±221.02
rh_transversetemporal_Vol	991.32 ±170.17	979.17 ±161.76	987.65 ±171.96	986.52 ±170.72	972.31 ±167.85
wholeBrainWMHvol	1768.09 ±2781.08	1736.62 ±2500.84	2433.40 ±3315.97	2826.63 ±3763.55	3933.86 ±5122.58
PVWMHvol	1306.82 ±1848.85	1280.22 ±1645.52	1797.52 ±2373.29	2099.64 ±2755.11	2918.03 ±3608.72
DWMHvol	438.01 ±1042.36	433.55 ±1092.36	608.44 ±1140.08	697.37 ±1226.06	980.13 ±1929.39
Lfrontal_WMHvol	86.19 ±213.10	86.12 ±306.76	120.00 ±263.27	132.58 ±269.40	174.69 ±351.04
Rfrontal_WMHvol	90.87 ±220.14	91.36 ±284.00	127.78 ±298.69	141.77 ±270.57	187.73 ±369.43
Ltemporal_WMHvol	13.17 ±67.81	10.47 ±26.07	17.17 ±54.60	19.52 ±51.12	28.83 ±80.48
Rtemporal_WMHvol	12.18 ±100.13	9.69 ±29.23	14.68 ±43.10	17.96 ±82.08	25.05 ±62.13
Lparietal_WMHvol	82.48 ±268.78	82.78 ±275.56	121.44 ±309.61	142.83 ±376.07	216.93 ±577.52
Rparietal_WMHvol	85.13 ±278.81	81.78 ±252.76	124.34 ±302.58	148.21 ±331.60	232.31 ±628.92
Loccipital_WMHvol	27.09 ±46.38	28.68 ±43.74	33.48 ±55.43	38.33 ±64.68	46.90 ±75.79
Roccipital_WMHvol	40.90 ±61.61	42.67 ±60.77	49.53 ±71.76	56.16 ±81.59	67.68 ±99.88
Lcerebellum_WMHvol	0.38 ±5.68	0.23 ±1.32	0.48 ±10.46	0.46 ±4.52	0.42 ±4.50
Rcerebellum_WMHvol	0.30 ±3.91	0.25 ±1.37	0.35 ±3.30	0.41 ±6.04	0.35 ±3.42
Brainstem_WMHvol	10.80 ±51.90	10.77 ±23.33	13.26 ±33.23	14.59 ±36.23	17.50 ±46.67
lAAH_WMHvol	55.13 ±127.45	56.26 ±128.70	76.02 ±133.11	85.49 ±141.58	119.27 ±196.62
rAAH_WMHvol	51.77 ±141.89	50.99 ±119.38	72.51 ±161.27	83.76 ±167.70	110.29 ±194.57
lMAH_WMHvol	246.81 ±655.23	242.81 ±667.33	368.39 ±794.88	429.90 ±888.72	627.57 ±1244.33
rMAH_WMHvol	276.33 ±649.41	267.17 ±623.75	389.41 ±710.57	457.38 ±803.76	655.03 ±1126.29
lAAML_WMHvol	92.90 ±105.42	90.39 ±97.56	117.37 ±133.18	135.59 ±152.80	177.49 ±196.81
rAAML_WMHvol	95.32 ±103.89	93.96 ±103.05	119.87 ±131.67	136.80 ±152.67	176.91 ±191.24
lAAC_WMHvol	103.17 ±131.64	100.14 ±122.44	135.53 ±170.67	157.24 ±191.27	210.08 ±233.72
rAAC_WMHvol	92.39 ±114.56	91.16 ±105.54	117.62 ±145.95	131.62 ±161.35	170.03 ±190.67

lMALL_WMHvol	250.85 ±371.26	250.47 ±362.30	363.94 ±500.55	429.11 ±570.62	599.49 ±711.94
rMALL_WMHvol	221.46 ±330.15	217.31 ±314.86	321.28 ±446.68	376.84 ±507.64	526.30 ±632.23
lPATMP_WMHvol	60.44 ±51.49	59.04 ±36.43	59.75 ±39.53	61.23 ±42.60	64.20 ±48.73
rPATMP_WMHvol	42.65 ±44.53	41.63 ±28.02	43.82 ±33.49	45.52 ±37.60	55.66 ±181.31
lPAH_WMHvol	67.64 ±162.01	67.10 ±117.13	93.41 ±195.78	111.97 ±233.16	169.13 ±361.95
rPAH_WMHvol	94.41 ±218.80	91.63 ±163.61	130.50 ±256.44	156.03 ±294.98	230.98 ±476.39
lPAC_WMHvol	3.49 ±20.10	3.30 ±19.56	6.45 ±31.61	8.41 ±37.31	15.56 ±52.39
rPAC_WMHvol	1.36 ±10.77	1.40 ±10.09	2.70 ±16.86	3.39 ±19.54	6.41 ±27.78

Abbreviations: lh = left hemisphere; rh = right hemisphere; wb = whole-brain; Vol = volume; Thick = mean thickness; WMH = white matter hyperintensity; SD = standard deviation. See Table S1 WMH-related abbreviations.

Table S4 Correlations between covariates and selected features using Spearman and point-biserial tests in the training dataset.

	Spearman Correlation Test				Point-Biserial Correlation Test									
	Age		Intracranial Volume		Sex		Diabetes		Hypercholestero-lemia		Obesity		Smoking	
	r_s	p-value	r_s	p-value	r_{pb}	p-value	r_{pb}	p-value	r_{pb}	p-value	r_{pb}	p-value	r_{pb}	p-value
Mean thickness of the left insula	-0.087	**<0.001**	0.090	**<0.001**	0.113	**<0.001**	-0.028	0.393	-0.029	0.396	-0.014	0.952	-0.022	0.841
Mean thickness of the right insula	-0.134	**<0.001**	0.109	**<0.001**	0.081	**0.001**	-0.051	0.164	-0.092	**0.001**	-0.028	0.759	-0.037	0.791
Volume of whole-brain subcortical grey matter volume	-0.285	**<0.001**	0.771	**<0.001**	0.471	**<0.001**	-0.035	0.324	-0.033	0.303	0.024	0.822	-0.015	0.886
Volume of the 5th ventricle	-0.028	0.258	0.010	0.702	-0.003	0.915	0.017	0.600	-0.041	0.194	-0.008	0.952	0.017	0.868
Volume of whole-brain cortical grey matter	-0.218	**<0.001**	0.853	**<0.001**	0.545	**<0.001**	-0.033	0.338	-0.021	0.571	0.008	0.952	-0.020	0.854
Volume of whole-brain WMH	0.394	**<0.001**	0.086	**<0.001**	-0.009	0.755	0.081	**0.017**	0.118	**<0.001**	0.018	0.909	0.056	0.355
WMH volume of the left cerebellum	-0.052	**0.037**	0.037	0.153	0.030	0.260	-0.025	0.460	0.004	0.936	-0.006	0.952	-0.013	0.890
WMH volume of the right cerebellum	-0.038	0.128	0.035	0.168	0.037	0.156	-0.020	0.548	-0.033	0.303	0.020	0.858	-2.224	0.999
WMH volume of the left posterior artery callosal	0.218	**<0.001**	0.019	0.468	0.029	0.267	0.095	**0.002**	0.047	0.126	0.001	0.980	0.012	0.893
WMH volume of the right posterior artery callosal	0.170	**<0.001**	0.019	0.469	0.002	0.931	0.070	0.056	0.075	**0.010**	-0.048	0.586	0.009	0.907

This table displays Spearman correlation coefficients (r_s) and Point-Biserial correlation coefficients (r_{pb}) and corresponding corrected p-values for associations between various covariates and selected brain measures in the training dataset. The Spearman correlation is used for assessing relationships between continuous variables, while the point-biserial correlation is used to assess relationships between binary variables and brain measures. P-values were corrected for multiple comparisons using the False Discovery Rate (FDR) method. Statistically significant correlations (p < 0.05) are highlighted. Abbreviations: WMH = white matter hyperintensity.

Table S5 Median predictions of selected brain measures before and after age perturbation on the hold-out dataset.

	**Prior Sampling**			**Posterior Sampling**		
	Original	Perturbed	Median Shift†	Original	Perturbed	Median Shift†
Mean thickness of the left insula	-0.020	-0.076	-0.056	0.005	0.013	0.008
Mean thickness of the right insula	-0.042	-0.152	-0.110	0.020	0.031	0.011
Volume of whole-brain subcortical grey matter volume	-0.085	-0.305	-0.220	-0.160	-0.153	0.007
Volume of whole-brain cortical grey matter	-0.143	-0.307	-0.164	-0.167	-0.168	-0.001
Volume of whole-brain WMH	-0.019	0.311	0.330	-0.098	-0.092	0.006

This table presents the sensitivity analysis results comparing median predictions (normalised) of selected brain measures when age is perturbed by one standard deviation. Original and Perturbed columns show median predictions before and after age perturbation, respectively. †Median shift represents the difference between median of predictions from perturbed and original age values (perturbed - original), where positive values indicate increased predictions after age perturbation. A larger absolute shift indicates greater sensitivity to age perturbation. Abbreviations: WMH = white matter hyperintensity.

Table S6 Summary of model performance metrics across brain regions and performance metrics.

	MedianAE					RMSE					Spearman's Correlation				
	Prior-cVAE	Post-cVAE	GAMLSS	MFPR	HBR	Prior-cVAE	Post-cVAE	GAMLSS	MFPR	HBR	Prior-cVAE	Post-cVAE	GAMLSS	MFPR	HBR
wb_SubCortGray_Vol	0.39	0.04	0.38	0.38	0.38	0.59	0.07	0.59	0.59	0.59	0.81**	1**	0.82**	0.82**	0.82**
wb_3rd-Ventricle_Vol	0.51	0.31	0.49	0.50	0.50	0.79	0.47	0.82	0.78	0.78	0.60**	0.87**	0.61**	0.61**	0.61**
wb_4th-Ventricle_Vol	0.62	0.53	0.61	0.62	0.63	0.92	0.79	0.93	0.92	0.92	0.34**	0.57**	0.35**	0.35**	0.35**
wb_5th-Ventricle_Vol	0	0	0	0.14	0	0.91	0.91	0.91	0.90	0.91	-0.02	0.19**	0.01	0	0.01
lh_Accumbens-area_Vol	0.56	0.38	0.56	0.57	0.57	0.85	0.55	0.87	0.85	0.85	0.53**	0.83**	0.54**	0.54**	0.53
rh_Accumbens-area_Vol	0.57	0.35	0.56	0.56	0.56	0.87	0.54	0.88	0.87	0.87	0.51**	0.84**	0.52**	0.52**	0.52**
lh_Amygdala_Vol	0.54	0.35	0.53	0.53	0.54	0.83	0.54	0.84	0.82	0.83	0.59**	0.85**	0.60**	0.60**	0.60**
rh_Amygdala_Vol	0.51	0.33	0.52	0.51	0.51	0.78	0.50	0.78	0.77	0.77	0.63**	0.86**	0.64**	0.64**	0.64**
wb_Brain-Stem_Vol	0.47	0.25	0.47	0.47	0.47	0.72	0.37	0.73	0.72	0.72	0.69**	0.92**	0.70**	0.70**	0.69**
wb_CC-Anterior_Vol	0.57	0.37	0.57	0.57	0.57	0.89	0.60	0.91	0.89	0.89	0.48**	0.81**	0.48**	0.48**	0.48**
wb_CC-Central_Vol	0.56	0.27	0.48	0.59	0.62	0.98	0.49	1.12	0.98	0.98	0.34**	0.88**	0.35**	0.34**	0.34**
wb_CC-Mid-Anterior_Vol	0.58	0.29	0.52	0.59	0.61	0.96	0.53	1.11	0.96	0.96	0.39**	0.86**	0.39**	0.39**	0.39**
wb_CC-Mid-Posterior_Vol	0.65	0.36	0.64	0.64	0.65	0.95	0.57	0.95	0.95	0.95	0.34**	0.82**	0.34**	0.35**	0.34**
wb_CC-Posterior_Vol	0.59	0.35	0.59	0.59	0.60	0.93	0.57	0.93	0.93	0.93	0.39**	0.82**	0.38**	0.38**	0.39**
wb_CSF_Vol	0.52	0.41	0.52	0.52	0.52	0.88	0.70	0.96	0.87	0.88	0.55**	0.75**	0.55**	0.55**	0.55**
lh_Caudate_Vol	0.57	0.14	0.57	0.57	0.57	0.86	0.23	0.87	0.86	0.86	0.49**	0.97**	0.49**	0.49**	0.49**
rh_Caudate_Vol	0.58	0.14	0.56	0.58	0.58	0.87	0.24	0.88	0.86	0.86	0.52**	0.97**	0.52**	0.52**	0.52**
lh_Cerebellum-Cortex_Vol	0.54	0.13	0.54	0.54	0.54	0.80	0.21	0.80	0.80	0.80	0.58**	0.98**	0.59**	0.59**	0.59**
rh_Cerebellum-Cortex_Vol	0.52	0.12	0.51	0.52	0.53	0.78	0.19	0.78	0.78	0.78	0.61**	0.98**	0.62**	0.62**	0.62**
lh_Cerebellum-White-Matter_Vol	0.55	0.19	0.55	0.53	0.54	0.82	0.33	0.83	0.82	0.82	0.58**	0.95**	0.59**	0.59**	0.59**
rh_Cerebellum-White-Matter_Vol	0.49	0.17	0.48	0.49	0.50	0.83	0.38	0.85	0.83	0.83	0.57**	0.95**	0.58**	0.58**	0.58**
lh_CerebralWhiteMatter_Vol	0.33	0.08	0.32	0.32	0.32	0.50	0.12	0.50	0.50	0.50	0.86**	0.99**	0.86**	0.86**	0.86**
rh_CerebralWhiteMatter_Vol	0.33	0.08	0.32	0.32	0.32	0.51	0.12	0.50	0.50	0.50	0.86**	0.99**	0.86**	0.86**	0.86**
lh_Hippocampus_Vol	0.55	0.22	0.54	0.53	0.53	0.82	0.35	0.81	0.81	0.81	0.60**	0.93**	0.61**	0.61**	0.62**

rh_Hippocampus_Vol	0.55	0.20	0.53	0.54	0.54	0.79	0.32	0.79	0.79	0.79	0.59**	0.94**	0.60**	0.60**	0.61**
lh_Inf-Lat-Vent_Vol	0.58	0.42	0.57	0.58	0.57	0.88	0.64	0.87	0.87	0.87	0.47**	0.75**	0.47**	0.47**	0.47**
rh_Inf-Lat-Vent_Vol	0.63	0.47	0.62	0.62	0.63	0.93	0.74	0.93	0.93	0.93	0.39**	0.66**	0.39**	0.39**	0.38**
lh_Lateral-Ventricle_Vol	0.57	0.10	0.55	0.56	0.56	0.84	0.16	0.84	0.84	0.84	0.53**	0.99**	0.53**	0.53**	0.53**
rh_Lateral-Ventricle_Vol	0.56	0.12	0.55	0.54	0.54	0.84	0.18	0.84	0.84	0.84	0.53**	0.98**	0.53**	0.53**	0.54**
lh_Pallidum_Vol	0.51	0.28	0.49	0.51	0.51	0.78	0.44	0.78	0.77	0.77	0.63**	0.89**	0.64**	0.64**	0.64**
rh_Pallidum_Vol	0.52	0.28	0.51	0.50	0.50	0.78	0.43	0.79	0.78	0.78	0.63**	0.9**	0.64**	0.64**	0.64**
lh_Putamen_Vol	0.51	0.14	0.51	0.51	0.51	0.78	0.22	0.78	0.78	0.78	0.61**	0.97**	0.61**	0.61**	0.62**
rh_Putamen_Vol	0.53	0.14	0.53	0.54	0.53	0.81	0.24	0.81	0.81	0.81	0.59**	0.97**	0.59**	0.59**	0.59**
lh_Thalamus-Proper_Vol	0.45	0.16	0.45	0.46	0.45	0.73	0.28	0.75	0.73	0.73	0.74**	0.97**	0.74**	0.74**	0.74**
rh_Thalamus-Proper_Vol	0.44	0.17	0.44	0.44	0.43	0.71	0.29	0.72	0.71	0.71	0.75**	0.96**	0.75**	0.75**	0.75**
lh_VentralDC_Vol	0.46	0.17	0.45	0.46	0.46	0.70	0.27	0.71	0.70	0.70	0.72**	0.96**	0.72**	0.72**	0.72**
rh_VentralDC_Vol	0.47	0.17	0.47	0.47	0.47	0.69	0.26	0.70	0.69	0.69	0.71**	0.96**	0.72**	0.72**	0.72**
wb_VentricleChoroid_Vol	0.55	0.05	0.54	0.55	0.54	0.83	0.08	0.83	0.83	0.83	0.55**	1**	0.55**	0.55**	0.55**
lh_caudalanteriorcingulate_Thick	0.64	0.37	0.64	0.64	0.64	0.98	0.57	1.06	0.98	0.99	0.14**	0.80**	0.14**	0.14**	0.13**
rh_caudalanteriorcingulate_Thick	0.61	0.37	0.61	0.61	0.61	1	0.59	1.03	1	1	0.17**	0.80**	0.16**	0.16**	0.16**
lh_caudalmiddlefrontal_Thick	0.64	0.34	0.64	0.64	0.64	0.96	0.51	0.96	0.96	0.96	0.26**	0.84**	0.26**	0.26**	0.26**
rh_caudalmiddlefrontal_Thick	0.63	0.34	0.63	0.62	0.62	0.96	0.5	0.96	0.95	0.95	0.26**	0.85**	0.27**	0.27**	0.27**
lh_cuneus_Thick	0.67	0.28	0.65	0.66	0.66	0.98	0.43	0.98	0.98	0.98	0.12**	0.89**	0.11**	0.11**	0.11**
rh_cuneus_Thick	0.68	0.31	0.68	0.68	0.67	1.02	0.44	1.02	1.02	1.02	0.09**	0.90**	0.09**	0.09**	0.08**
lh_entorhinal_Thick	0.65	0.44	0.62	0.63	0.64	1.01	0.7	1.02	1.01	1.01	0.12**	0.72**	0.13**	0.14**	0.12**
rh_entorhinal_Thick	0.63	0.42	0.63	0.63	0.64	1	0.67	1	0.99	0.99	0.12**	0.74**	0.13**	0.12**	0.10**
lh_fusiform_Thick	0.65	0.39	0.67	0.67	0.66	1	0.59	1	1	1	0.17**	0.79**	0.16**	0.16**	0.15**
rh_fusiform_Thick	0.65	0.39	0.66	0.64	0.66	0.97	0.56	0.98	0.97	0.97	0.20**	0.81**	0.20**	0.21**	0.21**
lh_inferiorparietal_Thick	0.63	0.31	0.63	0.63	0.63	0.96	0.47	0.96	0.96	0.96	0.28**	0.87**	0.28**	0.28**	0.27**
rh_inferiorparietal_Thick	0.64	0.30	0.64	0.64	0.64	0.95	0.45	0.95	0.95	0.95	0.30**	0.89**	0.30**	0.30**	0.31**
lh_inferiortemporal_Thick	0.66	0.39	0.66	0.66	0.67	1	0.58	1	1	1	0.15**	0.81**	0.16**	0.16**	0.15**
rh_inferiortemporal_Thick	0.65	0.39	0.67	0.66	0.66	0.99	0.57	0.99	0.99	0.98	0.15**	0.80**	0.15**	0.15**	0.15**
lh_insula_Thick	0.64	0.40	0.65	0.63	0.63	0.97	0.61	0.98	0.97	0.97	0.14**	0.77**	0.13**	0.13**	0.13**

rh_insula_Thick	0.65	0.42	0.65	0.65	0.64	0.98	0.62	0.98	0.98	0.98	0.18**	0.78**	0.18**	0.18**	0.18**
lh_isthmuscingulate_Thick	0.66	0.45	0.66	0.67	0.67	1	0.69	1.03	1	1	0.18**	0.74**	0.17**	0.17**	0.16**
rh_isthmuscingulate_Thick	0.67	0.43	0.66	0.66	0.67	1.01	0.65	1.03	1.01	1.01	0.19**	0.79**	0.19**	0.19**	0.18**
lh_lateraloccipital_Thick	0.65	0.29	0.66	0.65	0.65	1	0.43	1	1	1	0.15**	0.90**	0.15**	0.15**	0.15**
rh_lateraloccipital_Thick	0.64	0.27	0.64	0.64	0.65	0.98	0.43	0.98	0.98	0.98	0.16**	0.90**	0.17**	0.17**	0.16**
lh_lateralorbitofrontal_Thick	0.68	0.41	0.67	0.68	0.69	1	0.64	1.01	1	1.01	0.09**	0.77**	0.10**	0.09**	0.04**
rh_lateralorbitofrontal_Thick	0.67	0.47	0.67	0.67	0.66	0.99	0.66	0.99	0.99	0.99	0.08**	0.73**	0.08**	0.08**	0.08**
lh_lingual_Thick	0.65	0.26	0.65	0.65	0.65	0.97	0.39	0.97	0.97	0.97	0.14**	0.92**	0.15**	0.15**	0.14**
rh_lingual_Thick	0.66	0.28	0.66	0.66	0.65	1.02	0.41	1.01	1.01	1.01	0.08**	0.91**	0.09**	0.09**	0.08**
lh_medialorbitofrontal_Thick	0.66	0.45	0.65	0.66	0.66	1.01	0.67	1.01	1.01	1.01	0.13**	0.74**	0.13**	0.13**	0.14**
rh_medialorbitofrontal_Thick	0.66	0.45	0.65	0.66	0.66	1.02	0.68	1.02	1.01	1.01	0.07**	0.72**	0.08**	0.08**	0.08**
lh_middletemporal_Thick	0.65	0.32	0.67	0.65	0.66	1	0.5	1.02	0.99	1	0.19**	0.86**	0.19**	0.19**	0.18**
rh_middletemporal_Thick	0.66	0.34	0.65	0.65	0.65	1.01	0.52	1.01	1.01	1.01	0.21**	0.86**	0.21**	0.21**	0.21**
lh_paracentral_Thick	0.64	0.29	0.65	0.64	0.65	0.96	0.44	0.98	0.96	0.96	0.24**	0.89**	0.24**	0.24**	0.24**
rh_paracentral_Thick	0.64	0.29	0.66	0.64	0.65	0.97	0.47	0.98	0.97	0.97	0.25**	0.88**	0.25**	0.25**	0.24**
lh_parahippocampal_Thick	0.70	0.34	0.70	0.72	0.72	1	0.50	1.01	1.01	1.01	0.12**	0.87**	0.11**	0.11**	0.11**
rh_parahippocampal_Thick	0.65	0.35	0.65	0.64	0.64	0.96	0.51	0.96	0.96	0.95	0.16**	0.84**	0.16**	0.16**	0.16**
lh_parsopercularis_Thick	0.63	0.42	0.62	0.62	0.63	0.95	0.61	0.95	0.95	0.95	0.25**	0.76**	0.25**	0.25**	0.24**
rh_parsopercularis_Thick	0.68	0.44	0.66	0.67	0.66	0.97	0.65	0.97	0.97	0.97	0.25**	0.75**	0.26**	0.26**	0.26**
lh_parsorbitalis_Thick	0.62	0.44	0.62	0.61	0.62	0.94	0.65	0.94	0.93	0.94	0.18**	0.71**	0.19**	0.19**	0.18**
rh_parsorbitalis_Thick	0.66	0.45	0.67	0.66	0.65	0.99	0.67	0.99	0.99	0.99	0.21**	0.74**	0.20**	0.20**	0.20**
lh_parstriangularis_Thick	0.64	0.41	0.63	0.63	0.64	0.95	0.62	0.95	0.95	0.96	0.26**	0.76**	0.26**	0.26**	0.25**
rh_parstriangularis_Thick	0.66	0.39	0.67	0.66	0.66	0.95	0.60	0.95	0.95	0.95	0.27**	0.79**	0.27**	0.27**	0.26**
lh_pericalcarine_Thick	0.66	0.36	0.67	0.67	0.67	0.99	0.54	1	0.99	0.98	0.08**	0.83**	0.07**	0.07**	0.08**
rh_pericalcarine_Thick	0.69	0.32	0.66	0.68	0.68	1.03	0.50	1.05	1.03	1.03	0.07**	0.88**	0.07**	0.06**	0.06**
lh_postcentral_Thick	0.64	0.25	0.63	0.65	0.64	0.96	0.39	0.96	0.96	0.96	0.24**	0.91**	0.24**	0.24**	0.24**
rh_postcentral_Thick	0.65	0.20	0.64	0.64	0.64	0.96	0.34	0.96	0.96	0.96	0.26**	0.94**	0.27**	0.27**	0.26**
lh_posteriorcingulate_Thick	0.64	0.42	0.65	0.65	0.64	0.98	0.65	0.98	0.98	0.98	0.11**	0.74**	0.12**	0.12**	0.12**
rh_posteriorcingulate_Thick	0.63	0.41	0.64	0.64	0.63	0.97	0.64	0.97	0.97	0.97	0.19**	0.76**	0.20**	0.20**	0.19**

lh_precentral_Thick	0.61	0.23	0.6	0.61	0.61	0.95	0.39	0.97	0.95	0.95	0.26**	0.91**	0.26**	0.26**	0.25**
rh_precentral_Thick	0.63	0.22	0.6	0.62	0.62	0.96	0.36	1	0.96	0.96	0.29**	0.94**	0.29**	0.29**	0.28**
lh_precuneus_Thick	0.67	0.3	0.66	0.67	0.67	0.98	0.45	0.98	0.97	0.97	0.27**	0.89**	0.27**	0.27**	0.27**
rh_precuneus_Thick	0.66	0.31	0.64	0.66	0.65	0.95	0.47	0.95	0.95	0.95	0.25**	0.87**	0.25**	0.25**	0.24**
lh_rostralanteriorcingulate_Thick	0.63	0.45	0.63	0.63	0.63	0.97	0.71	0.97	0.97	0.97	0.20**	0.69**	0.20**	0.20**	0.20**
rh_rostralanteriorcingulate_Thick	0.62	0.48	0.62	0.62	0.63	0.96	0.74	0.96	0.96	0.96	0.16**	0.63**	0.17**	0.17**	0.17**
lh_rostralmiddlefrontal_Thick	0.61	0.32	0.6	0.6	0.6	0.97	0.49	0.97	0.97	0.97	0.31**	0.86**	0.31**	0.31**	0.31**
rh_rostralmiddlefrontal_Thick	0.66	0.31	0.66	0.66	0.66	0.95	0.46	0.95	0.95	0.95	0.31**	0.88**	0.32**	0.32**	0.31**
lh_superiorfrontal_Thick	0.61	0.23	0.61	0.61	0.6	0.93	0.34	0.93	0.93	0.93	0.32**	0.93**	0.32**	0.32**	0.32**
rh_superiorfrontal_Thick	0.63	0.22	0.64	0.64	0.64	0.95	0.34	0.95	0.94	0.94	0.34**	0.94**	0.34**	0.34**	0.34**
lh_superiorparietal_Thick	0.65	0.28	0.65	0.65	0.65	0.97	0.41	0.97	0.97	0.97	0.25**	0.90**	0.24**	0.24**	0.24**
rh_superiorparietal_Thick	0.64	0.25	0.63	0.64	0.64	0.95	0.37	0.95	0.95	0.95	0.26**	0.92**	0.27**	0.27**	0.26**
lh_superiortemporal_Thick	0.67	0.28	0.67	0.68	0.68	0.97	0.42	0.98	0.97	0.97	0.26**	0.90**	0.25**	0.26**	0.25**
rh_superiortemporal_Thick	0.65	0.29	0.65	0.65	0.65	0.96	0.43	0.97	0.96	0.96	0.29**	0.90**	0.29**	0.29**	0.3**
lh_supramarginal_Thick	0.60	0.34	0.59	0.59	0.59	0.94	0.51	0.94	0.94	0.94	0.29**	0.83**	0.29**	0.29**	0.28**
rh_supramarginal_Thick	0.66	0.32	0.64	0.64	0.65	0.96	0.50	0.96	0.96	0.96	0.27**	0.86**	0.27**	0.27**	0.27**
lh_transversetemporal_Thick	0.71	0.48	0.70	0.70	0.70	1.03	0.68	1.02	1.03	1.02	0.06*	0.73**	0.06*	0.04	0.06*
rh_transversetemporal_Thick	0.66	0.39	0.69	0.66	0.66	1	0.59	1	1	1	0.04**	0.80**	0.01	0.03	0.03
wb_TotalGray_Vol	0.32	0.03	0.31	0.31	0.31	0.46	0.05	0.46	0.46	0.46	0.88**	1**	0.88**	0.88**	0.88**
lh_Cortex_Vol	0.35	0.04	0.35	0.35	0.35	0.52	0.06	0.52	0.52	0.52	0.84**	1**	0.84**	0.84**	0.84**
rh_Cortex_Vol	0.36	0.04	0.36	0.36	0.35	0.53	0.07	0.53	0.52	0.53	0.83**	1**	0.84**	0.84**	0.84**
lh_caudalanteriorcingulate_Vol	0.56	0.31	0.56	0.55	0.55	0.86	0.49	0.86	0.86	0.86	0.51**	0.87**	0.51**	0.51**	0.51**
rh_caudalanteriorcingulate_Vol	0.61	0.32	0.61	0.60	0.6	0.97	0.51	0.97	0.96	0.96	0.27**	0.86**	0.27**	0.27**	0.27**
lh_caudalmiddlefrontal_Vol	0.55	0.36	0.54	0.53	0.54	0.80	0.55	0.81	0.8	0.80	0.52**	0.81**	0.52**	0.52**	0.52**
rh_caudalmiddlefrontal_Vol	0.56	0.36	0.56	0.57	0.56	0.83	0.57	0.83	0.83	0.82	0.49**	0.80**	0.50**	0.50**	0.50**
lh_cuneus_Vol	0.57	0.28	0.58	0.57	0.57	0.88	0.45	0.92	0.88	0.88	0.44**	0.88**	0.44**	0.44**	0.44**
rh_cuneus_Vol	0.55	0.25	0.55	0.55	0.56	0.85	0.40	0.88	0.85	0.85	0.45**	0.91**	0.46**	0.46**	0.46**
lh_entorhinal_Vol	0.55	0.35	0.55	0.55	0.55	0.90	0.58	0.94	0.90	0.90	0.35**	0.78**	0.35**	0.35**	0.35**
rh_entorhinal_Vol	0.58	0.38	0.58	0.58	0.57	0.93	0.62	0.96	0.92	0.92	0.29**	0.76**	0.30**	0.30**	0.30**

lh_fusiform_Vol	0.55	0.39	0.53	0.54	0.54	0.78	0.58	0.79	0.78	0.78	0.60**	0.80**	0.60**	0.60**	0.60**
rh_fusiform_Vol	0.50	0.35	0.5	0.5	0.49	0.76	0.54	0.77	0.76	0.76	0.61**	0.82**	0.61**	0.61**	0.61**
lh_inferiorparietal_Vol	0.54	0.34	0.55	0.54	0.54	0.81	0.50	0.81	0.80	0.80	0.58**	0.86**	0.58**	0.58**	0.58**
rh_inferiorparietal_Vol	0.53	0.32	0.52	0.52	0.51	0.76	0.50	0.77	0.76	0.76	0.65**	0.87**	0.65**	0.65**	0.65**
lh_inferiortemporal_Vol	0.50	0.33	0.50	0.50	0.50	0.76	0.50	0.77	0.76	0.76	0.64**	0.85**	0.64**	0.64**	0.64**
rh_inferiortemporal_Vol	0.50	0.30	0.49	0.51	0.51	0.76	0.48	0.76	0.76	0.76	0.65**	0.87**	0.65**	0.65**	0.65**
lh_insula_Vol	0.49	0.20	0.47	0.48	0.48	0.74	0.3	0.75	0.74	0.74	0.65**	0.95**	0.65**	0.65**	0.65**
rh_insula_Vol	0.48	0.19	0.47	0.48	0.49	0.73	0.28	0.73	0.73	0.72	0.66**	0.95**	0.66**	0.66**	0.66**
lh_isthmuscingulate_Vol	0.54	0.40	0.52	0.54	0.54	0.83	0.62	0.86	0.82	0.82	0.55**	0.78**	0.55**	0.55**	0.56**
rh_isthmuscingulate_Vol	0.58	0.41	0.57	0.58	0.58	0.90	0.64	0.93	0.90	0.90	0.48**	0.78**	0.48**	0.48**	0.48**
lh_lateraloccipital_Vol	0.52	0.27	0.52	0.52	0.51	0.79	0.41	0.80	0.79	0.79	0.58**	0.90**	0.58**	0.58**	0.58**
rh_lateraloccipital_Vol	0.55	0.28	0.55	0.55	0.56	0.81	0.43	0.82	0.81	0.81	0.57**	0.89**	0.57**	0.57**	0.57**
lh_lateralorbitofrontal_Vol	0.48	0.27	0.48	0.49	0.49	0.73	0.42	0.73	0.73	0.73	0.67**	0.90**	0.67**	0.67**	0.67**
rh_lateralorbitofrontal_Vol	0.50	0.29	0.50	0.49	0.49	0.72	0.43	0.72	0.72	0.72	0.67**	0.89**	0.67**	0.67**	0.67**
lh_lingual_Vol	0.55	0.24	0.55	0.55	0.56	0.86	0.38	0.87	0.86	0.86	0.39**	0.91**	0.39**	0.40**	0.39**
rh_lingual_Vol	0.60	0.25	0.58	0.59	0.59	0.91	0.40	0.91	0.91	0.91	0.34**	0.91**	0.35**	0.35**	0.34**
lh_medialorbitofrontal_Vol	0.54	0.39	0.54	0.53	0.54	0.81	0.56	0.81	0.81	0.81	0.58**	0.81**	0.58**	0.58**	0.58**
rh_medialorbitofrontal_Vol	0.52	0.36	0.52	0.51	0.51	0.82	0.55	0.82	0.82	0.82	0.57**	0.83**	0.57**	0.57**	0.57**
lh_middletemporal_Vol	0.49	0.28	0.49	0.49	0.50	0.74	0.41	0.74	0.74	0.74	0.68**	0.90**	0.68**	0.68**	0.68**
rh_middletemporal_Vol	0.47	0.28	0.47	0.47	0.48	0.75	0.43	0.75	0.74	0.75	0.69**	0.90**	0.69**	0.69**	0.69**
lh_paracentral_Vol	0.54	0.35	0.54	0.54	0.54	0.81	0.53	0.82	0.81	0.81	0.53**	0.82**	0.54**	0.54**	0.54**
rh_paracentral_Vol	0.54	0.36	0.53	0.55	0.55	0.86	0.54	0.87	0.86	0.86	0.48**	0.82**	0.48**	0.48**	0.48**
lh_parahippocampal_Vol	0.59	0.32	0.62	0.59	0.59	0.94	0.51	0.97	0.94	0.94	0.31**	0.86**	0.31**	0.31**	0.31**
rh_parahippocampal_Vol	0.63	0.39	0.65	0.64	0.64	0.96	0.61	0.98	0.96	0.96	0.32**	0.81**	0.32**	0.33**	0.32**
lh_parsopercularis_Vol	0.52	0.37	0.51	0.52	0.51	0.86	0.61	0.90	0.85	0.86	0.48**	0.78**	0.48**	0.48**	0.47**
rh_parsopercularis_Vol	0.55	0.40	0.54	0.55	0.54	0.86	0.63	0.89	0.86	0.86	0.48**	0.76**	0.48**	0.48**	0.48**
lh_parsorbitalis_Vol	0.56	0.37	0.56	0.55	0.55	0.86	0.57	0.87	0.86	0.87	0.49**	0.82**	0.48**	0.48**	0.48**
rh_parsorbitalis_Vol	0.58	0.38	0.57	0.59	0.59	0.88	0.58	0.90	0.88	0.88	0.47**	0.81**	0.48**	0.48**	0.47**
lh_parstriangularis_Vol	0.58	0.35	0.58	0.57	0.57	0.86	0.52	0.89	0.86	0.86	0.46**	0.84**	0.45**	0.45**	0.45**

rh_parstriangularis_Vol	0.59	0.42	0.6	0.59	0.58	0.89	0.65	0.94	0.89	0.89	0.41**	0.73**	0.42**	0.42**	0.42**
lh_pericalcarine_Vol	0.62	0.22	0.59	0.6	0.6	0.91	0.35	0.99	0.91	0.91	0.30**	0.93**	0.30**	0.30**	0.3**
rh_pericalcarine_Vol	0.61	0.20	0.61	0.61	0.61	0.93	0.30	1	0.92	0.92	0.32**	0.95**	0.32**	0.32**	0.31**
lh_postcentral_Vol	0.52	0.28	0.53	0.52	0.52	0.78	0.44	0.79	0.78	0.78	0.60**	0.89**	0.60**	0.60**	0.6**
rh_postcentral_Vol	0.54	0.26	0.54	0.55	0.56	0.83	0.41	0.84	0.83	0.83	0.55**	0.91**	0.56**	0.55**	0.56**
lh_posteriorcingulate_Vol	0.58	0.43	0.58	0.58	0.57	0.84	0.65	0.84	0.84	0.84	0.55**	0.77**	0.55**	0.55**	0.55**
rh_posteriorcingulate_Vol	0.55	0.43	0.55	0.55	0.55	0.87	0.67	0.87	0.87	0.87	0.52**	0.75**	0.53**	0.53**	0.53**
lh_precentral_Vol	0.48	0.26	0.48	0.48	0.48	0.75	0.41	0.75	0.75	0.75	0.62**	0.91**	0.62**	0.62**	0.62**
rh_precentral_Vol	0.49	0.24	0.48	0.48	0.48	0.77	0.40	0.77	0.77	0.77	0.59**	0.91**	0.60**	0.60**	0.59**
lh_precuneus_Vol	0.52	0.25	0.49	0.50	0.50	0.76	0.39	0.76	0.75	0.75	0.62**	0.91**	0.63**	0.63**	0.63**
rh_precuneus_Vol	0.49	0.27	0.49	0.49	0.49	0.76	0.42	0.76	0.76	0.76	0.66**	0.91**	0.67**	0.67**	0.67**
lh_rostralanteriorcingulate_Vol	0.52	0.40	0.52	0.52	0.53	0.80	0.60	0.81	0.80	0.80	0.58**	0.78**	0.58**	0.58**	0.58**
rh_rostralanteriorcingulate_Vol	0.56	0.43	0.56	0.56	0.55	0.88	0.67	0.89	0.88	0.88	0.47**	0.73**	0.47**	0.47**	0.47**
lh_rostralmiddlefrontal_Vol	0.50	0.32	0.49	0.49	0.49	0.73	0.48	0.75	0.73	0.73	0.65**	0.86**	0.65**	0.66**	0.65**
rh_rostralmiddlefrontal_Vol	0.51	0.29	0.51	0.5	0.5	0.74	0.45	0.76	0.74	0.74	0.63**	0.88**	0.64**	0.64**	0.64**
lh_superiorfrontal_Vol	0.44	0.24	0.44	0.44	0.44	0.67	0.38	0.68	0.67	0.67	0.71**	0.91**	0.71**	0.71**	0.7**
rh_superiorfrontal_Vol	0.44	0.20	0.43	0.43	0.42	0.65	0.32	0.66	0.65	0.65	0.72**	0.94**	0.72**	0.72**	0.72**
lh_superiorparietal_Vol	0.57	0.34	0.57	0.56	0.57	0.83	0.50	0.83	0.83	0.83	0.51**	0.85**	0.52**	0.52**	0.51**
rh_superiorparietal_Vol	0.55	0.33	0.55	0.55	0.55	0.83	0.51	0.83	0.83	0.83	0.55**	0.85**	0.55**	0.55**	0.55**
lh_superiortemporal_Vol	0.49	0.25	0.48	0.48	0.48	0.75	0.40	0.75	0.74	0.75	0.65**	0.92**	0.65**	0.65**	0.65**
rh_superiortemporal_Vol	0.50	0.27	0.50	0.49	0.49	0.75	0.40	0.75	0.75	0.75	0.64**	0.91**	0.65**	0.65**	0.64**
lh_supramarginal_Vol	0.55	0.40	0.53	0.54	0.54	0.83	0.59	0.84	0.83	0.83	0.59**	0.81**	0.59**	0.59**	0.58**
rh_supramarginal_Vol	0.52	0.30	0.51	0.52	0.51	0.77	0.49	0.77	0.77	0.77	0.60**	0.86**	0.60**	0.60**	0.59**
lh_transversetemporal_Vol	0.60	0.42	0.57	0.59	0.61	0.90	0.64	0.92	0.90	0.90	0.39**	0.74**	0.39**	0.39**	0.39**
rh_transversetemporal_Vol	0.58	0.34	0.58	0.57	0.57	0.86	0.5	0.89	0.85	0.85	0.42**	0.84**	0.42**	0.42**	0.42**
wholeBrainWMHvol	0.56	0.05	0.56	0.56	0.57	0.87	0.08	0.9	0.86	0.86	0.42**	1**	0.43**	0.43**	0.43**
PVWMHvol	0.56	0.06	0.56	0.56	0.56	0.88	0.10	0.89	0.87	0.87	0.41**	0.99**	0.42**	0.42**	0.42**
DWMHvol	0.52	0.12	0.51	0.52	0.52	0.90	0.39	0.90	0.90	0.90	0.36**	0.97**	0.37**	0.37**	0.37**
Lfrontal_WMHvol	0.44	0.13	0.21	0.50	0.54	0.96	0.61	1.12	0.95	0.97	0.29**	0.87**	0.3**	0.30**	0.27**

Rfrontal_WMHvol	0.43	0.13	0.21	0.48	0.53	0.95	0.59	1.1	0.94	0.96	0.27**	0.89**	0.28**	0.29**	0.25**
Ltemporal_WMHvol	0.62	0.60	0.21	0.70	0.67	1	0.89	1.24	1	1.01	0.26**	0.53**	0.23**	0.26**	0.20**
Rtemporal_WMHvol	0.70	0.67	0.23	0.77	0.73	1.01	0.91	1.28	1.01	1.02	0.20**	0.52**	0.19**	0.20**	0.13**
Lparietal_WMHvol	0.28	0.13	0.21	0.41	0.45	0.95	0.72	1.05	0.93	0.95	0.35**	0.86**	0.35**	0.35**	0.32**
Rparietal_WMHvol	0.26	0.12	0.21	0.37	0.39	0.97	0.77	1.06	0.96	0.97	0.35**	0.87**	0.35**	0.36**	0.34**
Loccipital_WMHvol	0.76	0.12	0.23	0.72	0.70	0.98	0.35	1.22	0.98	0.98	0.13**	0.89**	0.13**	0.13**	0.08**
Roccipital_WMHvol	0.51	0.1	0.17	0.54	0.56	0.97	0.35	1.12	0.97	0.97	0.13**	0.90**	0.14**	0.14**	0.12**
Lcerebellum_WMHvol	0	0	0	0.28	0.02	1.07	1.07	1.07	1.03	1.07	0.04	0.21**	-0.01	0.01	0.02
Rcerebellum_WMHvol	0	0	0	0.22	0.01	1.12	1.12	1.12	1.08	1.12	0	0.24**	-0.01	0.04	0.03
Brainstem_WMHvol	0.18	0.16	0.17	0.38	0.40	1.06	0.73	1.10	1.02	1.02	0.16**	0.59**	0.17**	0.17**	0.13**
lAAH_WMHvol	0.31	0.16	0.27	0.32	0.33	0.89	0.73	0.94	0.89	0.89	0.33**	0.81**	0.35**	0.35**	0.33**
rAAH_WMHvol	0.30	0.17	0.31	0.32	0.31	0.93	0.76	0.95	0.92	0.92	0.31**	0.82**	0.32**	0.32**	0.33**
lMAH_WMHvol	0.48	0.21	0.48	0.49	0.5	0.88	0.56	0.88	0.88	0.88	0.35**	0.88**	0.35**	0.35**	0.34**
rMAH_WMHvol	0.50	0.19	0.51	0.51	0.51	0.91	0.47	0.90	0.90	0.90	0.38**	0.91**	0.39**	0.39**	0.39**
lAAML_WMHvol	0.48	0.27	0.48	0.49	0.49	0.93	0.63	0.97	0.92	0.92	0.40**	0.84**	0.40**	0.40**	0.40**
rAAML_WMHvol	0.55	0.32	0.53	0.56	0.56	0.94	0.63	0.98	0.93	0.93	0.43**	0.83**	0.44**	0.44**	0.44**
lAAC_WMHvol	0.59	0.23	0.55	0.58	0.58	1.03	0.66	1.08	1.02	1.02	0.28**	0.89**	0.29**	0.29**	0.29**
rAAC_WMHvol	0.55	0.29	0.53	0.55	0.55	0.98	0.70	1.04	0.97	0.98	0.23**	0.82**	0.24**	0.25**	0.23**
lMALL_WMHvol	0.57	0.2	0.57	0.58	0.58	0.89	0.38	0.91	0.89	0.89	0.38**	0.92**	0.38**	0.38**	0.38**
rMALL_WMHvol	0.63	0.29	0.61	0.6	0.6	0.92	0.45	0.92	0.92	0.92	0.33**	0.86**	0.34**	0.34**	0.34**
lPATMP_WMHvol	0.60	0.47	0.59	0.61	0.61	0.93	0.71	0.97	0.93	0.93	0.15**	0.67**	0.16**	0.15**	0.15**
rPATMP_WMHvol	0.59	0.43	0.60	0.58	0.58	0.92	0.68	0.95	0.92	0.92	0.20**	0.69**	0.19**	0.19**	0.19**
lPAH_WMHvol	0.31	0.14	0.26	0.35	0.36	1.03	0.8	1.09	1.02	1.02	0.23**	0.85**	0.24**	0.24**	0.23**
rPAH_WMHvol	0.31	0.12	0.27	0.33	0.34	0.96	0.72	1	0.95	0.96	0.27**	0.89**	0.28**	0.27**	0.27**
lPAC_WMHvol	0.38	0	0	0.46	0.04	0.99	1.10	1.11	0.97	1.10	0.22**	0.62**	-0.01	0.21**	0.01
rPAC_WMHvol	0	0	0	0.33	0.02	1.10	1.10	1.10	1.01	1.09	0.12**	0.35**	0	0.16**	0.02

This table presents a comprehensive comparison of five models (prior-cVAE, posterior-cVAE, GAMLSS, MFPR, and HBR) across various brain measures

using three key performance metrics. These metrics were computed using predicted and actual data from the hold-out datasets to assess model generalisation. For Spearman's correlation coefficient, * indicates statistical significance with ($p < 0.05$), while ** indicates ($p < 0.01$). Abbreviations: cVAE = conditional Variational Autoencoder; GAMLSS = Generalised Additive Models for Location, Scale and Shape; MFPR = Multivariate Fractional Polynomial Regression; HBR = Hierarchical Bayesian Regression. See Table S1 for abbreviations of lobar and arterial regions.

Table S7 Correlations between covariates and z-score of selected measures by model on the hold-out dataset.

		Prior-cVAE		Posterior-cVAE		GAMLSS		MFPR		HBR	
		Coefficient	*p*-value	Coefficient	*p*-value	Coefficient	*p*-value	Coefficient	*p*-value	Coefficient	*p*-value
Age	Mean thickness of the left insula	-0.026	0.447	0.012	0.919	0.038	0.541	0.044	0.311	0.033	0.569
	Mean thickness of the right insula	-0.031	0.357	-0.016	0.906	0.002	0.972	0.008	0.861	0.002	0.977
	Volume of whole-brain subcortical grey matter volume	-0.033	0.325	0.038	0.525	-0.016	0.837	-0.022	0.699	-0.009	0.918
	Volume of the 5th ventricle	0.217	**<0.001**	0.010	0.919	0.140	**<0.001**	-0.058	0.150	0.003	0.960
	Volume of whole-brain cortical grey matter	-0.025	0.447	0.064	0.160	-0.011	0.905	0.004	0.921	-0.015	0.869
	Volume of whole-brain WMH	0.041	0.145	-0.040	0.511	-0.036	0.541	-0.044	0.311	0.010	0.901
	WMH volume of the left cerebellum	0.033	0.325	0.024	0.789	-0.139	**<0.001**	-0.017	0.743	0.015	0.869
	WMH volume of the right cerebellum	0.042	0.231	0.055	0.263	0.071	0.135	0.009	0.861	-0.025	0.691
	WMH volume of the left posterior artery callosal	-0.445	**<0.001**	0.149	**<0.001**	0.146	**<0.001**	-0.398	**<0.001**	0.167	**<0.001**
	WMH volume of the right posterior artery callosal	-0.105	**<0.001**	0.087	**0.021**	-0.046	0.377	-0.548	**<0.001**	0.121	**<0.001**

Intracranial Volume	Mean thickness of the left insula	0.028	0.441	0.020	0.713	0.037	0.566	0.035	0.566	0.045	0.421
	Mean thickness of the right insula	0.041	0.239	0.045	0.379	0.046	0.452	0.045	0.377	0.044	0.463
	Volume of whole-brain subcortical grey matter volume	0.015	0.705	-0.075	**0.040**	0.001	0.996	0.002	0.985	-0.009	0.943
	Volume of the 5th ventricle	0.377	**<0.001**	0.392	**<0.001**	-0.031	0.653	-0.072	**0.049**	-0.043	0.476
	Volume of whole-brain cortical grey matter	0.021	0.562	-0.044	0.402	-0.025	0.753	-0.018	0.846	-0.035	0.590
	Volume of whole-brain WMH	0.045	0.178	0.052	0.276	0.041	0.502	0.030	0.685	0.035	0.590
	WMH volume of the left cerebellum	-0.284	**<0.001**	0.152	**<0.001**	0.040	0.502	-0.557	**<0.001**	-0.055	0.251
	WMH volume of the right cerebellum	0.216	**<0.001**	-0.013	0.846	-0.005	0.975	-0.585	**<0.001**	-0.012	0.943
	WMH volume of the left posterior artery callosal	-0.022	0.551	0.003	0.967	0.015	0.940	-0.133	**<0.001**	-0.011	0.943
	WMH volume of the right posterior artery callosal	-0.167	**<0.001**	-0.027	0.637	0.029	0.703	-0.141	**<0.001**	-0.023	0.736
Sex	Mean thickness of the left insula	0.044	0.503	0.036	0.582	0.044	0.472	0.047	0.727	0.055	0.262
	Mean thickness of the right insula	0.001	0.992	0.011	0.916	0.013	0.912	0.012	0.997	0.005	0.952
	Volume of whole-brain subcortical grey matter volume	0.001	0.992	-0.013	0.895	0.008	0.947	-0.011	0.997	-0.010	0.898
	Volume of the 5th ventricle	0.006	0.910	0.175	**<0.001**	-0.010	0.913	-0.024	0.917	-0.005	0.952

	Volume of whole-brain cortical grey matter	0.013	0.833	-0.049	0.285	-0.026	0.704	-0.023	0.917	-0.029	0.703
	Volume of whole-brain WMH	-0.015	0.833	0.016	0.864	0.017	0.870	0.019	0.968	0.025	0.703
	WMH volume of the left cerebellum	-0.004	0.941	0.091	**0.011**	0.001	0.993	-0.008	0.997	0.026	0.703
	WMH volume of the right cerebellum	-0.023	0.773	0.050	0.285	0.035	0.665	-0.035	0.917	0.028	0.703
	WMH volume of the left posterior artery callosal	0.035	0.519	0.052	0.285	0.028	0.682	0.015	0.997	0.023	0.703
	WMH volume of the right posterior artery callosal	-0.026	0.731	0.007	0.967	-0.044	0.483	-0.019	0.968	0.003	0.956

This table summarises Spearman's correlation coefficients and p-values for the relationship between age and z-scores of selected imaging-derived phenotypes, derived from five models (prior-cVAE, posterior-cVAE, GAMLSS, MFPR, HBR) across various brain regions on the hold-out dataset. P-values were corrected for multiple comparisons using the False Discovery Rate (FDR) method. Abbreviations: cVAE = conditional Variational Autoencoder; GAMLSS = Generalised Additive Models for Location, Scale and Shape; MFPR = Multivariate Fractional Polynomial Regression; HBR = Hierarchical Bayesian Regression.

Table S8 Spearman's correlations between Z-scores of brain measures and hypertension levels across models.

	Prior-cVAE		Posterior-cVAE		GAMLSS		MFPR		HBR	
	r_s	p-value	r_s	p-value	r_s	p-value	r_s	p-value	r_s	p-value
wb_SubCortGray_Vol	0.011	0.457	0.021	0.208	0.026	**0.008**	0.029	**0.003**	0.030	**0.001**
wb_3rd-Ventricle_Vol	0.027	**0.004**	0.009	0.9	0.011	0.588	0.007	0.829	0.017	0.157
wb_4th-Ventricle_Vol	0.016	0.174	-0.001	0.978	0.008	0.759	0.007	0.829	0.005	0.919
wb_5th-Ventricle_Vol	0.064	**<0.001**	-0.008	0.935	0.010	0.649	-0.003	0.95	0.004	0.935
lh_Accumbens-area_Vol	-0.011	0.457	0.015	0.583	0.008	0.749	0.011	0.585	0.004	0.935
rh_Accumbens-area_Vol	-0.011	0.424	0.001	0.978	0.007	0.816	0.009	0.737	0.007	0.826
lh_Amygdala_Vol	-0.012	0.377	-0.005	0.975	0.007	0.829	0.006	0.874	0.007	0.81
rh_Amygdala_Vol	0	0.986	0.005	0.975	0.016	0.239	0.017	0.19	0.016	0.226
wb_Brain-Stem_Vol	-0.014	0.265	0.007	0.94	-0.001	0.983	-0.002	0.978	0	1
wb_CC-Anterior_Vol	0.017	0.114	0.005	0.975	0.023	**0.035**	0.024	**0.024**	0.021	0.076
wb_CC-Central_Vol	-0.001	0.978	0.003	0.978	0.010	0.664	0.023	**0.044**	-0.008	0.79
wb_CC-Mid-Anterior_Vol	0.001	0.983	0.001	0.983	0.014	0.364	0.023	**0.043**	-0.003	0.95
wb_CC-Mid-Posterior_Vol	0.002	0.975	0	1	0.007	0.826	0.009	0.737	0.004	0.935
wb_CC-Posterior_Vol	0.015	0.214	0.01	0.841	0.020	0.09	0.021	0.075	0.018	0.132
wb_CSF_Vol	-0.011	0.424	-0.026	0.065	-0.023	**0.033**	-0.03	**0.002**	-0.018	0.136
lh_Caudate_Vol	0.035	**<0.001**	0.002	0.978	0.034	**<0.001**	0.033	**<0.001**	0.034	**<0.001**

rh_Caudate_Vol	0.034	**<0.001**	0	1	0.033	**<0.001**	0.031	**0.001**	0.032	**<0.001**
lh_Cerebellum-Cortex_Vol	0.003	0.96	0.004	0.978	0.006	0.848	0.007	0.829	0.007	0.833
rh_Cerebellum-Cortex_Vol	0.002	0.978	-0.010	0.886	0.007	0.829	0.008	0.79	0.006	0.862
lh_Cerebellum-White-Matter_Vol	-0.023	0.021	-0.007	0.935	-0.009	0.749	-0.008	0.799	-0.007	0.829
rh_Cerebellum-White-Matter_Vol	-0.010	0.501	0.011	0.807	0.001	0.983	-0.003	0.948	0.001	0.978
lh_CerebralWhiteMatter_Vol	-0.010	0.517	-0.005	0.975	0.005	0.919	0.007	0.829	0.007	0.828
rh_CerebralWhiteMatter_Vol	-0.009	0.565	0.006	0.96	0.005	0.9	0.008	0.807	0.004	0.935
lh_Hippocampus_Vol	-0.008	0.62	0.012	0.749	0.012	0.487	0.014	0.37	0.012	0.506
rh_Hippocampus_Vol	-0.005	0.841	0.004	0.978	0.015	0.265	0.016	0.222	0.017	0.197
lh_Inf-Lat-Vent_Vol	0.040	**<0.001**	0.015	0.583	0.020	0.090	0.020	0.098	0.022	**0.045**
rh_Inf-Lat-Vent_Vol	0.032	**<0.001**	0.013	0.682	0.019	0.118	0.018	0.18	0.020	0.091
lh_Lateral-Ventricle_Vol	0.028	**0.003**	-0.002	0.978	0.007	0.829	0.006	0.841	0.005	0.918
rh_Lateral-Ventricle_Vol	0.026	**0.005**	0.004	0.975	0.008	0.807	0.007	0.819	0.005	0.932
lh_Pallidum_Vol	0.001	0.983	-0.004	0.975	0.010	0.62	0.010	0.643	0.014	0.347
rh_Pallidum_Vol	0.001	0.978	-0.003	0.978	0.011	0.562	0.011	0.572	0.014	0.337
lh_Putamen_Vol	0.012	0.375	-0.007	0.935	0.017	0.189	0.017	0.19	0.020	0.085
rh_Putamen_Vol	0.015	0.199	0.008	0.935	0.02	0.086	0.021	0.086	0.025	**0.014**
lh_Thalamus-Proper_Vol	-0.003	0.94	0.001	0.978	0.008	0.759	0.005	0.919	0.011	0.578
rh_Thalamus-Proper_Vol	0	0.983	-0.002	0.978	0.006	0.848	0.006	0.848	0.007	0.81
lh_VentralDC_Vol	-0.025	0.009	-0.012	0.749	-0.007	0.826	-0.008	0.79	-0.009	0.749

rh_VentralDC_Vol	-0.020	0.048	-0.002	0.978	-0.004	0.928	-0.004	0.935	-0.004	0.935
wb_VentricleChoroid_Vol	0.029	**0.002**	0.011	0.794	0.008	0.795	0.007	0.822	0.009	0.706
lh_caudalanteriorcingulate_Thick	-0.028	**0.002**	-0.003	0.978	-0.017	0.174	-0.018	0.145	-0.022	**0.044**
rh_caudalanteriorcingulate_Thick	-0.020	0.06	-0.002	0.978	-0.015	0.269	-0.014	0.341	-0.019	0.11
lh_caudalmiddlefrontal_Thick	-0.018	0.11	0.002	0.978	-0.001	0.983	0.001	0.983	0	0.995
rh_caudalmiddlefrontal_Thick	-0.011	0.417	-0.003	0.978	0.003	0.938	0.005	0.935	0.004	0.935
lh_cuneus_Thick	0.014	0.258	0.016	0.565	0.022	0.06	0.021	0.065	0.019	0.105
rh_cuneus_Thick	0.005	0.89	-0.007	0.935	0.010	0.647	0.01	0.643	0.009	0.682
lh_entorhinal_Thick	-0.024	**0.013**	0.005	0.975	-0.005	0.919	0	0.998	-0.006	0.862
rh_entorhinal_Thick	-0.027	**0.003**	0	0.986	-0.010	0.65	-0.007	0.826	-0.016	0.199
lh_fusiform_Thick	-0.014	0.258	0.013	0.749	0.002	0.975	0.003	0.96	-0.002	0.975
rh_fusiform_Thick	-0.005	0.89	0.016	0.565	0.009	0.729	0.01	0.643	0.006	0.869
lh_inferiorparietal_Thick	-0.006	0.81	0.007	0.935	0.013	0.414	0.014	0.341	0.010	0.682
rh_inferiorparietal_Thick	-0.011	0.424	-0.007	0.935	0.005	0.919	0.007	0.829	0.004	0.935
lh_inferiortemporal_Thick	-0.029	**0.002**	-0.022	0.208	-0.020	0.095	-0.02	0.098	-0.022	0.044
rh_inferiortemporal_Thick	-0.025	**0.01**	-0.012	0.781	-0.010	0.62	-0.01	0.64	-0.018	0.136
lh_insula_Thick	-0.033	**<0.001**	-0.003	0.978	-0.017	0.205	-0.016	0.226	-0.020	0.086
rh_insula_Thick	-0.043	**<0.001**	-0.021	0.208	-0.033	**<0.001**	-0.032	**0.001**	-0.035	**<0.001**
lh_isthmuscingulate_Thick	-0.009	0.62	0.001	0.983	-0.004	0.935	-0.003	0.948	-0.003	0.937
rh_isthmuscingulate_Thick	-0.002	0.978	0.001	0.978	0.004	0.935	0.002	0.978	0.002	0.96

lh_lateraloccipital_Thick	-0.007	0.749	-0.017	0.424	0.007	0.829	0.008	0.759	0.004	0.935
rh_lateraloccipital_Thick	0.001	0.978	-0.003	0.978	0.011	0.565	0.013	0.487	0.008	0.749
lh_lateralorbitofrontal_Thick	-0.035	**<0.001**	-0.004	0.978	-0.018	0.166	-0.016	0.222	-0.024	**0.026**
rh_lateralorbitofrontal_Thick	-0.020	0.057	0.009	0.901	-0.009	0.729	-0.008	0.752	-0.009	0.682
lh_lingual_Thick	0.001	0.978	0.004	0.975	0.011	0.562	0.012	0.517	0.008	0.749
rh_lingual_Thick	0.002	0.964	0.001	0.978	0.005	0.9	0.006	0.888	0.005	0.935
lh_medialorbitofrontal_Thick	-0.030	**0.001**	-0.006	0.95	-0.019	0.118	-0.02	0.098	-0.020	0.096
rh_medialorbitofrontal_Thick	-0.009	0.565	0.012	0.749	0	0.986	-0.003	0.96	-0.005	0.918
lh_middletemporal_Thick	-0.031	**0.001**	-0.010	0.84	-0.02	0.092	-0.020	0.105	-0.02	0.096
rh_middletemporal_Thick	-0.026	**0.006**	-0.006	0.975	-0.016	0.239	-0.018	0.149	-0.017	0.17
lh_paracentral_Thick	-0.029	**0.001**	-0.004	0.975	-0.013	0.394	-0.01	0.643	-0.019	0.121
rh_paracentral_Thick	-0.032	**<0.001**	-0.012	0.793	-0.021	0.06	-0.017	0.199	-0.019	0.109
lh_parahippocampal_Thick	-0.017	0.132	-0.001	0.983	0	1	0.001	0.978	-0.002	0.975
rh_parahippocampal_Thick	-0.016	0.145	-0.001	0.983	-0.004	0.935	-0.003	0.96	-0.010	0.606
lh_parsopercularis_Thick	-0.019	0.086	0.002	0.978	-0.003	0.95	-0.001	0.983	-0.008	0.796
rh_parsopercularis_Thick	-0.019	0.086	-0.005	0.975	-0.007	0.829	-0.005	0.932	-0.009	0.749
lh_parsorbitalis_Thick	-0.005	0.89	0.017	0.424	0.011	0.59	0.012	0.535	0.008	0.749
rh_parsorbitalis_Thick	-0.019	0.076	0.006	0.95	-0.005	0.919	-0.004	0.935	-0.005	0.935
lh_parstriangularis_Thick	-0.010	0.533	0.013	0.682	0	0.991	0.008	0.807	0.003	0.96
rh_parstriangularis_Thick	-0.009	0.565	0.009	0.901	-0.001	0.983	0	0.986	0.006	0.862

lh_pericalcarine_Thick	0.002	0.975	0.008	0.935	0.009	0.749	0.009	0.749	0.008	0.78
rh_pericalcarine_Thick	0.005	0.841	0.002	0.978	0.008	0.749	0.011	0.562	0.008	0.79
lh_postcentral_Thick	-0.008	0.62	0.013	0.682	0.007	0.829	0.009	0.749	0.004	0.935
rh_postcentral_Thick	-0.005	0.838	0.005	0.975	0.007	0.829	0.009	0.749	0.006	0.862
lh_posteriorcingulate_Thick	-0.022	**0.026**	-0.002	0.978	-0.012	0.528	-0.01	0.643	-0.012	0.501
rh_posteriorcingulate_Thick	-0.023	**0.021**	-0.013	0.682	-0.017	0.18	-0.017	0.199	-0.016	0.228
lh_precentral_Thick	-0.024	**0.012**	0.012	0.749	-0.006	0.839	-0.003	0.95	-0.013	0.449
rh_precentral_Thick	-0.025	**0.009**	-0.009	0.901	-0.011	0.59	-0.008	0.752	-0.014	0.373
lh_precuneus_Thick	-0.006	0.834	-0.011	0.809	0.013	0.466	0.014	0.37	0.01	0.61
rh_precuneus_Thick	0.002	0.975	0.002	0.978	0.019	0.118	0.021	0.065	0.017	0.177
lh_rostralanteriorcingulate_Thick	-0.025	**0.009**	-0.003	0.978	-0.015	0.305	-0.015	0.313	-0.013	0.389
rh_rostralanteriorcingulate_Thick	-0.022	**0.026**	-0.005	0.975	-0.015	0.265	-0.017	0.200	-0.018	0.141
lh_rostralmiddlefrontal_Thick	-0.015	0.22	0.002	0.978	0.007	0.829	0.008	0.810	0.005	0.932
rh_rostralmiddlefrontal_Thick	-0.018	0.098	-0.007	0.935	-0.004	0.935	-0.002	0.978	-0.004	0.935
lh_superiorfrontal_Thick	-0.015	0.201	0.007	0.935	0.006	0.848	0.009	0.749	0.005	0.919
rh_superiorfrontal_Thick	-0.014	0.257	-0.004	0.975	0.003	0.95	0.006	0.888	0.004	0.935
lh_superiorparietal_Thick	0.011	0.424	0.019	0.295	0.030	**0.001**	0.03	**0.001**	0.027	**0.007**
rh_superiorparietal_Thick	0.014	0.239	0.025	0.071	0.030	**0.001**	0.032	**0.001**	0.029	**0.002**
lh_superiortemporal_Thick	-0.029	**0.002**	0.004	0.975	-0.011	0.59	-0.009	0.749	-0.012	0.462
rh_superiortemporal_Thick	-0.022	**0.025**	0.014	0.643	-0.006	0.841	-0.004	0.935	-0.01	0.657

lh_supramarginal_Thick	-0.021	**0.037**	-0.008	0.919	-0.005	0.919	-0.003	0.96	-0.008	0.793
rh_supramarginal_Thick	-0.018	0.092	-0.004	0.978	-0.003	0.95	-0.002	0.978	-0.005	0.928
lh_transversetemporal_Thick	-0.013	0.28	-0.006	0.95	-0.009	0.749	-0.01	0.643	-0.009	0.682
rh_transversetemporal_Thick	-0.007	0.786	0.002	0.978	-0.005	0.919	-0.001	0.983	-0.004	0.935
wb_TotalGray_Vol	-0.027	**0.004**	0.003	0.978	-0.005	0.92	-0.006	0.887	-0.004	0.935
lh_Cortex_Vol	-0.031	**0.001**	-0.004	0.975	-0.011	0.562	-0.012	0.517	-0.014	0.343
rh_Cortex_Vol	-0.03	**0.001**	-0.009	0.916	-0.013	0.424	-0.014	0.37	-0.016	0.222
lh_caudalanteriorcingulate_Vol	-0.016	0.188	0.005	0.975	-0.013	0.453	-0.012	0.514	-0.012	0.506
rh_caudalanteriorcingulate_Vol	-0.018	0.098	-0.005	0.975	-0.017	0.193	-0.017	0.2	-0.019	0.115
lh_caudalmiddlefrontal_Vol	-0.017	0.133	-0.015	0.583	-0.012	0.517	-0.011	0.585	-0.011	0.582
rh_caudalmiddlefrontal_Vol	-0.006	0.81	-0.001	0.983	-0.001	0.983	-0.001	0.983	-0.003	0.95
lh_cuneus_Vol	0.004	0.89	0.001	0.983	0.014	0.391	0.01	0.682	0.011	0.565
rh_cuneus_Vol	0.003	0.937	0.004	0.978	0.009	0.715	0.007	0.818	0.011	0.574
lh_entorhinal_Vol	-0.013	0.305	-0.003	0.978	0.002	0.978	0.002	0.978	0.003	0.935
rh_entorhinal_Vol	-0.013	0.275	-0.001	0.978	-0.001	0.983	0	0.986	0	0.986
lh_fusiform_Vol	-0.014	0.229	-0.004	0.978	-0.002	0.978	-0.002	0.975	0	0.995
rh_fusiform_Vol	-0.009	0.59	0.012	0.749	0.001	0.983	-0.001	0.983	-0.001	0.978
lh_inferiorparietal_Vol	-0.025	**0.008**	-0.007	0.935	-0.016	0.209	-0.017	0.199	-0.016	0.2
rh_inferiorparietal_Vol	-0.015	0.211	0.011	0.807	-0.007	0.829	-0.007	0.829	-0.008	0.749
lh_inferiortemporal_Vol	-0.028	**0.002**	-0.004	0.975	-0.02	0.09	-0.019	0.122	-0.019	0.11

rh_inferiortemporal_Vol	-0.016	0.188	0.004	0.978	-0.006	0.841	-0.006	0.848	-0.006	0.858
lh_insula_Vol	-0.006	0.796	0.012	0.749	0.001	0.983	-0.003	0.96	0.008	0.799
rh_insula_Vol	-0.006	0.826	0.020	0.275	-0.001	0.983	-0.007	0.814	-0.002	0.975
lh_isthmuscingulate_Vol	-0.010	0.499	0.001	0.978	-0.004	0.935	-0.004	0.935	-0.003	0.96
rh_isthmuscingulate_Vol	-0.006	0.829	-0.001	0.978	0.001	0.983	0.001	0.978	0.004	0.935
lh_lateraloccipital_Vol	-0.001	0.978	0.007	0.935	0.011	0.59	0.010	0.682	0.011	0.565
rh_lateraloccipital_Vol	-0.004	0.935	0	1	0.005	0.919	0.004	0.935	0.004	0.935
lh_lateralorbitofrontal_Vol	-0.026	**0.007**	0.007	0.935	-0.014	0.355	-0.014	0.347	-0.014	0.339
rh_lateralorbitofrontal_Vol	-0.028	**0.003**	-0.002	0.978	-0.018	0.132	-0.019	0.122	-0.021	0.063
lh_lingual_Vol	-0.004	0.89	-0.007	0.935	0.005	0.919	0.005	0.89	0.003	0.960
rh_lingual_Vol	0.002	0.975	0.010	0.849	0.010	0.65	0.010	0.682	0.010	0.628
lh_medialorbitofrontal_Vol	-0.031	**0.001**	-0.008	0.919	-0.020	0.088	-0.021	0.086	-0.018	0.136
rh_medialorbitofrontal_Vol	-0.028	**0.002**	-0.013	0.682	-0.020	0.093	-0.023	0.037	-0.019	0.115
lh_middletemporal_Vol	-0.040	**<0.001**	-0.018	0.39	-0.032	**0.001**	-0.032	**0.001**	-0.031	**0.001**
rh_middletemporal_Vol	-0.026	**0.005**	-0.003	0.978	-0.019	0.109	-0.019	0.122	-0.018	0.139
lh_paracentral_Vol	-0.022	**0.023**	0.005	0.975	-0.013	0.459	-0.014	0.37	-0.013	0.424
rh_paracentral_Vol	-0.014	0.258	0.014	0.682	-0.008	0.807	-0.009	0.682	-0.009	0.73
lh_parahippocampal_Vol	-0.015	0.21	-0.006	0.975	0.003	0.95	0.004	0.94	0.002	0.975
rh_parahippocampal_Vol	-0.015	0.206	-0.002	0.978	0.001	0.983	0.001	0.983	0	1
lh_parsopercularis_Vol	-0.008	0.62	-0.002	0.978	-0.002	0.975	-0.004	0.94	-0.003	0.937

rh_parsopercularis_Vol	-0.010	0.551	-0.003	0.978	-0.006	0.848	-0.006	0.888	-0.007	0.829
lh_parsorbitalis_Vol	-0.012	0.373	0.004	0.978	0	1	0	0.986	0.005	0.919
rh_parsorbitalis_Vol	-0.020	0.052	-0.010	0.841	-0.011	0.59	-0.009	0.716	-0.011	0.574
lh_parstriangularis_Vol	-0.002	0.975	0.005	0.975	0.002	0.975	0.002	0.975	-0.002	0.975
rh_parstriangularis_Vol	0.001	0.983	0.007	0.935	0.001	0.978	0.004	0.935	-0.001	0.983
lh_pericalcarine_Vol	0.004	0.919	0.012	0.749	0.012	0.562	0.007	0.829	0.009	0.706
rh_pericalcarine_Vol	0.005	0.841	-0.001	0.983	0.008	0.749	0.012	0.503	0.011	0.578
lh_postcentral_Vol	-0.016	0.15	-0.006	0.96	-0.004	0.935	-0.004	0.935	-0.003	0.95
rh_postcentral_Vol	-0.014	0.265	-0.011	0.81	-0.004	0.935	-0.004	0.935	-0.003	0.948
lh_posteriorcingulate_Vol	-0.021	**0.044**	-0.006	0.975	-0.017	0.2	-0.016	0.237	-0.018	0.13
rh_posteriorcingulate_Vol	-0.013	0.329	0.011	0.829	-0.008	0.749	-0.007	0.81	-0.008	0.749
lh_precentral_Vol	-0.030	**0.001**	-0.007	0.935	-0.019	0.105	-0.019	0.109	-0.02	0.086
rh_precentral_Vol	-0.027	**0.004**	-0.021	0.208	-0.016	0.211	-0.016	0.222	-0.015	0.291
lh_precuneus_Vol	-0.022	**0.032**	-0.005	0.975	-0.007	0.829	-0.005	0.89	-0.005	0.928
rh_precuneus_Vol	-0.023	**0.016**	-0.016	0.565	-0.011	0.565	-0.011	0.581	-0.012	0.488
lh_rostralanteriorcingulate_Vol	-0.024	**0.013**	-0.005	0.975	-0.017	0.188	-0.017	0.208	-0.016	0.199
rh_rostralanteriorcingulate_Vol	-0.017	0.145	0.004	0.975	-0.011	0.565	-0.014	0.347	-0.012	0.533
lh_rostralmiddlefrontal_Vol	-0.005	0.848	0.008	0.935	0.005	0.919	0.005	0.919	0.004	0.935
rh_rostralmiddlefrontal_Vol	-0.018	0.11	-0.009	0.919	-0.011	0.572	-0.014	0.37	-0.012	0.506
lh_superiorfrontal_Vol	-0.014	0.265	0.003	0.978	0.001	0.983	0.002	0.978	0.002	0.975

rh_superiorfrontal_Vol	-0.009	0.61	0.009	0.901	0.003	0.950	0.003	0.96	0.003	0.950
lh_superiorparietal_Vol	-0.009	0.59	0.019	0.295	0.003	0.950	0.004	0.935	0.002	0.978
rh_superiorparietal_Vol	-0.019	0.082	-0.003	0.978	-0.006	0.841	-0.005	0.89	-0.007	0.829
lh_superiortemporal_Vol	-0.016	0.145	0.002	0.978	-0.005	0.919	-0.005	0.919	-0.004	0.935
rh_superiortemporal_Vol	-0.018	0.101	-0.001	0.983	-0.007	0.829	-0.007	0.829	-0.008	0.790
lh_supramarginal_Vol	-0.015	0.214	0.007	0.935	-0.006	0.841	-0.007	0.829	-0.006	0.900
rh_supramarginal_Vol	-0.012	0.340	0.009	0.888	-0.004	0.935	-0.003	0.960	-0.004	0.935
lh_transversetemporal_Vol	-0.008	0.620	-0.01	0.841	-0.004	0.935	-0.004	0.935	-0.004	0.935
rh_transversetemporal_Vol	-0.01	0.524	-0.006	0.950	-0.005	0.919	-0.005	0.929	-0.006	0.900
wholeBrainWMHvol	0.094	**<0.001**	-0.001	0.983	0.072	**<0.001**	0.074	**<0.001**	0.087	**<0.001**
PVWMHvol	0.094	**<0.001**	0.016	0.565	0.073	**<0.001**	0.075	**<0.001**	0.086	**<0.001**
DWMHvol	0.077	**<0.001**	-0.006	0.975	0.055	**<0.001**	0.05	**<0.001**	0.058	**<0.001**
Lfrontal_WMHvol	0.075	**<0.001**	-0.01	0.841	0.045	**<0.001**	-0.035	**<0.001**	0.066	**<0.001**
Rfrontal_WMHvol	0.079	**<0.001**	-0.012	0.749	0.050	**<0.001**	-0.028	**0.004**	0.068	**<0.001**
Ltemporal_WMHvol	0.041	**<0.001**	0.011	0.807	0.052	**<0.001**	-0.012	0.503	0.068	**<0.001**
Rtemporal_WMHvol	0.028	**0.002**	-0.002	0.978	0.055	**<0.001**	-0.014	0.37	0.065	**<0.001**
Lparietal_WMHvol	0.088	**<0.001**	-0.001	0.983	0.054	**<0.001**	-0.031	**0.001**	0.07	**<0.001**
Rparietal_WMHvol	0.092	**<0.001**	0.026	0.065	0.065	**<0.001**	-0.012	0.497	0.077	**<0.001**
Loccipital_WMHvol	0.015	0.191	-0.015	0.578	0.026	**0.009**	-0.010	0.621	0.038	**<0.001**
Roccipital_WMHvol	0.034	**<0.001**	-0.003	0.978	0.035	**<0.001**	0	0.986	0.041	**<0.001**

Lcerebellum_WMHvol	0.025	**0.007**	0.021	0.208	-0.031	**0.001**	-0.030	**0.001**	0.020	0.085
Rcerebellum_WMHvol	0.032	**<0.001**	0.001	0.978	0.009	0.691	-0.011	0.585	-0.009	0.682
Brainstem_WMHvol	0.014	0.226	-0.007	0.935	0.014	0.377	-0.042	**<0.001**	0.016	0.208
lAAH_WMHvol	0.076	**<0.001**	0.004	0.975	0.045	**<0.001**	0.015	0.293	0.058	**<0.001**
rAAH_WMHvol	0.075	**<0.001**	0.004	0.975	0.042	**<0.001**	0.025	**0.019**	0.051	**<0.001**
lMAH_WMHvol	0.078	**<0.001**	-0.015	0.615	0.049	**<0.001**	0.054	**<0.001**	0.052	**<0.001**
rMAH_WMHvol	0.089	**<0.001**	0.009	0.919	0.068	**<0.001**	0.067	**<0.001**	0.066	**<0.001**
lAAML_WMHvol	0.079	**<0.001**	-0.004	0.975	0.057	**<0.001**	0.058	**<0.001**	0.062	**<0.001**
rAAML_WMHvol	0.076	**<0.001**	-0.007	0.935	0.052	**<0.001**	0.051	**<0.001**	0.056	**<0.001**
lAAC_WMHvol	0.081	**<0.001**	0.004	0.975	0.063	**<0.001**	0.064	**<0.001**	0.063	**<0.001**
rAAC_WMHvol	0.062	**<0.001**	-0.016	0.565	0.053	**<0.001**	0.059	**<0.001**	0.044	**<0.001**
lMALL_WMHvol	0.090	**<0.001**	0.011	0.81	0.068	**<0.001**	0.072	**<0.001**	0.072	**<0.001**
rMALL_WMHvol	0.092	**<0.001**	0.017	0.424	0.073	**<0.001**	0.076	**<0.001**	0.080	**<0.001**
lPATMP_WMHvol	0.032	**<0.001**	0.005	0.975	0.026	**0.007**	0.026	**0.01**	0.022	0.058
rPATMP_WMHvol	0.037	**<0.001**	-0.005	0.975	0.034	**<0.001**	0.032	**0.001**	0.025	**0.013**
lPAH_WMHvol	0.067	**<0.001**	0.003	0.978	0.041	**<0.001**	0.017	0.199	0.048	**<0.001**
rPAH_WMHvol	0.066	**<0.001**	0.003	0.978	0.056	**<0.001**	0.030	**0.001**	0.058	**<0.001**
lPAC_WMHvol	-0.036	**<0.001**	0.048	**<0.001**	0.051	**<0.001**	-0.045	**<0.001**	0.062	**<0.001**
rPAC_WMHvol	0.025	**0.009**	0.028	0.034	0.037	**<0.001**	-0.080	**<0.001**	0.045	**<0.001**

This table presents Spearman's correlation coefficients and corresponding FDR-corrected p-values for the relationship between White Matter Hyperintensity (WMH) volume Z-scores and hypertension levels (0-3) for different brain regions. Results are shown for five different modelling approaches: prior-sampling cVAE, posterior-sampling cVAE, GAMLSS, MFPR, and HBR. Abbreviations: cVAE = conditional Variational Autoencoder; GAMLSS = Generalised Additive Models for Location, Scale and Shape; MFPR = Multivariate Fractional Polynomial Regression; HBR = Hierarchical Bayesian Regression. See Table S1 for abbreviations of lobar and arterial regions.

Table S9 Percentage of positive extreme deviations (%) of selected brain measures across models.

	Prior-cVAE				Posterior- cVAE				GAMLSS				MFPR				HBR			
**Hypertension Level**	**0**	**1**	**2**	**3**	**0**	**1**	**2**	**3**	**0**	**1**	**2**	**3**	**0**	**1**	**2**	**3**	**0**	**1**	**2**	**3**
Mean thickness of the left insula	0.58	0.46	0.48	0.78	0.35	0.36	0.40	0.28	0.53	0.46	0.39	0.57	0.29	0.32	0.31	0.50	0.35	0.47	0.53	0.71
Mean thickness of the right insula	0.64	0.46	0.40	0.28	0.64	0.50	0.58	0.28	0.47	0.55	0.45	0.28	0.41	0.27	0.34	0.28	0.47	0.41	0.51	0.43
Volume of whole-brain subcortical grey matter volume	1.22	1.00	1.03	0.71	0.23	0.21	0.32	0.14	1.87	1.56	1.69	1.21	0.88	0.70	0.90	0.50	1.05	1.05	1.11	0.71
Volume of the 5th ventricle	1.75	2.16	2.06	2.27	0.29	0.34	0.37	0.28	56.52	56.34	55.18	52.80	1.75	2.16	2.06	2.27	1.75	2.16	2.06	2.27
Volume of whole-brain cortical grey matter	0.70	1.01	0.69	0.78	0.23	0.50	0.48	0.28	1.69	1.54	1.35	1.14	0.41	0.64	0.59	0.64	0.64	0.66	0.66	0.85
Volume of whole-brain WMH	2.28	5.56	7.26	10.93	0.12	0.31	0.27	0.35	3.16	5.83	7.54	10.01	0.70	1.46	1.38	3.26	1.17	2.74	3.41	5.75
WMH volume of the left cerebellum	7.25	6.48	7.04	6.53	0.06	0.16	0.21	0.21	51.96	53.79	54.31	54.08	8.30	7.53	8.07	7.59	8.30	7.53	8.07	7.59
WMH volume of the right cerebellum	7.13	7.03	6.59	5.96	0.23	0.14	0.26	0.21	58.56	56.66	56.47	53.37	7.42	7.27	6.89	6.10	7.42	7.27	6.89	6.10
WMH volume of the left posterior artery callosal	12.68	15.10	15.06	18.10	0.12	0.17	0.08	0	53.19	56.26	58.07	62.31	0	0.02	0.05	0	19.40	25.26	28.15	35.70
WMH volume of the right posterior artery callosal	7.36	7.68	8.48	11.07	0.06	0.08	0.05	0	66.69	68.58	70.69	77.93	1.17	1.52	1.90	2.63	12.62	16.29	19.07	24.63

This table presents the percentage of samples with positive extreme deviations (z-score > 2.58) of selected brain features across hypertension levels (0-3) for five different modelling approaches: prior- cVAE, posterior- cVAE, GAMLSS, MFPR, and HBR. Abbreviations: cVAE = conditional Variational Autoencoder;

GAMLSS = Generalised Additive Models for Location, Scale and Shape; MFPR = Multivariate Fractional Polynomial Regression; HBR = Hierarchical Bayesian Regression.

Table S10 Percentage of negative extreme deviations (%) of selected brain measures across models.

	Prior-cVAE				Posterior-cVAE				GAMLSS				MFPR				HBR			
**Hypertension Level**	**0**	**1**	**2**	**3**	**0**	**1**	**2**	**3**	**0**	**1**	**2**	**3**	**0**	**1**	**2**	**3**	**0**	**1**	**2**	**3**
Mean thickness of the left insula	0.70	1.55	1.72	3.34	0.41	0.71	0.71	1.14	1.11	1.90	1.91	3.34	0.41	0.79	0.93	2.13	0.64	1.21	1.41	2.98
Mean thickness of the right insula	0.58	1.29	1.43	1.63	0.53	0.72	0.72	0.71	1.17	2.20	2.39	2.34	0.41	0.84	0.90	1.28	0.58	1.38	1.41	1.77
Volume of whole-brain subcortical grey matter volume	0.29	0.92	0.80	1.35	0.06	0.13	0.05	0	0.23	0.86	0.66	1.14	0.12	0.49	0.39	0.64	0.12	0.66	0.53	1.21
Volume of the 5th ventricle	0	0	0	0	0	0.038	0.016	0	42.90	43.33	44.40	46.91	0	0	0	0	0	0	0	0
Volume of whole-brain cortical grey matter	0.94	1.35	1.37	1.35	0.41	0.69	0.55	0.50	1.23	1.62	1.66	1.35	0.47	0.68	0.98	0.78	0.58	0.85	1.01	0.64
Volume of whole-brain WMH	0.53	0.77	0.63	0.99	0.11	0.03	0.02	0.07	0	0.11	0.10	0.07	0.06	0.05	0.08	0.07	0.06	0.21	0.22	0.35
WMH volume of the left cerebellum	0	0	0	0	0	0	0	0	47.69	45.83	45.09	45.42	0	0	0	0	0	0	0	0
WMH volume of the right cerebellum	0	0	0	0	0	0.01	0	0	41.09	42.64	42.83	45.85	0	0	0	0	0	0	0	0
WMH volume of the left posterior artery callosal	0	0	0	0	0	0	0	0	46.52	43.52	41.57	37.33	0	0	0	0	0	0	0	0
WMH volume of the right posterior artery callosal	0	0	0	0	0	0	0	0	33.08	31.16	29.18	21.86	0	0	0	0	0	0	0	0

This table presents the percentage of samples with negative extreme deviations (z-score < -2.58) of selected brain features across hypertension levels (0-3) for five different modelling approaches: prior-cVAE, posterior-cVAE, GAMLSS, MFPR, and HBR. Abbreviations: cVAE = conditional Variational Autoencoder;

GAMLSS = Generalised Additive Models for Location, Scale and Shape; MFPR = Multivariate Fractional Polynomial Regression; HBR = Hierarchical Bayesian Regression.

Supplementary Figures

Figure S1 Desikan-Killiany-Tourville (DKT) and Automated Subcortical Segmentation (ASEG) Atlases

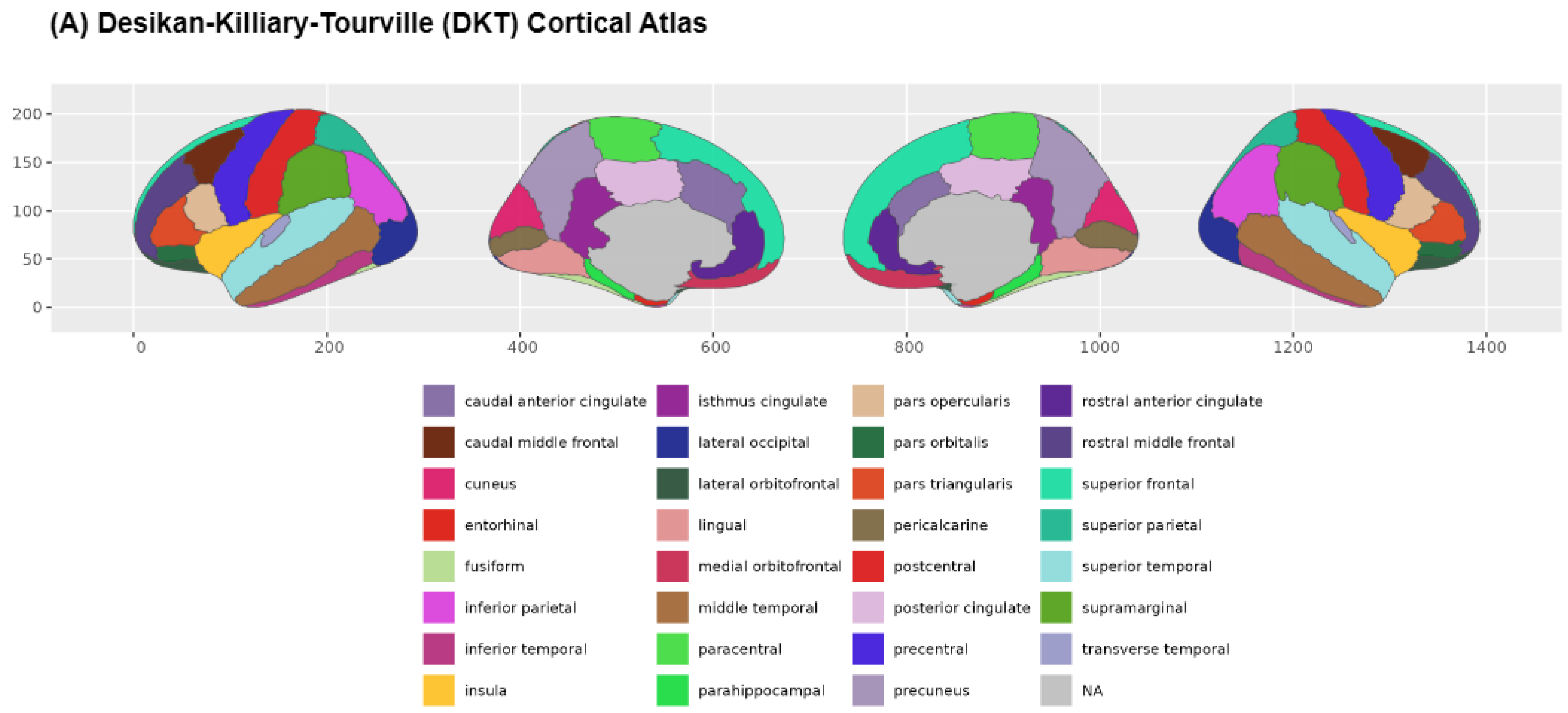

(B) FreeSurfer Automated Subcortical Segmentation (ASEG) Atlas

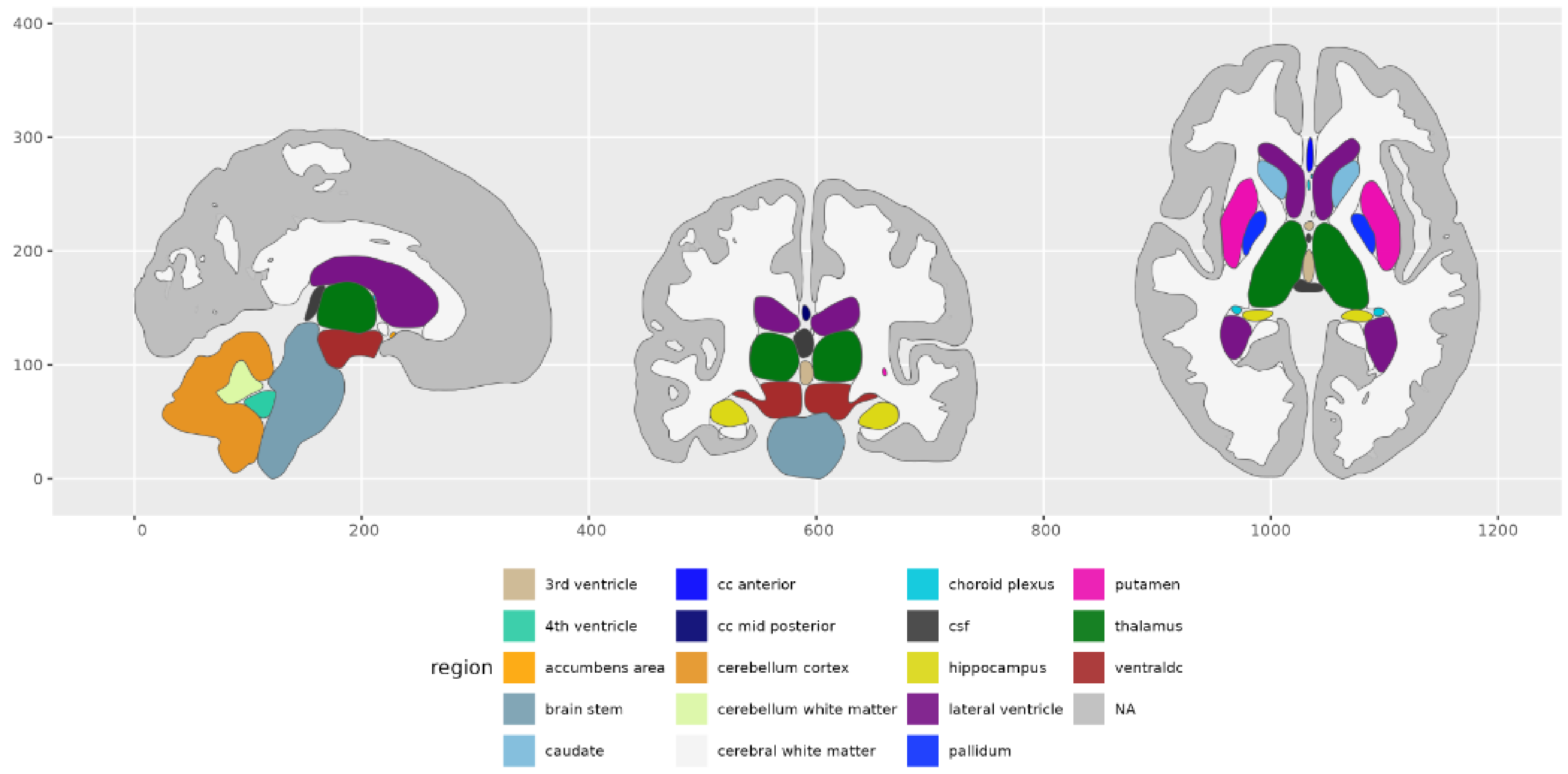

Note: Some subcortical structures are not visible in this visualisation due to their small size and position relative to the displayed brain slices, including: 5th ventricle, thalamus, amygdala, CC central, CC mid anterior, CC posterior, and Inferior Lateral Ventricle (Inf-Lat-Vent).

Abbreviations: CC = corpus callosum; CSF = Cerebrospinal Fluid

Figure S2 Posterior Sampling Inference Approach in Conventional cVAE-based Normative Modelling

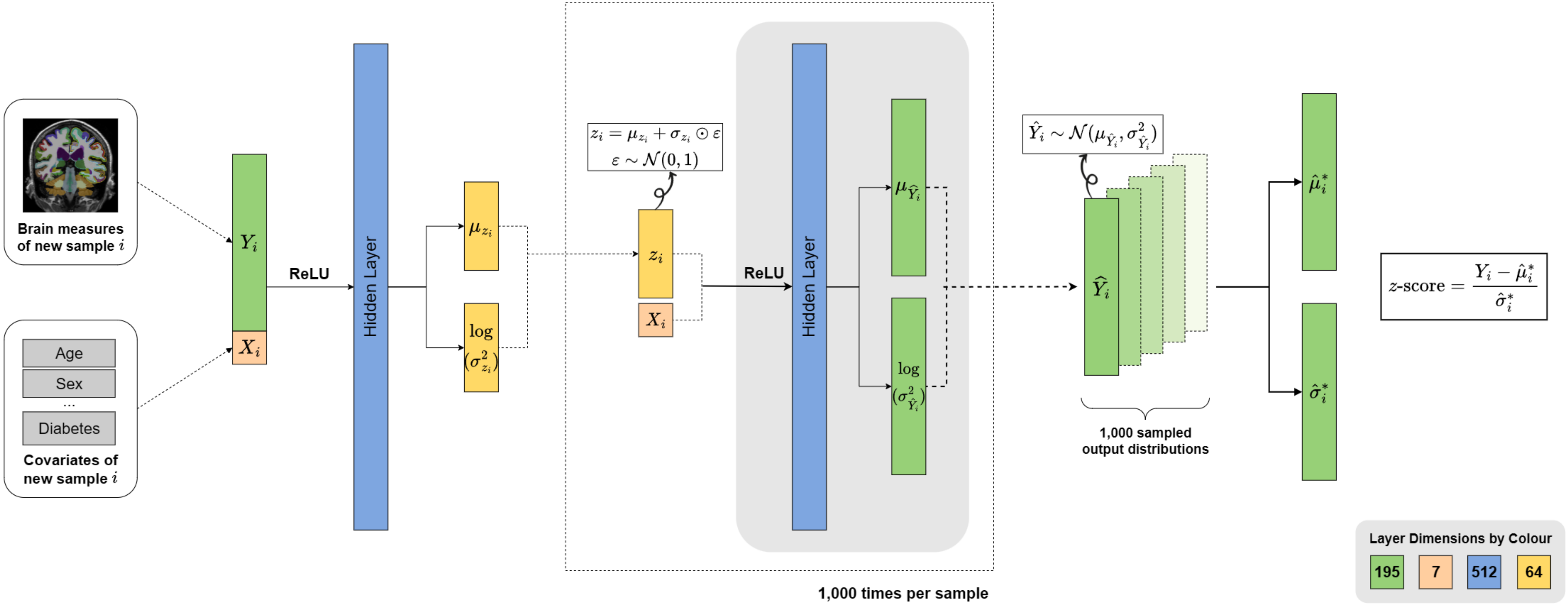

This illustration shows the posterior-sampling inference approach used in existing cVAE-based normative models, which differs fundamentally from our proposed prior sampling method. In conventional posterior sampling, both observed WMH volumes (Y_i) and covariates (X_i) of each sample are encoded into

latent space parameters (μ_{z_i} and σ_{z_i}), from which latent vectors (z_i) are sampled. These vectors, combined with covariates, are then decoded to generate parameters forming a multivariate normal distribution $\widehat{Y}_i \sim \mathcal{N}\left(\mu_{\widehat{Y}_i}, \sigma^2_{\widehat{Y}_i}\right)$. For consistency with our proposed inference approach, we have implemented the same sampling procedures. Specifically, for each of the 1,000 sampling rounds, a latent vector (z_i) is first sampled using the reparameterization trick, then passed through the decoder along with covariates to produce parameters of a multivariate normal distribution $\mathcal{N}\left(\mu_{\widehat{Y}_i}, \sigma^2_{\widehat{Y}_i}\right)$. The final probabilistic estimates ($\hat{\mu}_i^*$ and $\hat{\sigma}_i^*$) are derived from these 1,000 sampled output distributions.

Layers represented in the same colour indicate identical dimensions. Abbreviations: ReLU = Rectified Linear Unit. $\odot$ denotes element-wise multiplication.

Figure S3 Heatmaps of Spearman Correlations Between Covariates and Brain Region-Specific Measures in the Training Dataset.

(A) WMH volumetric measures:

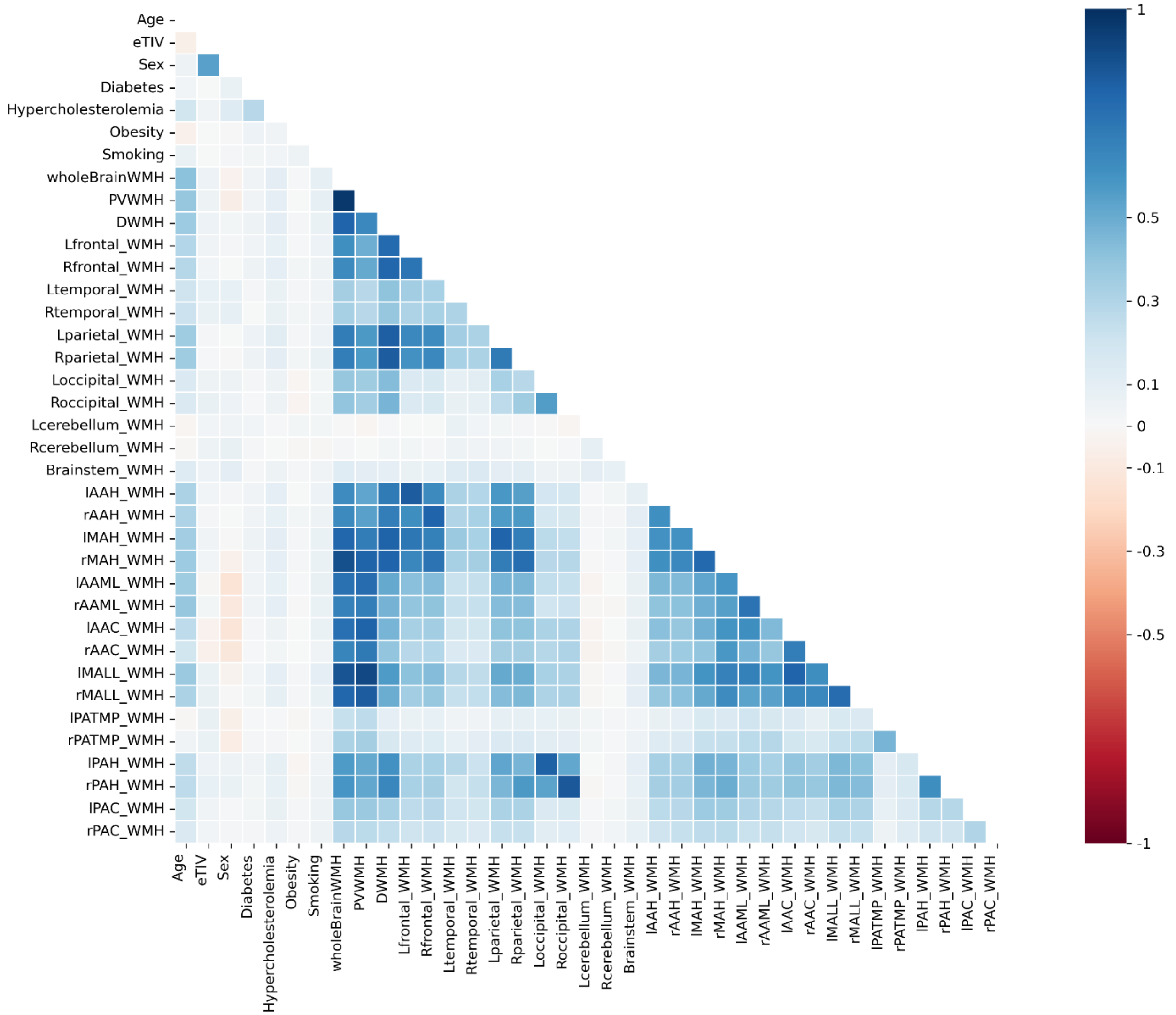

(B) Cortical mean thickness measures:

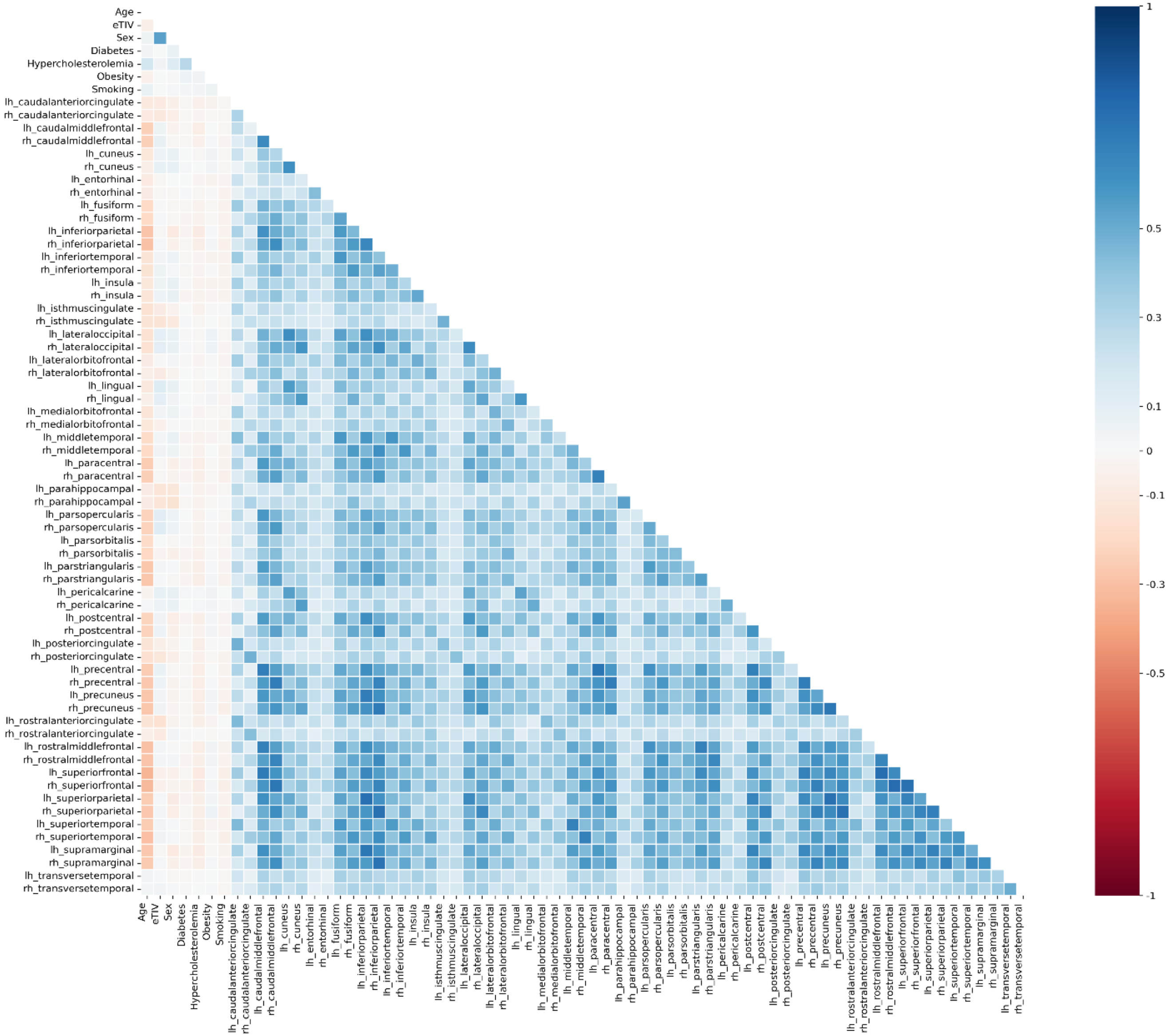

(C) Cortical volumetric measures:

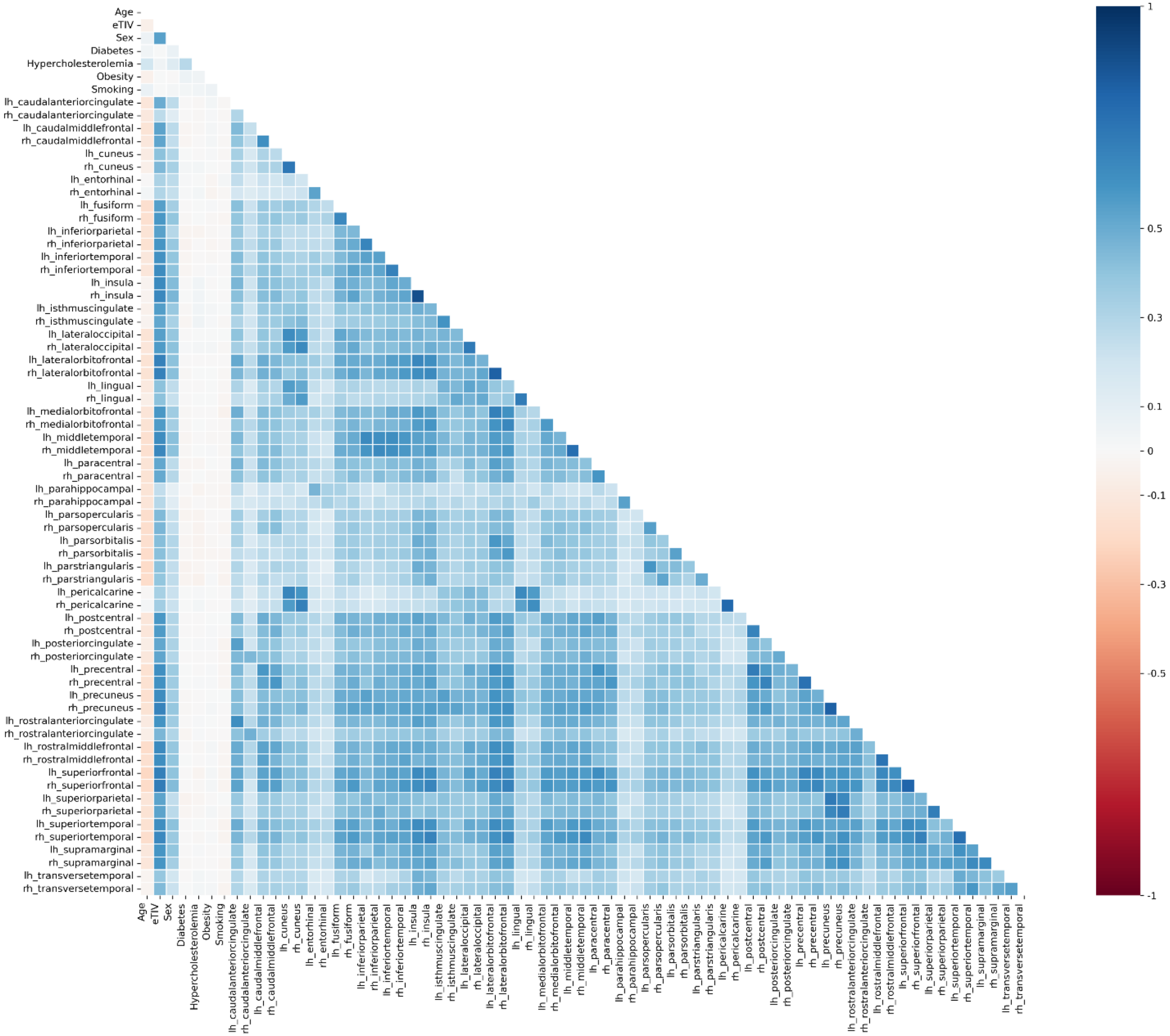

(D) Subcortical volumetric measures:

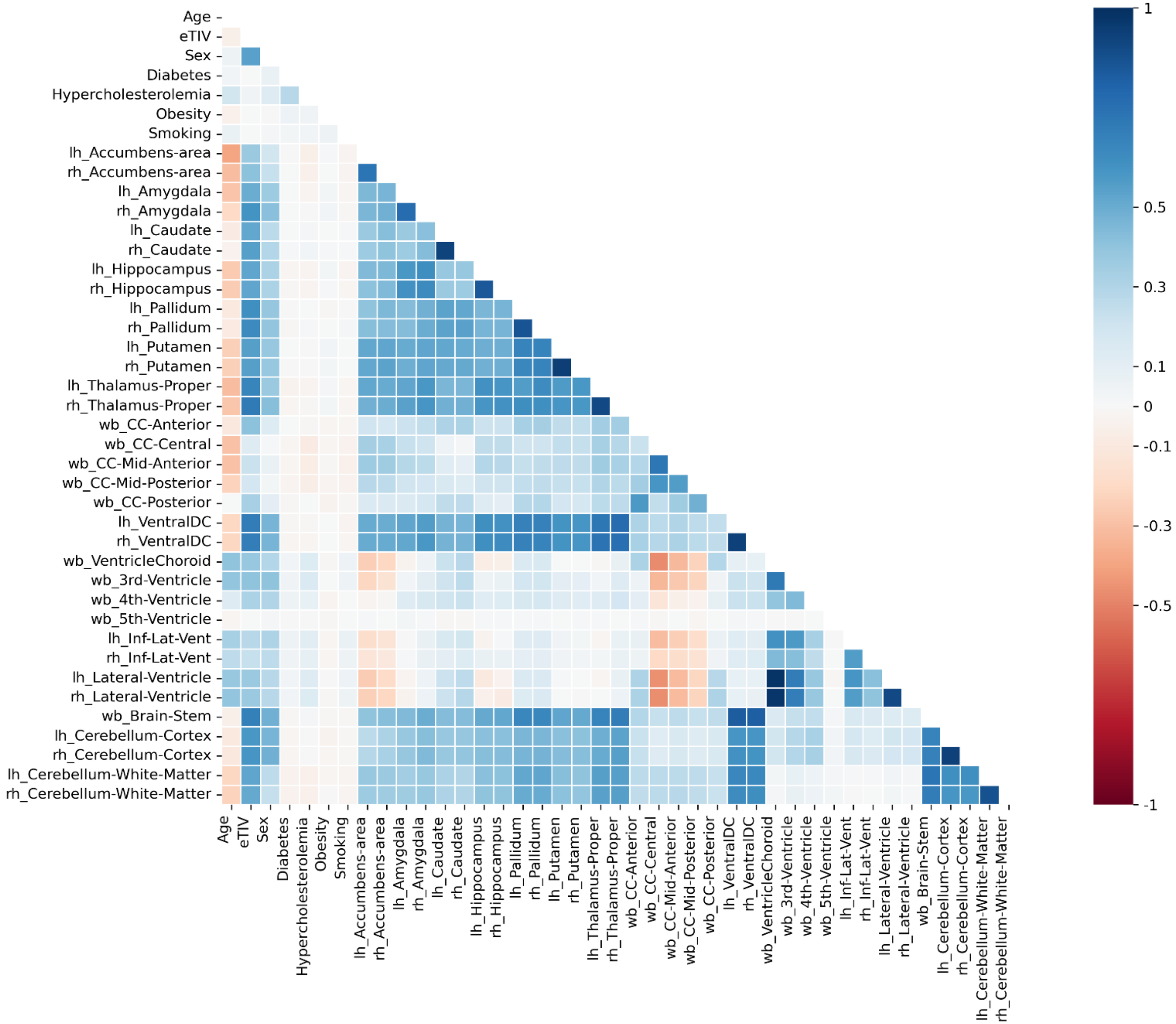

Figure S4 Bland-Altman Plots of Root Mean Squared Error for prior-cVAE vs Other Models.

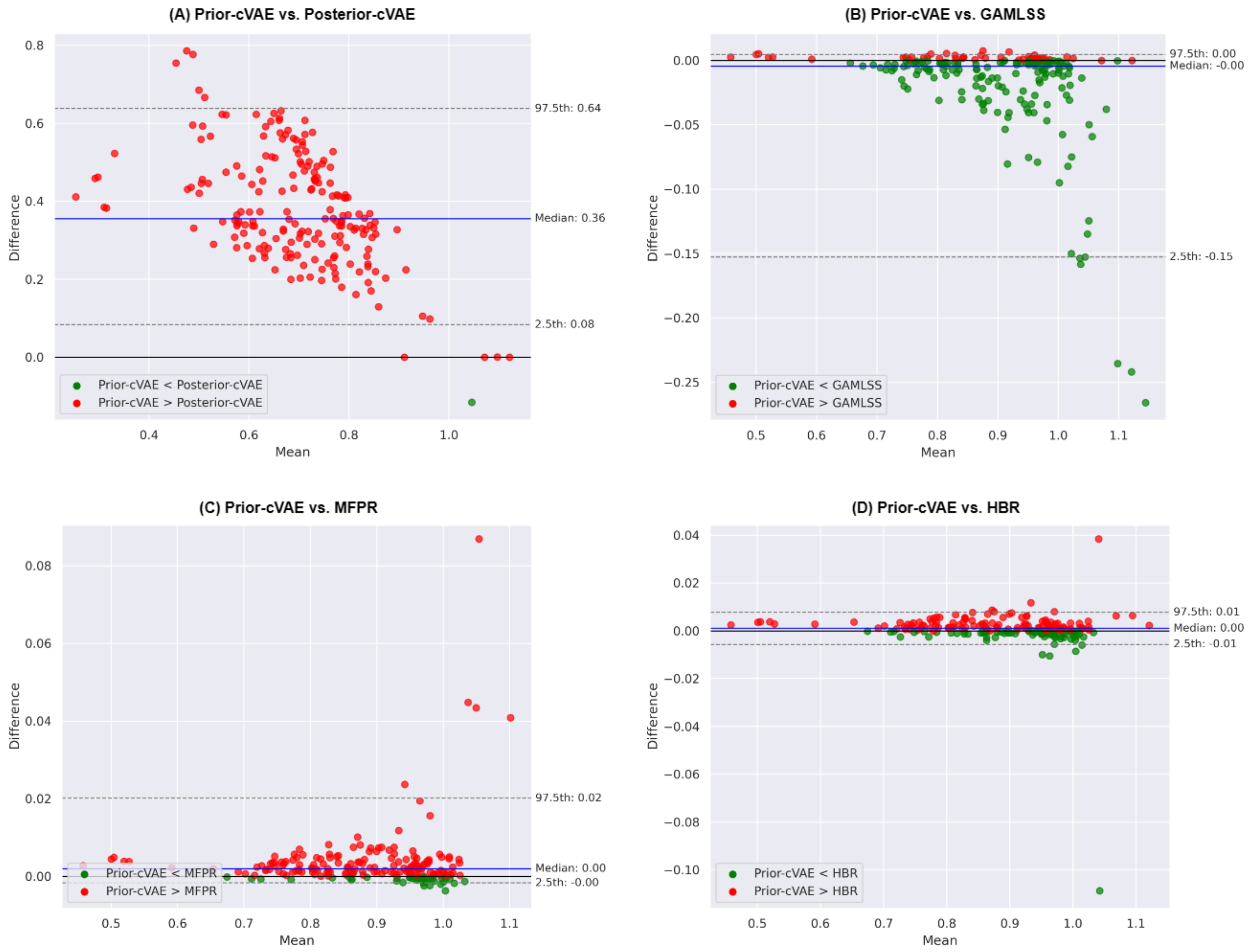

Bland-Altman plots comparing the Root Mean Squared Error (RMSE) of the prior-sampling conditional Variational Autoencoder (cVAE) model against the posterior-sampling cVAE (A), Generalised Additive Models for Location, Scale, and Shape (GAMLSS) (B), Multivariate Fractional Polynomial Regression (MFPR) (C), and Hierarchical Bayesian Regression (HBR) (D) models. Each point represents a different brain region. Green points indicate regions where cVAE exhibits lower RMSE compared to the corresponding model, while red points show regions where the other model performs better.

Figure S5 Bland-Altman Plots of Spearman Correlation for prior-cVAE vs Other Models.

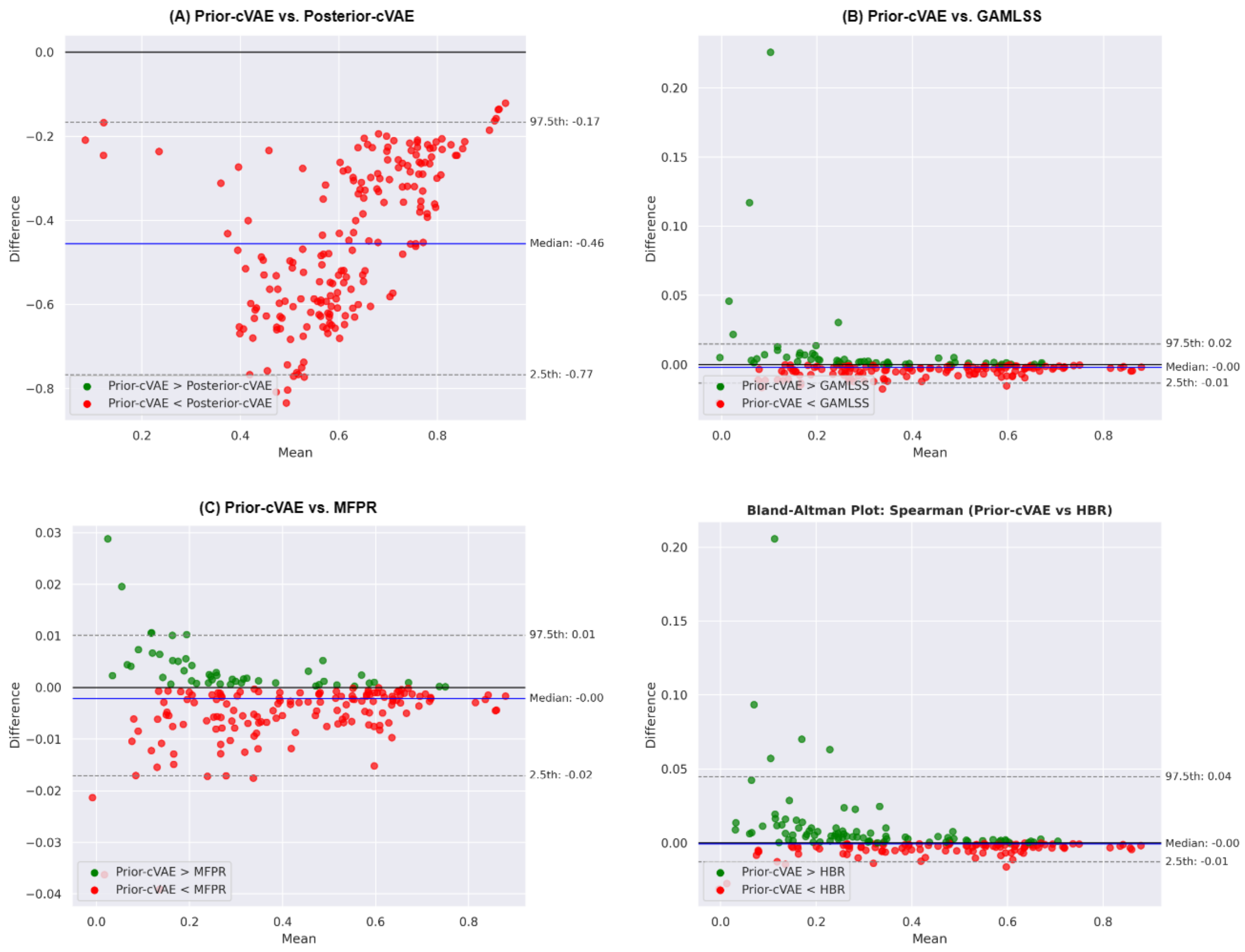

Bland-Altman plots comparing the Spearman Correlation of the prior-sampling conditional Variational Autoencoder (cVAE) model against the posterior-sampling cVAE (A), Generalised Additive Models for Location, Scale, and Shape (GAMLSS) (B), Multivariate Fractional Polynomial Regression (MFPR) (C), and Hierarchical Bayesian Regression (HBR) (D) models. Each point represents a different brain region. Green points indicate regions where cVAE exhibits higher Spearman correlation than the corresponding model, while red points show regions where the other model performs better.

Figure S6 Comparison of Z-Scores for Whole-Brain Grey Matter Volume across Hypertensive Levels and Models.

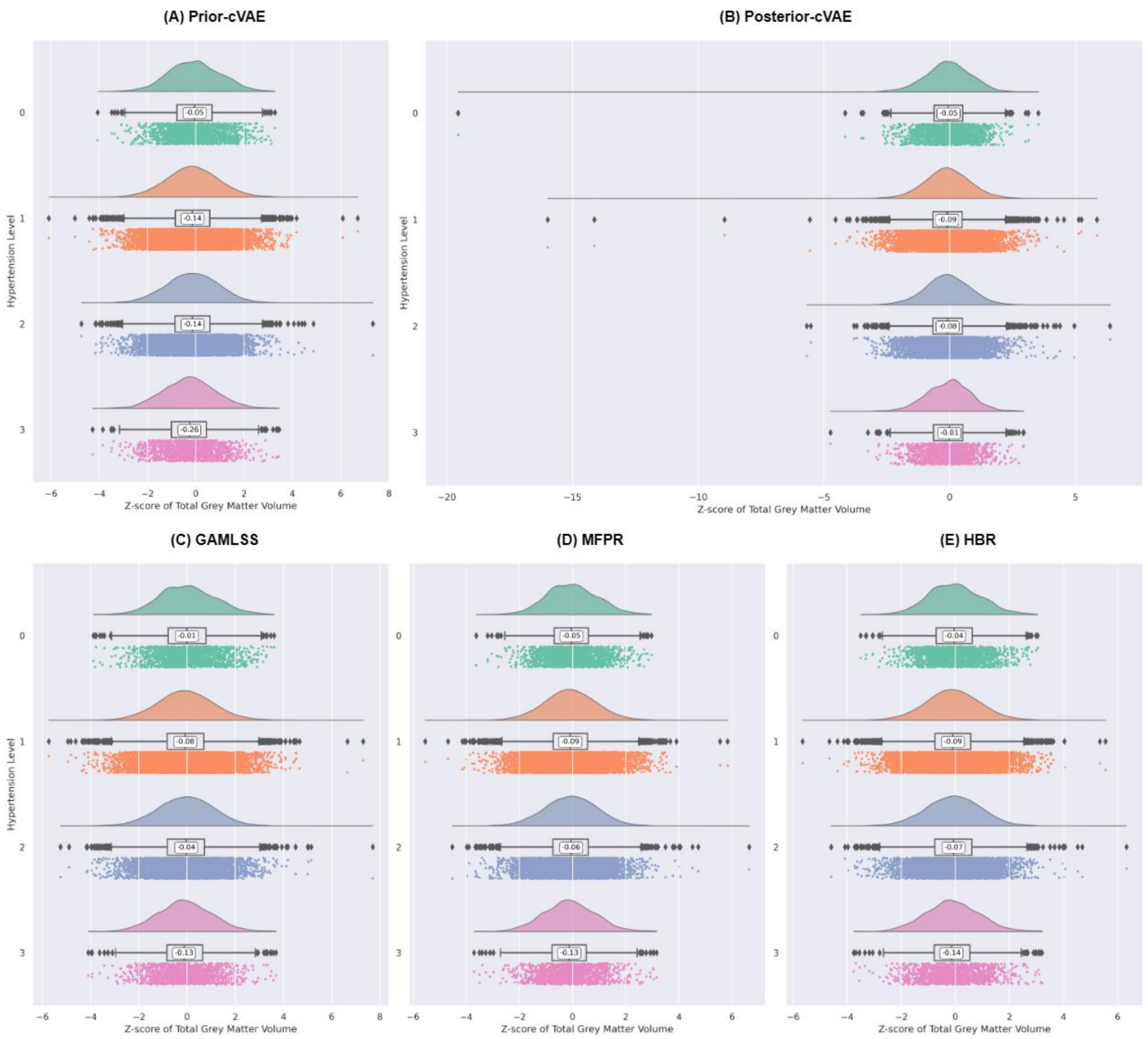

This figure displays the distribution of z-scores for whole-brain grey matter volume stratified by hypertensive levels (0-3) across five different modelling approaches. Each panel corresponds to a different model: (A) Prior-sampling conditional Variational Autoencoder (prior-cVAE), our proposed inference approach that generates brain measures directly from covariates; (B) Posterior-sampling cVAE, the conventional inference approach that uses both observed data and covariates (posterior-cVAE); (C) Generalised Additive Models for Location, Scale and Shape (GAMLSS); (D) Multivariate Fractional Polynomial Regression (MFPR); and (E) Hierarchical Bayesian Regression (HBR).

Figure S7 Spearman Correlation between Z-score and Hypertension levels.

The below bubble chart illustrates the Spearman correlation between z-scores and hypertension levels (0-3) in the evaluation dataset, comparing different models and inference approaches. The chart includes both our proposed prior-sampling cVAE approach and the conventional posterior-sampling cVAE approach, alongside GAMLSS, MFPR, and HBR models across various brain regions. Bubble size and colour intensity represent correlation strength, with darker blue indicating stronger positive correlations and darker red showing stronger negative correlations. Bubbles with borders denote statistically significant correlations (False Discovery Rate (FDR)-corrected p-value < 0.05). Abbreviations: cVAE = conditional Variational Autoencoder; GAMLSS = Generalised Additive Models for Location, Scale and Shape; MFPR = Multivariate Fractional Polynomial Regression; HBR = Hierarchical Bayesian Regression; WMH = White Matter Hyperintensity. See Table S1 for brain region-specific measure abbreviations.

(A) **WMH volumetric measures:**

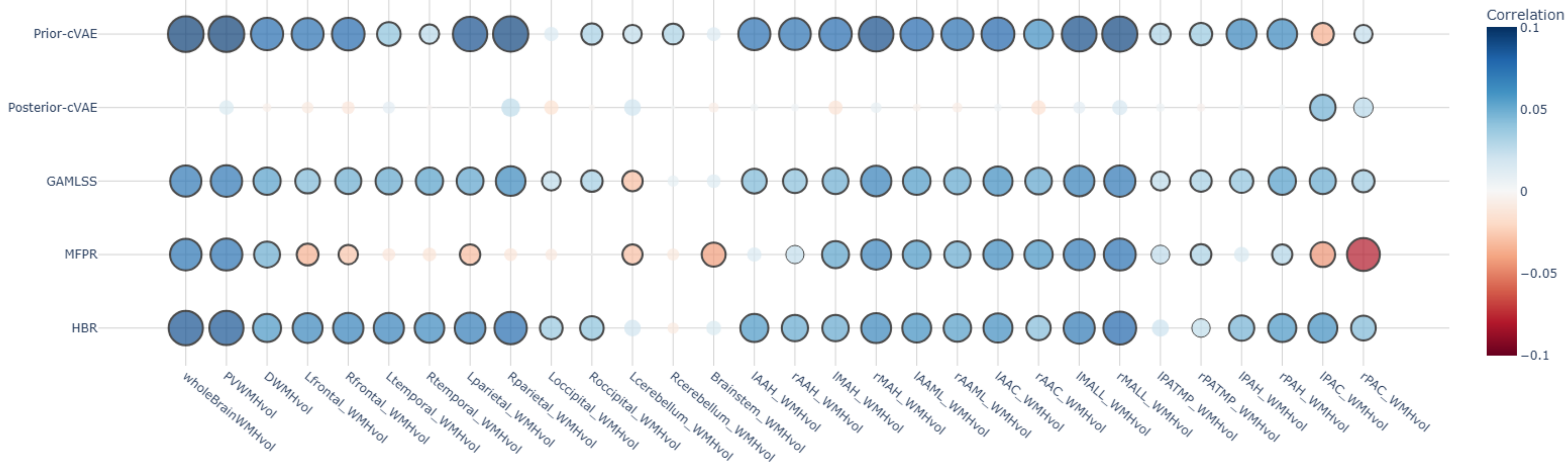

(B) **Cortical mean thickness measures:**

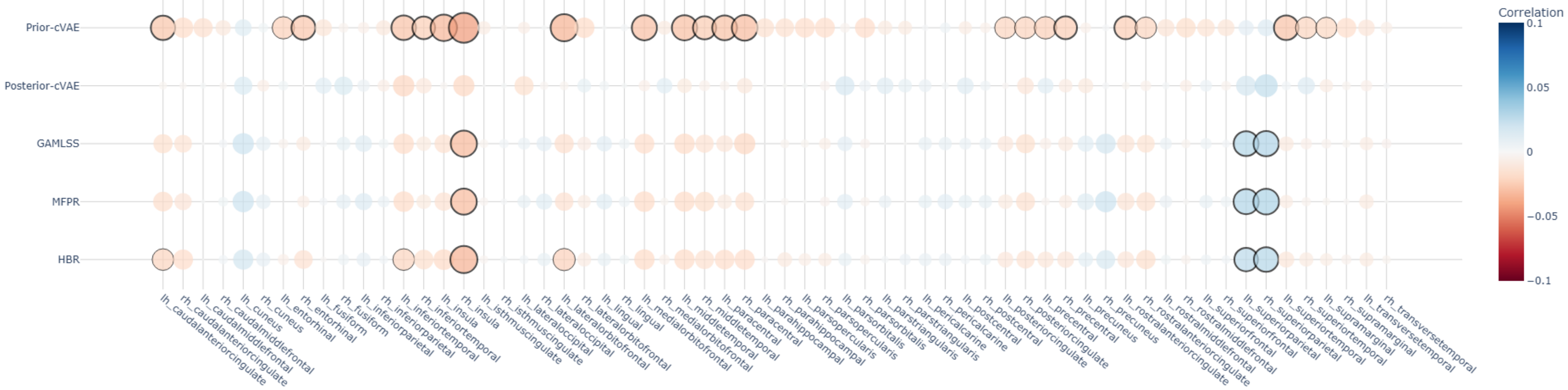

(C) **Cortical volumetric measures:**

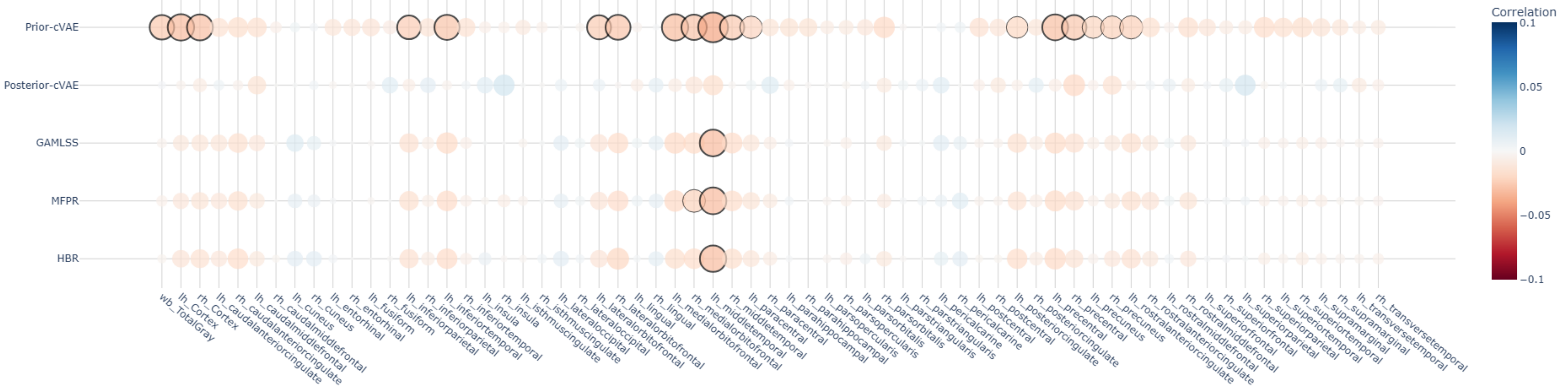

(D) **Subcortical volumetric measures:**

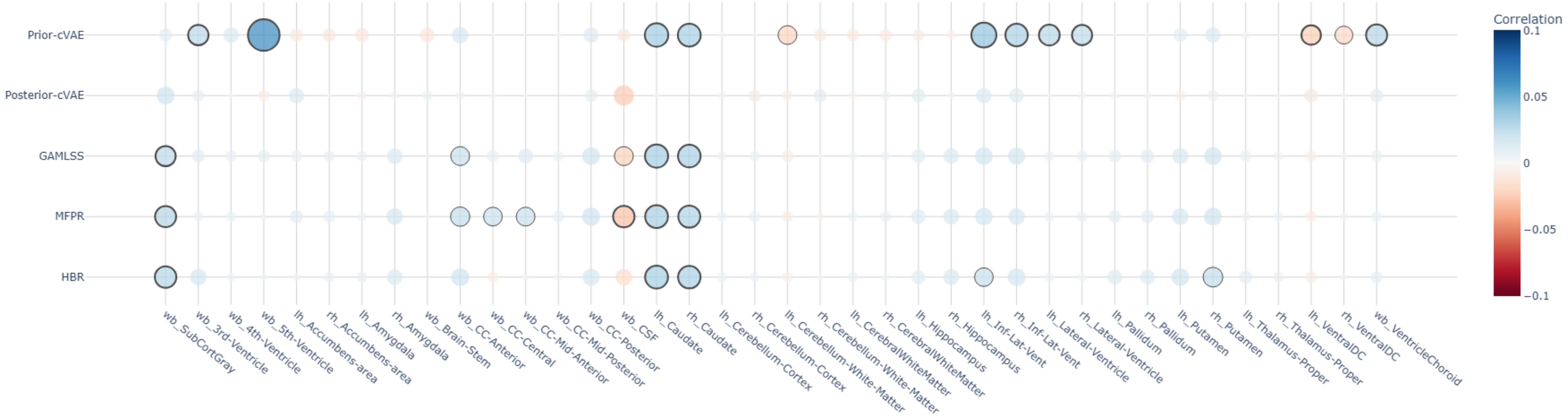

Figure S8 Comparison of Positive Extreme Deviations across Models and Hypertension Levels.

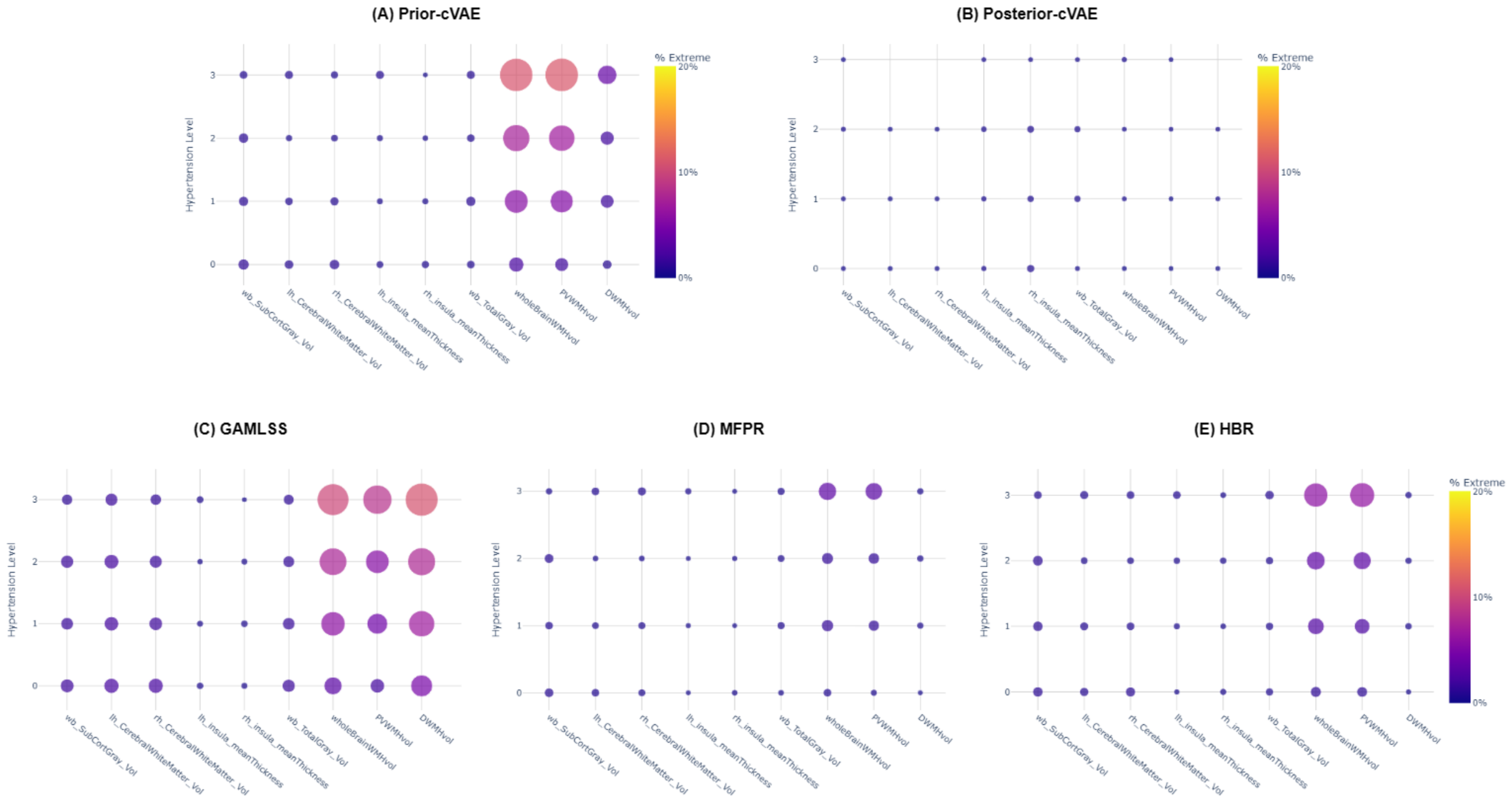

This bubble chart shows the percentage of samples with positive extreme deviations (z-score > 2.58) across hypertension levels (0-3) for 5 different modelling approaches: (A) Prior-sampling conditional Variational Autoencoder (cVAE), our proposed inference approach; (B) Posterior-sampling cVAE, the conventional inference approach; (C) Generalised Additive Models for Location, Scale and Shape (GAMLSS); (D) Multivariate Fractional Polynomial Regression (MFPR); and (E) Hierarchical Bayesian Regression (HBR). Bubble size and colour intensity indicate the percentage of extreme deviations, with larger, brighter bubbles representing higher percentages. Abbreviations: wholeBrainWMHvol = whole-brain WMH volume; PVWMHvol = periventricular WMH volume; DWMHvol = deep white matter hyperintensity volume.

Figure S9 Comparison of Negative Extreme Deviations across Models and Hypertension Levels.

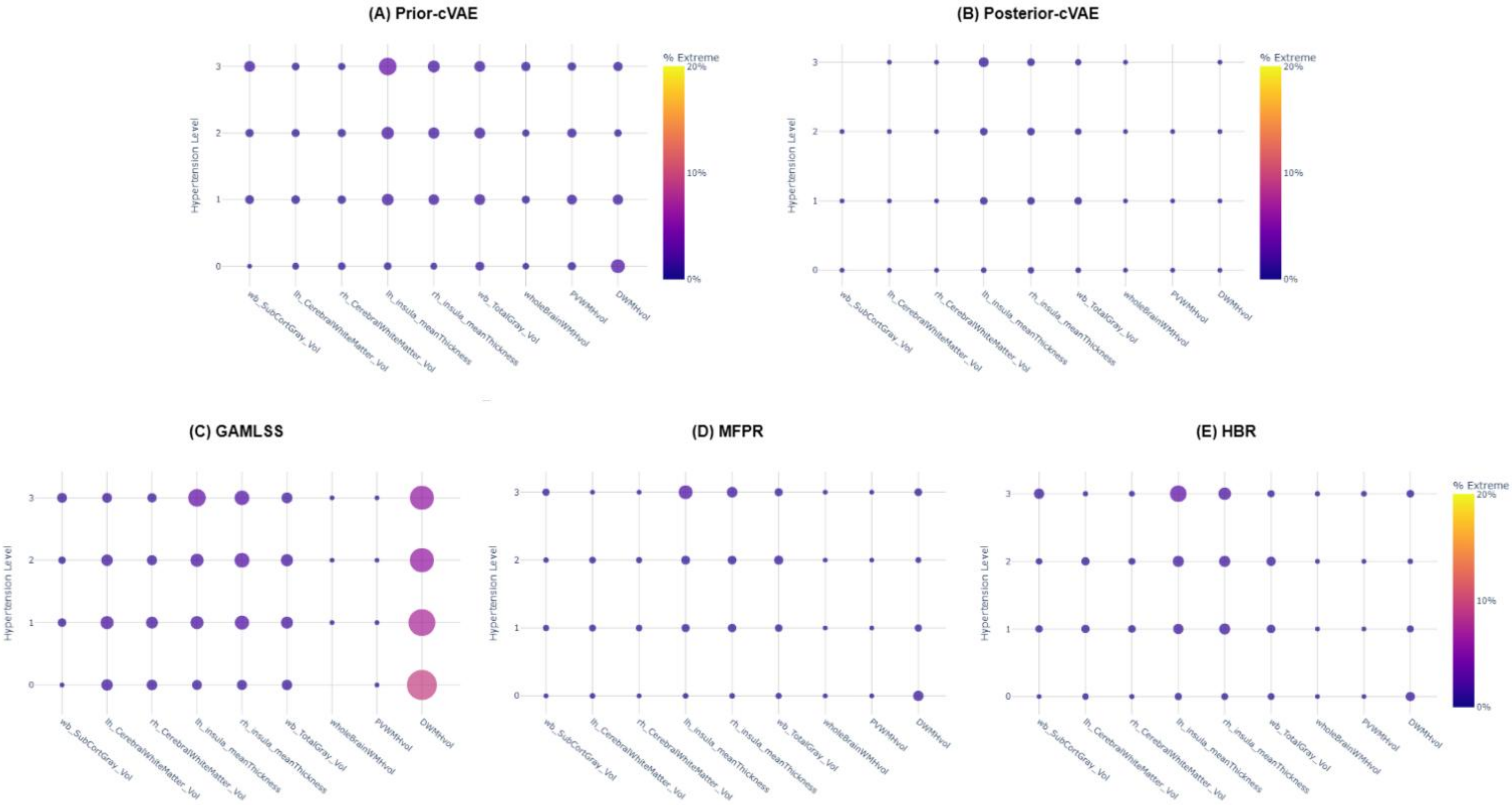

This bubble chart shows the percentage of samples with negative extreme deviations (z-score < 2.58) across hypertension levels (0-3) for 5 different modelling approaches: (A) Prior-sampling conditional Variational Autoencoder (cVAE), our proposed inference approach; (B) Posterior-sampling cVAE, the conventional inference approach; (C) Generalised Additive Models for Location, Scale and Shape (GAMLSS); (D) Multivariate Fractional Polynomial Regression (MFPR); and (E) Hierarchical Bayesian Regression (HBR). Bubble size and colour intensity indicate the percentage of extreme deviations, with larger, brighter bubbles representing higher percentages. Abbreviations: wholeBrainWMHvol = whole-brain WMH volume; PVWMHvol = periventricular WMH volume; DWMHvol = deep white matter hyperintensity volume.

Supplementary Methods

S1 UBO Detector Pipeline

The UBO (Unidentified Bright Object) Detector pipeline (Jiang et al., 2018) used in this study is a fully automated tool for extracting and quantifying white matter hyperintensities (WMH) from MRI scans. This section provides a brief description of the pipeline's workflow, QC procedures and its application to UK Biobank data.

S1.1 Pipeline Overview

UBO Detector is a cluster-based WMH segmentation toolbox that uses a k-nearest neighbours (k-NN) algorithm and information from T1-weighted and FLAIR images. The pipeline consists of the following key steps:

1) Pre-processing:
   - Coregistration of FLAIR to T1 images
   - Tissue segmentation (Grey Matter/White Matter/Cerebrospinal Fluid) of T1 images
   - Spatially normalisation of T1 and FLAIR scans to a standard template (DARTEL warping)
   - Non-brain tissue removal for both registered and normalised T1 and FLAIR images

2) Candidate cluster generation: FMRIB's Automated Segmentation Tool (FAST) is used to segment FLAIR images into three classes, with hyperintense regions identified as potential WMH clusters.

3) WMH classification: A supervised k-NN machine learning algorithm classifies candidate clusters as either WMH or non-WMH based on:
   - Intensity features (ratios of cluster intensity to mean GM/WM intensity)
   - Anatomical location features (tissue probability and distance from ventricles)

- Cluster size features (log-transformed cluster size)

4) Post-processing: WMH maps are segmented into sub-regions, including periventricular, deep, lobar regions and arterial territories (see Table S1).

S1.2 Quality Control Procedures

To ensure the integrity of the WMH extraction, all UBO Detector outputs underwent comprehensive visual quality control procedures. The rigorous QC process included three critical steps: (1) ensuring accurate alignment between T1 and FLAIR images to verify proper coregistration; (2) verifying the accuracy of white matter, grey matter, and CSF tissue segmentation which is crucial for proper WMH detection; and (3) carefully examining the final WMH maps overlaid on original FLAIR images (as shown in Figure S8 below) to assess segmentation quality. For each subject, we visually confirmed that all hyperintense regions corresponding to WMH were correctly identified, non-WMH hyperintensities were not incorrectly labelled as WMH, and that no significant WMH regions were missed by the pipeline. This thorough visual inspection process ensured the reliability of the extracted WMH volumes used in subsequent analyses.

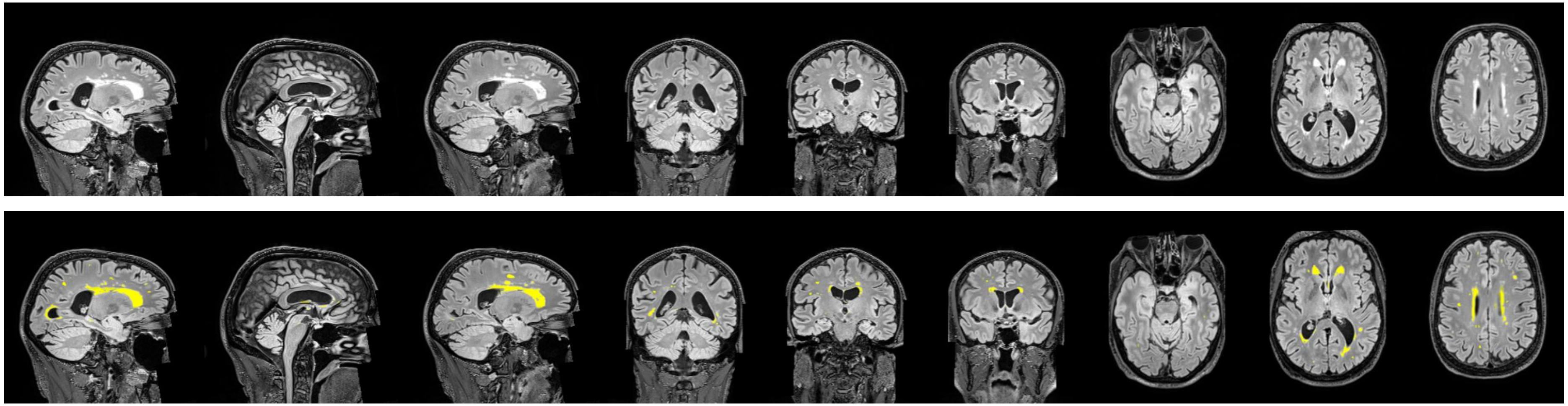

Figure S10 Visual QC of White Matter Hyperintensity (WMH) on FLAIR Images

S1.3 Application to UK Biobank Data

While the UBO Detector was originally validated on Australian cohorts (OATS and Sydney MAS), several factors support its reliability in processing the heterogeneous UK Biobank data, which includes both normotensive and hypertensive participants. First, UBO Detector has demonstrated robust performance across different dataset and scanner types in previous studies (Elliott et al., 2021; Hotz et al., 2022; Richmond-Rakerd et al., 2021; Wang et al., 2021). The UK Biobank imaging data is acquired using standardised protocols across all imaging centres (Siemens Skyra 3T scanners). This consistency in scanner type and imaging protocol significantly reduces scanner-related variability, enhancing the reliability of the results compared to studies using multiple scanner types. Second, the feature space used in UBO Detector (anatomical location, intensity, and cluster size) is designed to be generalizable across different populations, including those with varying degrees of vascular pathology. A recent comparative study by Hotz et al. (2022), evaluating three distinct WMH extraction pipelines, confirmed that UBO Detector shows "excellent volumetric agreement with manually segmented WMH" and "high correlations with the Fazekas scores", validating its effectiveness in capturing clinically relevant WMH patterns across populations with varying WMH burden. Third, UBO Detector provides options to use age-appropriate templates, which we employed to ensure optimal registration for our UK Biobank sample. This feature is particularly valuable when analysing cohorts with a diverse age range, as it helps account for age-related structural differences that might otherwise affect segmentation accuracy.

S2 Hyperparameter Optimisation with Optuna

S2.1 Optuna Framework Overview

The Optuna framework (Akiba et al., 2019) was employed for hyperparameter optimisation of our cVAE-based normative model. Unlike traditional grid search which evaluates all possible combinations of parameters or random search which samples configurations without learning, Optuna implements an intelligent sequential model-based optimization (SMBO) approach. At the core of Optuna is a tree-structured Parzen estimator (TPE), a Bayesian optimisation algorithm that learns from previous evaluations to guide the search process efficiently.

The TPE algorithm maintains two probability density functions - one for parameter sets that yielded good performance and another for those that performed poorly. This allows the algorithm to adaptively sample the parameter space, focusing computational resources on promising regions rather than exploring uniformly.

S2.2 Optimisation Process

We conducted hyperparameter optimisation using the Optuna framework, performing 5-fold cross-validation on the training dataset to ensure robust estimation of model performance. For each fold, 20 Optuna trials were executed, resulting in a total of 100 trials (20 trials × 5 folds). In each trial, Optuna proposed a unique combination of hyperparameters to construct and train a cVAE model, with the mean cross-validation loss as the optimisation objective.

Each model was trained using the Adam optimiser with a learning rate suggested by Optuna. To improve convergence and prevent overfitting, a ReduceLROnPlateau scheduler was implemented to halve the learning rate after 10 epochs without improvement, combined with early stopping using a patience of 20 epochs, based on the validation loss. To enhance computational efficiency, we applied Optuna's Median Pruner, an adaptive pruning mechanism that terminates underperforming trials early. The Median Pruner was configured to begin evaluation after 5 start-up trials and 20 warm-up steps, pruning trials whose intermediate performance fell below the median of prior trials at the same iteration.

Throughout the optimisation process, model checkpoints were automatically saved whenever a new lowest validation loss was achieved. After completion of

all 100 trials, the best-performing hyperparameter configuration was selected based on the average cross-validation loss. Using these optimal hyperparameters, the final cVAE model was retrained on the entire training dataset, reserving 10% of the data as a validation set for monitoring. The model exhibiting the lowest validation loss during this final training phase was retained for subsequent inference analyses.

S2.3 Hyperparameter Search Space

We systematically explored the following hyperparameters:

1. **Latent Space Dimensionality**: [16, 32, 64, 128]
2. **Hidden Layer Dimensions**:
    - Various architectures were explored using a string representation (e.g., "128_64" for two layers with 128 and 64 units):
        - Single hidden layer: "128", "256", "512", "1024"
        - Two hidden layers: "256_128", "128_64", "512_256", "1024_512"
        - Three hidden layers: "256_128_64", "512_256_128", "1024_512_256"
3. **Learning Rate**: Log-uniform sampling between 1e-5 and 1e-2
4. **Batch Size**: [32, 64, 128]

S2.4 Optimisation Results

After completing all trials, the optimal configuration was determined to be:

- Latent space dimension: 64
- Hidden layer size: single hidden layer of 512 units
- Learning rate: 1e-3
- Batch size: 32

This results in our final model, which is described in detail in Section 2.4.1 of the main manuscript.

S3 Analysis of Training and Validation Loss Plot

Figure S9 below shows the training (left) and validation (right) loss components during the training of a conditional Variational Autoencoder (cVAE) model across epochs.

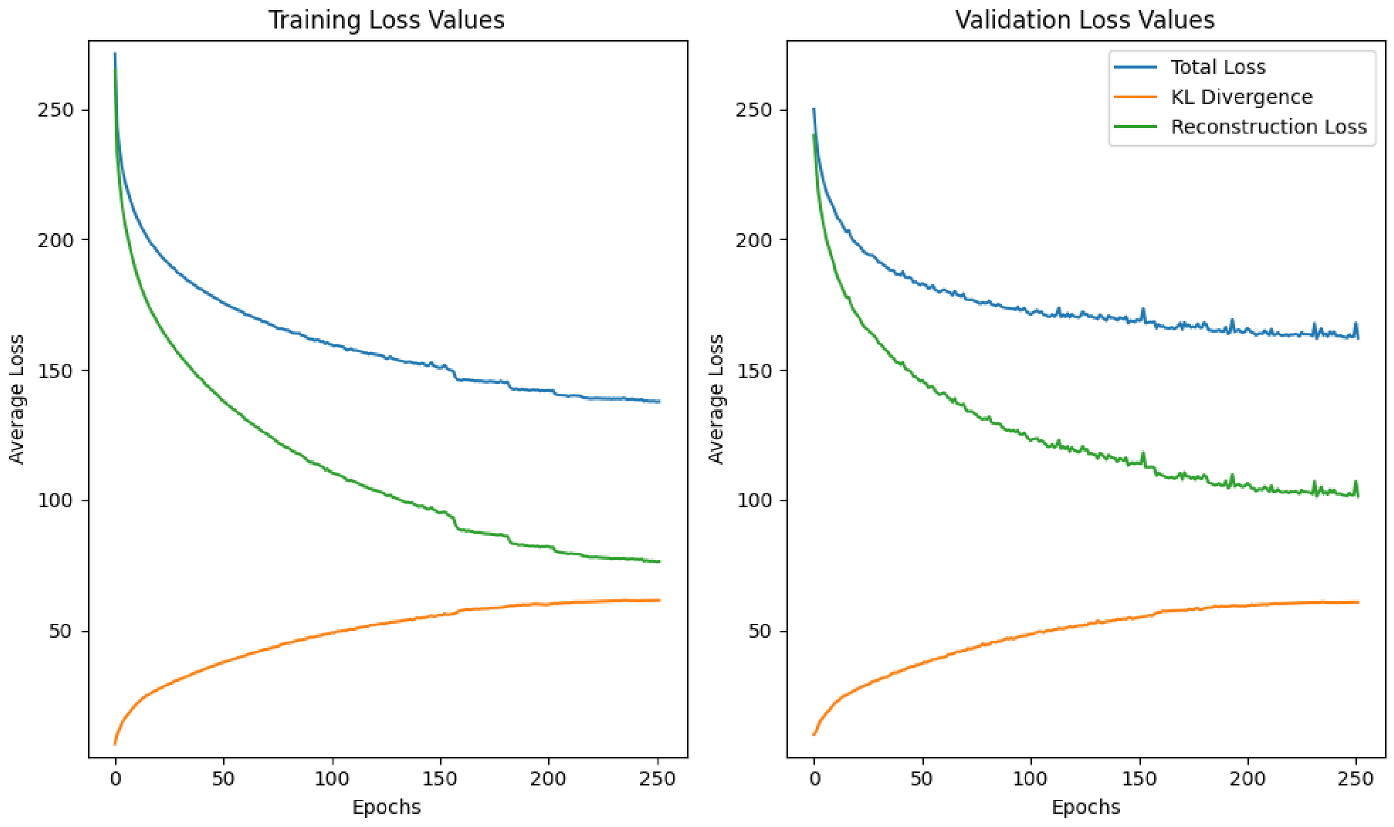

Figure S11 Training and Validation Loss Components (Total Loss, Kullback-Leibler Divergence, Reconstruction Loss) of the cVAE model

Overall, both the total loss and its components (Kullback–Leibler (KL) divergence and negative log-likelihood, representing the reconstruction loss) decrease sharply during the initial training epochs, reflecting rapid convergence. After approximately 50 epochs, the rate of decline slows, and all losses gradually stabilise, indicating that the model has entered a steady learning phase. The training and validation curves follow similar trajectories with only minor gaps, suggesting good generalisation and limited overfitting.

To prevent overfitting, early stopping was applied and triggered at epoch 252. By this point, the total training and validation losses had stabilised at approximately 160, with the negative log-likelihood around 100 and the KL divergence near 60. These correspond to roughly 0.82, 0.51, and 0.31 per input dimension across the 195 features, indicating that the model achieved a well-balanced trade-off between reconstruction accuracy and latent regularisation, with reliable generalisation performance.

S4 Explained Variance as a Performance Metric

Methodology

The formula for calculating the Explained Variance is:

$$EV_j = 1 - \frac{\sum_{i=1}^{N}\left(Y_{ij} - \hat{\mu}_{ij}^{*}\right)^2}{\sum_{i=1}^{N}\left(Y_{ij} - \bar{Y}_j\right)^2}$$

where N is the number of participants, Y_{ij} is the observed value, $\hat{\mu}_{ij}^{*}$ is the predicted mean for participant i and feature j, and $\overline{Y_j}$ is the mean of the observed values for feature j across all participants.

Explained Variance ranges from (-∞, 1], with 1 indicating that the model accounts for 100% of the variance in the observed data. A value of 0 means the model's predictions are no better than simply predicting the mean value, while negative values indicate worse performance than predicting the mean.

Results and Discussion

	**Total Variance ($\sum_{i=1}^{N}\left(Y_{ij} - \bar{Y}_j\right)^2$)**					**Explained Variance**				
	**Prior-cVAE**	**Post-cVAE**	**GAMLSS**	**MFPR**	**HBR**	**Prior-cVAE**	**Post-cVAE**	**GAMLSS**	**MFPR**	**HBR**
**wb_SubCortGray_Vol**	**1051.66**	**1730.31**	**1147.72**	**1144.73**	**1150.13**	**0.43**	**1.00**	**0.48**	**0.48**	**0.48**
wb_3rd-Ventricle_Vol	626.65	1193.02	620.38	714.28	614.41	-0.69	0.69	-0.84	-0.46	-0.70
wb_4th-Ventricle_Vol	270.78	570.37	245.68	257.77	241.53	-4.40	-0.89	-5.08	-4.61	-5.01
wb_5th-Ventricle_Vol	0.00	0.00	0.00	1.28	0.00	<-1e10	<-1e08	-1e09	-1094.27	-1e08
lh_Accumbens-area_Vol	435.13	1073.69	523.93	546.40	494.43	-1.87	0.52	-1.49	-1.28	-1.53
rh_Accumbens-area_Vol	393.27	1062.50	465.41	489.73	477.77	-2.28	0.52	-1.85	-1.62	-1.70
lh_Amygdala_Vol	532.85	1123.98	629.22	637.45	629.82	-1.22	0.56	-0.90	-0.82	-0.85
rh_Amygdala_Vol	641.29	1205.33	715.01	728.68	716.66	-0.62	0.65	-0.44	-0.40	-0.43

wb_Brain-Stem_Vol	841.03	1434.15	819.94	843.55	822.85	-0.06	0.83	-0.11	-0.05	-0.08
wb_CC-Anterior_Vol	301.64	982.02	297.01	331.92	297.94	-3.50	0.37	-3.78	-3.07	-3.57
wb_CC-Central_Vol	115.98	1072.96	91.94	171.76	78.16	-13.18	0.62	-22.15	-8.49	-20.15
wb_CC-Mid-Anterior_Vol	163.22	990.28	136.20	237.53	125.82	-8.64	0.51	-14.55	-5.59	-11.52
wb_CC-Mid-Posterior_Vol	158.81	973.82	161.76	171.90	156.83	-8.77	0.42	-8.61	-7.99	-8.92
wb_CC-Posterior_Vol	206.64	1014.22	204.86	213.96	212.65	-6.16	0.44	-6.25	-5.92	-5.93
wb_CSF_Vol	418.57	820.65	357.80	470.91	361.42	-2.13	-0.01	-3.37	-1.76	-2.65
lh_Caudate_Vol	437.67	1474.04	455.04	475.24	452.76	-1.88	0.94	-1.86	-1.65	-1.79
rh_Caudate_Vol	477.33	1482.59	498.39	519.77	493.27	-1.68	0.93	-1.65	-1.46	-1.59
lh_Cerebellum-Cortex_Vol	638.55	1543.06	656.15	669.54	655.25	-0.73	0.95	-0.68	-0.64	-0.68
rh_Cerebellum-Cortex_Vol	683.66	1567.90	694.93	708.30	692.03	-0.54	0.96	-0.51	-0.48	-0.51
lh_Cerebellum-White-Matter_Vol	519.73	1350.49	548.81	571.67	527.85	-1.22	0.86	-1.14	-1.00	-1.17
rh_Cerebellum-White-Matter_Vol	434.55	1147.53	451.62	456.39	468.67	-1.70	0.78	-1.75	-1.55	-1.50
**lh_CerebralWhiteMatter_Vol**	**1190.14**	**1686.68**	**1282.75**	**1283.36**	**1286.73**	**0.64**	**0.99**	**0.67**	**0.67**	**0.67**
**rh_CerebralWhiteMatter_Vol**	**1196.51**	**1689.10**	**1287.62**	**1285.27**	**1284.31**	**0.63**	**0.98**	**0.67**	**0.67**	**0.66**
lh_Hippocampus_Vol	564.75	1437.83	647.02	657.05	635.00	-1.02	0.85	-0.74	-0.72	-0.77
rh_Hippocampus_Vol	554.25	1419.24	646.50	649.88	649.90	-0.93	0.88	-0.64	-0.63	-0.63
lh_Inf-Lat-Vent_Vol	364.98	932.07	452.17	452.08	444.43	-2.62	0.26	-1.87	-1.87	-1.92
rh_Inf-Lat-Vent_Vol	276.93	723.97	314.42	321.52	308.44	-4.40	-0.30	-3.75	-3.61	-3.84
lh_Lateral-Ventricle_Vol	488.93	1648.45	578.51	580.55	578.80	-1.48	0.97	-1.09	-1.07	-1.08
rh_Lateral-Ventricle_Vol	513.68	1637.23	589.36	588.95	588.57	-1.38	0.97	-1.06	-1.05	-1.04
lh_Pallidum_Vol	642.23	1263.89	671.43	675.25	690.69	-0.60	0.74	-0.56	-0.51	-0.47
rh_Pallidum_Vol	672.79	1297.94	704.39	714.12	729.68	-0.55	0.75	-0.52	-0.45	-0.41
lh_Putamen_Vol	569.61	1477.10	640.77	642.57	626.01	-0.85	0.94	-0.64	-0.63	-0.67
rh_Putamen_Vol	559.38	1467.11	628.59	630.20	634.19	-0.99	0.93	-0.78	-0.76	-0.76
lh_Thalamus-Proper_Vol	811.39	1572.51	833.24	887.45	872.47	-0.14	0.92	-0.17	-0.04	-0.06
rh_Thalamus-Proper_Vol	905.79	1576.29	941.82	966.84	964.36	0.05	0.91	0.06	0.10	0.10
lh_VentralDC_Vol	911.36	1550.92	934.21	946.80	912.62	0.07	0.92	0.08	0.11	0.08
rh_VentralDC_Vol	906.68	1529.43	918.16	937.37	926.10	0.10	0.92	0.10	0.13	0.12

wb_VentricleChoroid_Vol	536.18	1695.77	625.79	624.84	624.77	-1.21	0.99	-0.87	-0.87	-0.87
lh_caudalanteriorcingulate_Thick	24.31	918.63	35.92	38.51	37.00	-67.20	0.40	-52.46	-42.06	-44.06
rh_caudalanteriorcingulate_Thick	36.65	843.55	37.25	39.86	28.16	-45.95	0.29	-48.07	-42.22	-60.31
lh_caudalmiddlefrontal_Thick	85.35	1177.01	139.68	148.53	145.83	-17.58	0.61	-10.38	-9.62	-9.84
rh_caudalmiddlefrontal_Thick	88.65	1206.29	137.12	142.80	147.57	-16.74	0.64	-10.40	-9.89	-9.55
lh_cuneus_Thick	20.85	1153.49	37.57	37.53	38.10	-77.59	0.72	-42.92	-42.88	-42.31
rh_cuneus_Thick	17.96	1250.49	25.32	25.43	27.75	-97.29	0.74	-69.51	-68.63	-62.91
lh_entorhinal_Thick	15.64	653.28	52.07	72.65	59.65	-110.98	-0.27	-33.32	-22.86	-28.17
rh_entorhinal_Thick	11.65	651.74	35.59	43.92	26.33	-145.15	-0.18	-47.33	-37.33	-63.25
lh_fusiform_Thick	50.78	1059.48	92.10	98.73	90.43	-32.36	0.45	-17.76	-16.23	-17.86
rh_fusiform_Thick	65.64	1047.29	101.02	106.98	94.10	-23.72	0.49	-15.40	-14.18	-16.23
lh_inferiorparietal_Thick	117.10	1249.53	192.69	197.39	177.14	-12.53	0.69	-7.23	-7.04	-7.98
rh_inferiorparietal_Thick	130.13	1289.58	192.57	202.30	195.61	-10.95	0.73	-7.06	-6.65	-6.88
lh_inferiortemporal_Thick	27.36	1009.78	47.45	48.89	40.34	-61.63	0.42	-35.16	-33.98	-41.53
rh_inferiortemporal_Thick	29.79	988.87	57.62	58.53	42.35	-54.85	0.43	-27.84	-27.38	-38.19
lh_insula_Thick	20.01	886.35	48.16	51.50	42.07	-79.43	0.29	-32.93	-30.40	-37.33
rh_insula_Thick	39.43	927.25	53.06	58.22	54.60	-40.76	0.30	-30.26	-27.22	-29.14
lh_isthmuscingulate_Thick	56.98	652.20	58.43	61.32	56.19	-29.02	-0.26	-29.87	-26.97	-29.57
rh_isthmuscingulate_Thick	62.41	720.34	73.89	75.31	73.96	-26.94	-0.01	-23.51	-22.19	-22.64
lh_lateraloccipital_Thick	45.09	1252.54	70.39	75.41	64.87	-36.77	0.75	-23.24	-21.60	-25.26
rh_lateraloccipital_Thick	52.82	1275.92	73.36	75.68	72.54	-30.30	0.75	-21.57	-20.88	-21.83
lh_lateralorbitofrontal_Thick	5.33	872.43	25.14	28.27	18.57	-322.50	0.21	-67.79	-59.98	-92.37
rh_lateralorbitofrontal_Thick	18.75	871.65	25.90	27.09	29.20	-88.80	0.14	-64.03	-61.03	-56.58
lh_lingual_Thick	51.16	1218.15	61.03	61.44	64.98	-30.41	0.79	-25.59	-25.24	-23.85
rh_lingual_Thick	21.68	1312.82	26.48	26.83	26.51	-80.37	0.78	-65.26	-64.44	-65.42
lh_medialorbitofrontal_Thick	16.99	793.47	39.49	40.12	38.44	-102.07	0.02	-43.52	-42.62	-44.44
rh_medialorbitofrontal_Thick	25.06	725.71	38.90	36.39	26.92	-69.35	-0.10	-44.74	-47.37	-64.34
lh_middletemporal_Thick	49.27	1172.06	78.80	82.88	75.88	-33.39	0.64	-21.43	-19.43	-21.34
rh_middletemporal_Thick	65.02	1109.62	87.21	86.33	94.33	-25.68	0.58	-19.06	-19.06	-17.40

lh_paracentral_Thick	84.25	1168.25	136.33	154.99	129.41	-17.72	0.72	-10.94	-9.19	-11.20
rh_paracentral_Thick	81.98	1180.37	119.70	137.82	129.42	-18.67	0.68	-12.83	-10.70	-11.49
lh_parahippocampal_Thick	42.02	1007.07	65.57	68.51	70.18	-39.84	0.58	-25.81	-24.23	-23.65
rh_parahippocampal_Thick	50.85	1021.58	63.13	64.31	48.01	-29.91	0.57	-24.11	-23.27	-31.47
lh_parsopercularis_Thick	85.12	941.70	129.05	134.56	126.81	-17.13	0.32	-10.99	-10.45	-11.21
rh_parsopercularis_Thick	90.89	939.72	128.82	133.48	116.99	-16.85	0.24	-11.54	-11.07	-12.78
lh_parsorbitalis_Thick	40.72	722.63	75.46	77.73	70.35	-35.77	-0.01	-18.94	-18.22	-20.36
rh_parsorbitalis_Thick	56.44	767.18	96.86	99.19	102.47	-28.52	-0.01	-16.29	-15.78	-15.30
lh_parstriangularis_Thick	104.73	965.91	148.79	166.29	150.99	-13.86	0.33	-9.44	-8.37	-9.36
rh_parstriangularis_Thick	106.21	933.75	156.07	155.32	178.43	-13.51	0.34	-8.94	-8.93	-7.66
lh_pericalcarine_Thick	20.81	970.84	22.17	22.68	10.77	-78.92	0.49	-75.60	-72.49	-152.88
rh_pericalcarine_Thick	16.49	1084.75	11.53	12.18	12.93	-109.45	0.60	-161.19	-148.94	-140.10
lh_postcentral_Thick	81.42	1304.69	129.17	137.60	120.10	-18.29	0.80	-11.20	-10.43	-12.11
rh_postcentral_Thick	88.98	1298.88	130.55	134.22	129.21	-16.82	0.85	-11.04	-10.70	-11.20
lh_posteriorcingulate_Thick	33.99	787.51	51.50	55.35	58.06	-47.27	0.09	-30.89	-28.67	-27.23
rh_posteriorcingulate_Thick	67.05	794.44	78.80	84.35	93.24	-23.02	0.13	-19.46	-18.13	-16.37
lh_precentral_Thick	86.21	1298.72	136.10	158.95	119.92	-16.81	0.80	-10.94	-8.62	-11.77
rh_precentral_Thick	103.44	1346.39	144.63	165.44	140.73	-14.38	0.84	-10.79	-8.56	-10.27
lh_precuneus_Thick	112.59	1267.35	185.88	192.02	179.41	-13.52	0.73	-7.77	-7.46	-8.05
rh_precuneus_Thick	114.15	1271.58	177.22	186.94	176.74	-12.52	0.70	-7.75	-7.26	-7.75
lh_rostralanteriorcingulate_Thick	54.04	735.71	91.12	91.73	104.49	-28.52	-0.16	-16.51	-16.41	-14.31
rh_rostralanteriorcingulate_Thick	32.45	598.24	41.01	41.71	39.79	-47.91	-0.58	-37.58	-36.89	-38.73
lh_rostralmiddlefrontal_Thick	112.22	1239.31	197.54	202.97	190.13	-13.29	0.66	-7.11	-6.87	-7.41
rh_rostralmiddlefrontal_Thick	116.88	1221.18	179.11	182.71	174.97	-12.27	0.71	-7.61	-7.45	-7.83
lh_superiorfrontal_Thick	165.29	1401.97	262.00	274.89	260.79	-7.94	0.86	-4.68	-4.39	-4.66
rh_superiorfrontal_Thick	171.50	1424.60	243.50	256.72	256.60	-7.93	0.86	-5.32	-4.95	-4.95
lh_superiorparietal_Thick	101.36	1285.01	171.29	173.08	157.57	-14.87	0.78	-8.40	-8.30	-9.24
rh_superiorparietal_Thick	124.90	1360.18	190.79	194.56	176.30	-11.37	0.82	-7.11	-6.93	-7.78
lh_superiortemporal_Thick	90.83	1338.62	148.86	158.66	145.46	-16.84	0.78	-10.06	-9.15	-10.10

rh_superiortemporal_Thick	140.34	1296.74	196.06	207.79	186.34	-10.27	0.76	-7.22	-6.61	-7.46
lh_supramarginal_Thick	106.77	1186.74	167.26	174.23	161.28	-13.21	0.63	-8.06	-7.67	-8.42
rh_supramarginal_Thick	103.31	1202.00	158.59	166.78	158.47	-14.28	0.64	-9.02	-8.45	-8.97
lh_transversetemporal_Thick	13.35	802.39	5.54	5.16	4.85	-133.72	0.02	-322.60	-347.21	-369.60
rh_transversetemporal_Thick	11.36	814.55	5.51	7.34	4.97	-148.87	0.27	-312.33	-230.18	-340.79
**wb_TotalGray_Vol**	**1189.03**	**1662.06**	**1369.21**	**1349.46**	**1366.98**	**0.70**	**1.00**	**0.74**	**0.74**	**0.74**
**lh_Cortex_Vol**	**1049.43**	**1654.94**	**1252.92**	**1224.17**	**1237.55**	**0.56**	**1.00**	**0.63**	**0.63**	**0.63**
**rh_Cortex_Vol**	**1062.63**	**1656.05**	**1260.22**	**1234.66**	**1227.65**	**0.55**	**1.00**	**0.62**	**0.62**	**0.61**
lh_caudalanteriorcingulate_Vol	374.18	1086.15	446.03	464.03	466.48	-2.40	0.62	-1.85	-1.72	-1.71
rh_caudalanteriorcingulate_Vol	115.26	872.64	137.62	150.85	133.40	-12.88	0.50	-10.58	-9.56	-10.90
lh_caudalmiddlefrontal_Vol	451.32	934.64	519.31	541.66	512.12	-1.45	0.45	-1.14	-1.03	-1.15
rh_caudalmiddlefrontal_Vol	428.13	942.18	486.75	513.48	475.06	-1.74	0.42	-1.42	-1.28	-1.45
lh_cuneus_Vol	287.54	1101.79	292.52	335.03	284.74	-3.60	0.68	-3.94	-2.94	-3.65
rh_cuneus_Vol	331.27	1202.30	337.93	390.26	323.84	-2.76	0.77	-2.95	-2.19	-2.83
lh_entorhinal_Vol	163.44	750.91	157.76	213.61	163.89	-7.44	0.23	-8.55	-5.42	-7.40
rh_entorhinal_Vol	162.86	747.36	146.77	183.74	141.09	-8.03	0.13	-9.76	-6.95	-9.36
lh_fusiform_Vol	539.12	966.97	575.79	602.97	563.40	-0.95	0.40	-0.86	-0.74	-0.87
rh_fusiform_Vol	598.75	1029.49	631.42	663.38	645.79	-0.67	0.52	-0.61	-0.50	-0.55
lh_inferiorparietal_Vol	490.97	1115.09	540.14	563.57	540.26	-1.27	0.61	-1.10	-0.96	-1.05
rh_inferiorparietal_Vol	586.58	1154.83	640.32	662.66	622.39	-0.70	0.63	-0.56	-0.49	-0.60
lh_inferiortemporal_Vol	592.69	1124.02	638.70	677.27	659.98	-0.67	0.61	-0.59	-0.45	-0.49
rh_inferiortemporal_Vol	655.73	1164.94	720.13	734.56	709.74	-0.51	0.66	-0.38	-0.34	-0.39
lh_insula_Vol	633.92	1394.47	717.73	739.39	686.05	-0.49	0.89	-0.33	-0.26	-0.37
rh_insula_Vol	643.36	1388.51	725.59	753.74	724.97	-0.41	0.90	-0.26	-0.19	-0.24
lh_isthmuscingulate_Vol	483.13	912.57	498.75	563.12	505.32	-1.41	0.28	-1.51	-1.06	-1.30
rh_isthmuscingulate_Vol	415.16	877.18	433.14	479.94	427.92	-2.34	0.20	-2.38	-1.89	-2.25
lh_lateraloccipital_Vol	525.12	1196.05	577.86	602.81	589.31	-1.02	0.75	-0.90	-0.76	-0.79
rh_lateraloccipital_Vol	560.70	1229.05	582.26	619.75	582.30	-0.99	0.74	-0.98	-0.80	-0.91
lh_lateralorbitofrontal_Vol	719.18	1328.69	832.34	844.88	847.37	-0.25	0.78	-0.10	-0.07	-0.07

rh_lateralorbitofrontal_Vol	709.59	1296.02	824.15	828.77	816.25	-0.24	0.75	-0.07	-0.06	-0.08
lh_lingual_Vol	293.04	1129.56	318.60	330.47	314.35	-3.34	0.78	-3.02	-2.85	-3.07
rh_lingual_Vol	272.32	1204.87	300.80	306.91	292.28	-4.18	0.78	-3.70	-3.59	-3.84
lh_medialorbitofrontal_Vol	558.14	1108.03	645.05	649.51	648.28	-1.02	0.52	-0.75	-0.73	-0.73
rh_medialorbitofrontal_Vol	516.02	1052.00	602.19	605.84	626.17	-1.21	0.51	-0.90	-0.88	-0.82
lh_middletemporal_Vol	672.33	1368.11	738.43	752.29	729.67	-0.41	0.79	-0.28	-0.25	-0.29
rh_middletemporal_Vol	684.55	1295.27	769.76	791.53	746.92	-0.40	0.76	-0.24	-0.20	-0.27
lh_paracentral_Vol	415.55	998.11	486.57	515.24	471.68	-1.71	0.51	-1.34	-1.16	-1.37
rh_paracentral_Vol	407.85	978.82	448.64	478.50	477.03	-2.11	0.48	-1.87	-1.64	-1.66
lh_parahippocampal_Vol	126.52	954.08	160.07	182.73	152.26	-10.91	0.54	-9.11	-7.28	-8.91
rh_parahippocampal_Vol	142.59	864.08	182.13	187.05	175.06	-10.07	0.26	-7.98	-7.37	-7.96
lh_parsopercularis_Vol	320.69	803.06	317.75	389.75	319.51	-2.92	0.22	-3.32	-2.21	-2.94
rh_parsopercularis_Vol	327.88	787.54	344.75	412.62	334.88	-2.85	0.13	-2.95	-2.05	-2.75
lh_parsorbitalis_Vol	324.78	892.05	369.70	394.86	397.93	-2.91	0.37	-2.49	-2.22	-2.22
rh_parsorbitalis_Vol	326.51	889.44	357.59	385.57	369.14	-3.09	0.35	-2.85	-2.43	-2.60
lh_parstriangularis_Vol	303.25	874.44	306.71	359.79	316.14	-3.21	0.47	-3.37	-2.54	-3.03
rh_parstriangularis_Vol	298.37	717.41	284.84	343.07	274.58	-3.49	-0.02	-4.29	-2.91	-3.89
lh_pericalcarine_Vol	189.81	1166.89	171.68	213.34	168.77	-6.52	0.83	-8.74	-5.66	-7.43
rh_pericalcarine_Vol	203.41	1242.51	170.38	220.16	164.89	-6.21	0.87	-9.14	-5.65	-7.88
lh_postcentral_Vol	533.90	1216.80	612.90	633.64	611.20	-0.94	0.73	-0.72	-0.63	-0.70
rh_postcentral_Vol	476.81	1247.87	567.95	589.97	579.44	-1.48	0.77	-1.12	-0.98	-1.02
lh_posteriorcingulate_Vol	431.49	854.11	485.69	492.01	451.29	-1.82	0.16	-1.49	-1.46	-1.69
rh_posteriorcingulate_Vol	404.23	873.48	492.52	496.50	475.80	-2.23	0.11	-1.63	-1.61	-1.72
lh_precentral_Vol	607.28	1230.17	696.87	702.39	701.67	-0.60	0.77	-0.39	-0.38	-0.38
rh_precentral_Vol	580.27	1222.26	672.61	674.08	677.11	-0.75	0.78	-0.52	-0.51	-0.51
lh_precuneus_Vol	624.79	1297.34	736.82	755.87	749.18	-0.58	0.80	-0.33	-0.29	-0.30
rh_precuneus_Vol	705.44	1319.67	799.04	824.36	797.43	-0.41	0.77	-0.25	-0.20	-0.24
lh_rostralanteriorcingulate_Vol	538.36	1004.56	632.02	651.91	630.65	-1.05	0.39	-0.76	-0.69	-0.75
rh_rostralanteriorcingulate_Vol	336.60	792.98	381.43	401.72	400.14	-2.94	0.05	-2.52	-2.30	-2.30

lh_rostralmiddlefrontal_Vol	652.22	1133.00	715.69	778.93	719.03	-0.41	0.65	-0.34	-0.18	-0.27
rh_rostralmiddlefrontal_Vol	621.01	1144.36	673.31	725.56	682.82	-0.51	0.69	-0.47	-0.28	-0.36
lh_superiorfrontal_Vol	776.48	1296.15	887.03	900.96	886.43	0.00	0.81	0.11	0.14	0.12
rh_superiorfrontal_Vol	813.55	1379.14	906.66	928.39	929.94	0.10	0.87	0.19	0.22	0.22
lh_superiorparietal_Vol	430.28	1075.27	524.82	543.39	531.45	-1.76	0.60	-1.27	-1.19	-1.24
rh_superiorparietal_Vol	447.51	1104.27	541.18	552.98	546.64	-1.64	0.60	-1.17	-1.13	-1.15
lh_superiortemporal_Vol	645.54	1320.19	721.79	736.89	694.42	-0.48	0.80	-0.33	-0.29	-0.37
rh_superiortemporal_Vol	660.95	1331.85	765.74	781.87	726.78	-0.45	0.79	-0.26	-0.22	-0.32
lh_supramarginal_Vol	572.16	1030.79	601.16	647.29	597.13	-1.05	0.42	-0.99	-0.81	-0.97
rh_supramarginal_Vol	538.24	1088.29	618.46	641.88	622.50	-0.90	0.63	-0.66	-0.59	-0.64
lh_transversetemporal_Vol	226.84	755.16	241.38	282.74	222.20	-5.07	0.07	-4.97	-3.85	-5.21
rh_transversetemporal_Vol	277.15	917.02	279.73	341.12	275.62	-3.54	0.53	-3.86	-2.67	-3.53
wholeBrainWMHvol	205.03	1573.47	320.07	384.78	303.15	-5.29	0.99	-3.33	-2.30	-3.19
PVWMHvol	196.29	1570.33	310.58	373.05	298.52	-5.69	0.99	-3.39	-2.44	-3.31
DWMHvol	108.95	1116.43	208.84	231.96	202.55	-11.80	0.77	-5.70	-4.92	-5.78
Lfrontal_WMHvol	32.76	637.29	9.82	105.85	9.19	-46.90	0.00	-216.01	-13.62	-173.53
Rfrontal_WMHvol	35.16	667.21	9.63	105.21	9.57	-42.59	0.11	-212.66	-13.43	-162.51
Ltemporal_WMHvol	41.54	270.52	5.21	72.81	5.55	-40.21	-4.06	-505.65	-22.27	-312.55
Rtemporal_WMHvol	60.23	258.70	5.02	76.52	7.00	-28.04	-4.51	-554.36	-21.65	-251.65
Lparietal_WMHvol	35.03	298.10	19.90	146.96	16.73	-43.46	-1.99	-93.61	-9.08	-92.28
Rparietal_WMHvol	33.09	263.71	23.49	124.29	19.40	-48.11	-2.86	-80.33	-11.56	-81.37
Loccipital_WMHvol	21.76	1614.02	2.73	22.84	4.71	-74.57	0.87	-924.13	-70.44	-347.50
Roccipital_WMHvol	13.66	1328.01	2.65	21.71	4.12	-116.31	0.84	-809.74	-72.93	-392.45
Lcerebellum_WMHvol	0.00	0.00	0.00	8.43	0.03	<-1e08	<-1e06	<-1e09	-213.49	-56959.38
Rcerebellum_WMHvol	0.00	0.00	0.00	11.17	0.00	<-1e08	<-1e06	<-1e09	-177.93	<-1e07
Brainstem_WMHvol	4.19	221.60	2.75	30.91	4.08	-457.40	-3.14	-749.45	-56.04	-436.21
lAAH_WMHvol	23.79	202.83	36.81	80.19	30.02	-56.54	-3.53	-39.95	-15.79	-44.61
rAAH_WMHvol	25.19	266.63	54.35	88.37	47.30	-57.71	-2.70	-27.61	-15.46	-29.85
lMAH_WMHvol	75.72	797.24	170.85	174.31	154.00	-16.50	0.33	-6.72	-6.54	-7.57

rMAH_WMHvol	112.95	1057.23	228.82	241.73	241.44	-11.44	0.64	-5.11	-4.72	-4.73
lAAML_WMHvol	112.99	735.39	204.59	222.66	195.06	-12.08	0.06	-6.82	-5.55	-6.48
rAAML_WMHvol	165.45	880.49	275.04	300.17	259.66	-8.12	0.24	-4.97	-3.90	-4.67
lAAC_WMHvol	79.11	955.44	154.59	150.34	157.26	-21.76	0.23	-11.80	-10.87	-10.41
rAAC_WMHvol	36.47	667.82	76.06	66.05	86.35	-43.86	-0.26	-23.12	-23.56	-17.92
lMALL_WMHvol	176.84	1273.01	324.96	310.25	305.79	-6.70	0.81	-3.32	-3.33	-3.40
rMALL_WMHvol	147.95	1264.95	253.55	276.11	246.27	-8.83	0.72	-4.65	-4.19	-4.83
lPATMP_WMHvol	24.10	423.18	29.22	33.51	25.98	-60.45	-1.05	-54.18	-43.14	-56.02
rPATMP_WMHvol	23.35	479.71	38.76	40.68	41.65	-61.33	-0.66	-39.05	-34.68	-33.81
lPAH_WMHvol	16.50	263.33	23.12	55.48	21.52	-108.20	-3.18	-86.19	-31.26	-82.08
rPAH_WMHvol	21.47	327.16	25.80	59.07	24.42	-72.07	-1.70	-65.91	-25.40	-63.23
lPAC_WMHvol	19.14	0.18	0.00	105.27	0.02	-86.25	-11721.57	<-1e09	-14.36	-98217.41
rPAC_WMHvol	0.00	0.02	0.00	74.20	0.01	<-1e07	<-1e06	<-1e09	-22.55	<-1e06

An interesting pattern emerged when examining Explained Variance across different brain measures. Global measures with high inter-subject variability, such as whole-brain subcortical grey matter volume, yielded positive Explained Variance values across covariate-only models, indicating that these models captured a substantial proportion of the total variance. In contrast, most regional features, including specific subcortical structures and lobar WMH volumes, exhibited negative Explained Variance despite achieving reasonable performance on other metrics such as Median Absolute Error and RMSE. This apparent paradox reflects the mathematical properties of the Explained Variance formula, where the denominator represents the total variance in observed values. Regional features often exhibit relatively low inter-subject variability compared to their prediction errors, particularly in regions with sparse pathology or small anatomical structures. These findings highlight that Explained Variance, whilst useful for assessing global measures with substantial inter-subject variability, may be less informative for evaluating model performance on regional features where baseline variance is inherently low. For such features, metrics like Median Absolute Error and calibration quality provide more meaningful assessments of predictive performance.

Reference

Akiba, T., Sano, S., Yanase, T., Ohta, T., & Koyama, M. (2019). Optuna: A Next-generation Hyperparameter Optimization Framework. *Proceedings of the 25th ACM SIGKDD International Conference on Knowledge Discovery & Data Mining*, 2623–2631. https://doi.org/10.1145/3292500.3330701

Elliott, M. L., Caspi, A., Houts, R. M., Ambler, A., Broadbent, J. M., Hancox, R. J., Harrington, H., Hogan, S., Keenan, R., Knodt, A., Leung, J. H., Melzer, T. R., Purdy, S. C., Ramrakha, S., Richmond-Rakerd, L. S., Righarts, A., Sugden, K., Thomson, W. M., Thorne, P. R., … Moffitt, T. E. (2021). Disparities in the pace of biological aging among midlife adults of the same chronological age have implications for future frailty risk and policy. *Nature Aging*, *1*(3), 295–308. https://doi.org/10.1038/s43587-021-00044-4

Hotz, I., Deschwanden, P. F., Liem, F., Mérillat, S., Malagurski, B., Kollias, S., & Jäncke, L. (2022). Performance of three freely available methods for extracting white matter hyperintensities: FreeSurfer, UBO Detector, and BIANCA. *Human Brain Mapping*, *43*(5), 1481–1500. https://doi.org/10.1002/hbm.25739

Jiang, J., Liu, T., Zhu, W., Koncz, R., Liu, H., Lee, T., Sachdev, P. S., & Wen, W. (2018). UBO Detector – A cluster-based, fully automated pipeline for extracting white matter hyperintensities. *NeuroImage*, *174*, 539–549. https://doi.org/10.1016/j.neuroimage.2018.03.050

Richmond-Rakerd, L. S., Caspi, A., Ambler, A., d'Arbeloff, T., de Bruine, M., Elliott, M., Harrington, H., Hogan, S., Houts, R. M., Ireland, D., Keenan, R., Knodt, A. R., Melzer, T. R., Park, S., Poulton, R., Ramrakha, S., Rasmussen, L. J. H., Sack, E., Schmidt, A. T., … Moffitt, T. E. (2021). Childhood self-control forecasts the pace of midlife aging and preparedness for old age. *Proceedings of the National Academy of Sciences*, *118*(3), e2010211118. https://doi.org/10.1073/pnas.2010211118

Wang, T., Jin, A., Fu, Y., Zhang, Z., Li, S., Wang, D., & Wang, Y. (2021). Heterogeneity of White Matter Hyperintensities in Cognitively Impaired Patients With Cerebral Small Vessel Disease. *Frontiers in Immunology*, *12*. https://doi.org/10.3389/fimmu.2021.803504
